# Binding Analysis and Structure-Based Design of Tricyclic Coumarin-Derived MTHFD2 Inhibitors as Anticancer Agents: Insights from Computational Modeling

**DOI:** 10.1021/acsomega.2c08025

**Published:** 2023-04-12

**Authors:** Vibhu Jha, Fredrik Lannestam Holmelin, Leif A. Eriksson

**Affiliations:** Department of Chemistry and Molecular Biology, University of Gothenburg, 405 30 Göteborg, Sweden

## Abstract

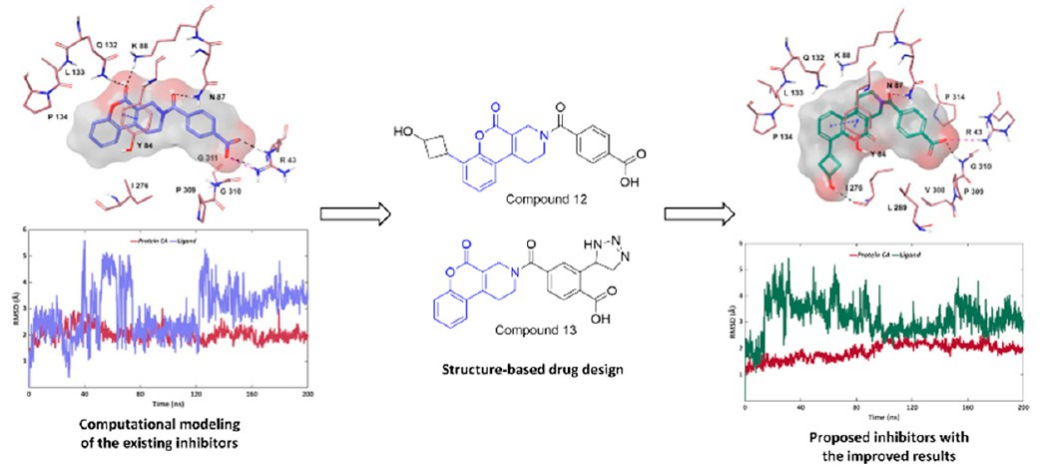

Unfolded protein response (UPR)-dependent metabolic reprogramming diverts metabolites from glycolysis to mitochondrial 1C metabolism, highlighting pharmacological resistance to folate drugs and overexpression of certain enzymes. Methylenetetrahydrofolate dehydrogenase (MTHFD2) is a mitochondrial enzyme that plays a key role in 1C metabolism in purine and thymidine synthesis and is exclusively overexpressed in cancer cells but absent in most healthy adult human tissues. To the best of our knowledge, tricyclic coumarin-based compounds (substrate site binders) and xanthine derivatives (allosteric site binders) are the only selective inhibitors of MTHFD2 reported until the present date. The current study aims at the investigation of the available structural data of MTHFD2 in complex with potent and selective inhibitors that occupy the substrate binding site, further providing insights into binding mode, key protein–ligand interactions, and conformational dynamics, that correspond to the experimental binding affinities and biological activities. In addition, we carried out structure-based drug design on the substrate binding site of MTHFD2, by exploiting the cocrystal structure of MTHFD2 with the tricyclic coumarin-based inhibitor. The structure-based drug design campaign involves R-group enumeration, bioisostere replacement, molecular docking, ADME prediction, MM-GBSA binding free energy calculations, and molecular dynamics simulations, that led to a small library of new and potential compounds, capable of selectively inhibiting MTHFD2. The results reported herein are expected to benefit medicinal chemists working on the development of selective MTHFD2 inhibitors for cancer treatment, although experimental validation by biochemical and/or pharmacokinetic assays is required to substantiate the outcomes of the study.

## 1 Introduction

**Figure 1:**
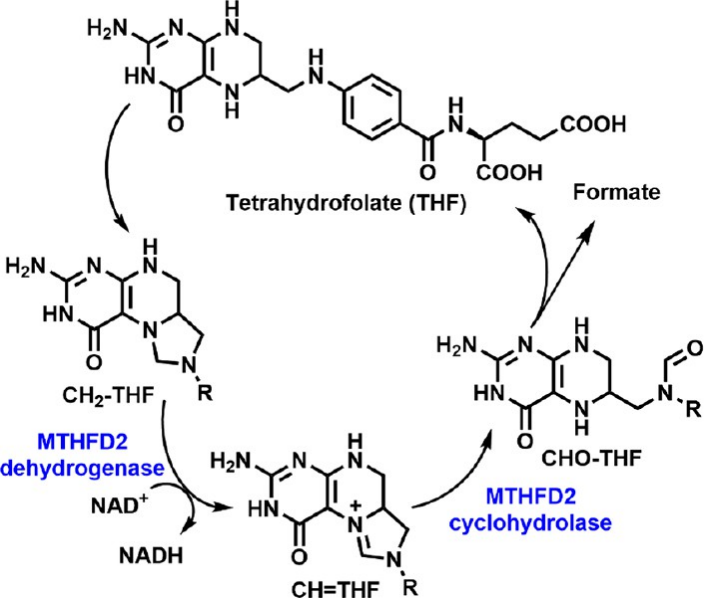
Mitochondrial MTHFD2 in the folate pathway.

In 2019, the WHO estimated cancer as one of the two leading causes of death before the age of 70 in 112 of 183 countries.^1^ An estimated 19.3 million new cancer cases and almost 10 million cancer deaths occurred globally in 2020,^2^ with female breast cancer being the most diagnosed cancer (2.26 million cases worldwide).^3^ By 2025, 4.3 million new cancer cases annually in Europe and 20 million new cases globally are anticipated.^4^ New and more effective ways of cancer treatment are hence urgently needed. A multiomics analysis revealed that one of the stress response pathways, the endoplasmic reticulum (ER) unfolded protein response (UPR), diverts the metabolites from glycolysis to mitochondrial 1C metabolism, which results in cellular insensitivity to FDA-approved antimetabolites such as Methotrexate and Pemetrexed, establishing a direct link to drug resistance.^5^ UPR-driven changes lead to differential expression of certain enzymes in cancer. In particular, the bifunctional mitochondrial methylenetetrahydrofolate dehydrogenase/cyclohydrolase (MTHFD2), that plays a key role in 1C metabolism in purine and thymidine synthesis, was identified as one of the overexpressed enzymes.^6^ MTHFD2 catalyzes dehydrogenation of 5,10-methylene-THF (CH2–THF) with an NAD^+^ cofactor, and cyclohydrolysis of 5,10-methenyl-THF (CH=THF), to yield 10-formyl-THF (CHO–THF), eventually producing formate as a 1C unit^7^ (Figure 1).

**Figure 2:**
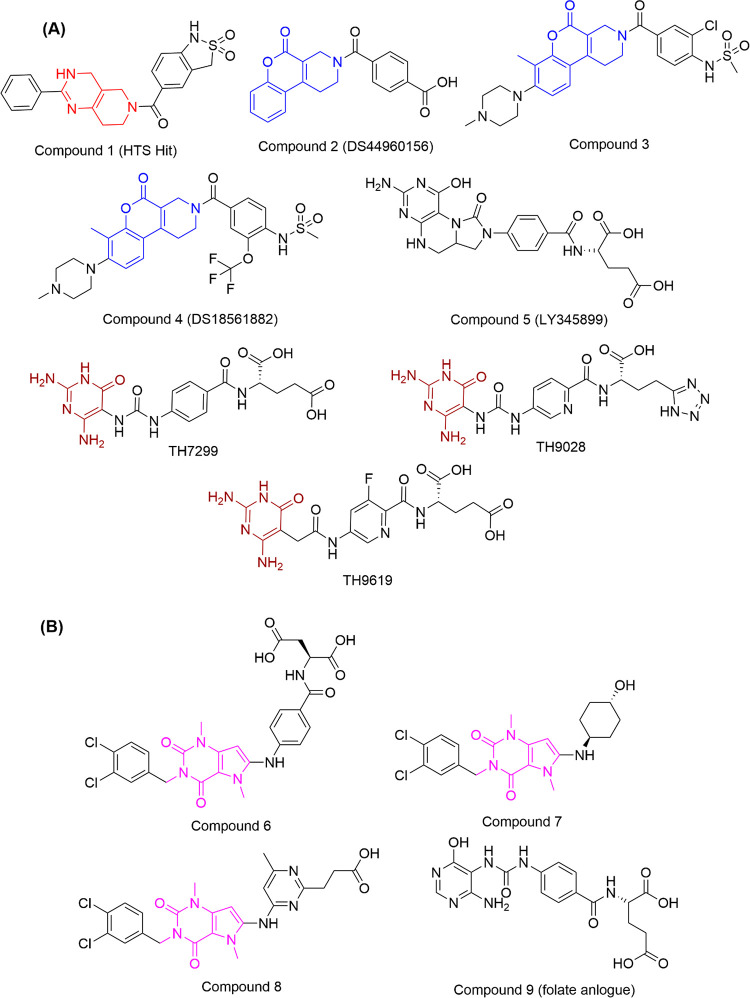
(A) Cocrystallized/reported substrate site inhibitors of MTHFD2 (compounds **1**–**4**, TH7299, TH9028, and TH9619) and MTHFD1 (compound **5**). (B) Cocrystallized/reported MTHFD2 allosteric inhibitors (compounds **6**–**8**) and folate-based inhibitor (compound **9**).

MTHFD2 has gained broad attention due to its high expression level in tumors associated with poor prognosis and low survival rate (in particular breast and colorectal cancers).^8,9^ Interestingly, most healthy adult tissues do not express MTHFD2 but instead the close homologue MTHFD1. Thus, the development of selective MTHFD2 inhibitors could present a novel and promising therapeutic strategy for MTHFD2-overexpressing cancers with minimal side effects.^10^ Despite notable expression of MTHFD2 in various cancer types, only a few MTHFD1/2 dual inhibitors have been identified. A folate analogue LY345899^7,10^ (here denoted as compound **5**, Figure 2A) inhibited both MTHFD2 (IC_50_: 663 nM) and MTHFD1 (IC_50_: 96 nM) and suppressed tumor growth in a mice xenograft model of colorectal cancer through intraperitoneal injection.^7^ Similarly, a natural product named carolacton was identified, that binds to both MTHFD1 and MTHFD2 with *K*_i_ values in the nanomolar range.^11^ These potent compounds concurrently inhibit MTHFD1/2; however, the inhibition of MTHFD1 could pose a potential safety risk and is therefore undesirable due to its high expression in normal tissues. To this end, the development of selective inhibitors of MTHFD2 could constitute an important anticancer drug discovery program, presenting an alternative route to the drug resistance of folate antimetabolites. To the best of our knowledge, tricyclic coumarin-based compounds identified by Kawai et al.^12,13^ and xanthine-based derivatives developed by Lee et al.^14^ are the only selective inhibitors of MTHFD2 reported to date. As evident from the crystallographic studies and kinetic experiments, tricyclic coumarin-based compounds bind to the substrate binding site competitively, whereas xanthine-based inhibitors occupy the allosteric site of MTHFD2.

The first isozyme selective inhibitor (tetrahydropyrido[4,3-*d*]pyrimidin-4-one derivative), which is a substrate site binder, was discovered in a high throughput screening (HTS) campaign, using a thermal shift assay. However, the HTS hit (compound **1**, Figure 2A, PDB code: 6JID) was moderately potent against MTHFD2 with an IC_50_ value of 8.3 μM, but fascinatingly did not show any activity against MTHFD1 isoform (IC_50_ > 100 μM).^12^ Compound **1** was further subjected to rational structure-based drug design, that resulted in the discovery of first lead compound DS44960156 (compound **2**, PDB code: 6JIB), which is a tricyclic coumarin-based derivative with an IC_50_ of 1.6 μM in enzyme assays, exhibiting a high selectivity (>18-fold) for MTHFD2. Replacing the central tetrahydropyrido-pyrimidin-4-one ring (compound **1**) with the tricyclic coumarin-based ring (compound **2**) proved to be a key factor in crucially improving the binding affinities and corresponding IC_50_ values for MTHFD2. In continuation, compound **2** was employed in structure-guided optimization studies which led to a series of highly potent and selective MTHFD2 inhibitors (denoted as “DS18” series).

One of the best compounds from the “DS18” series called compound 18 (here denoted as compound **3** for clarity, cf Figure 2A) possessed IC_50_ of 0.048 μM and 6.4 μM for MTHFD2 and MTHFD1 isoforms, respectively, demonstrated a prominent selectivity profile, and was cocrystallized with MTHFD2 (PDB code: 6KG2). The best compound of the “DS18” series labeled DS18561882 (compound **4**), showed 0.0063 μM and 0.57 μM IC_50_ values for MTHFD2 and MTHFD1, respectively. Compound **4** was identified as highly potent, selective, and orally available MTHFD2 inhibitor, exhibiting >250-fold greater potency than the parent compound **2**. Furthermore, compound **4** illustrated a good pharmacokinetic profile and high cell-based activity of 0.14 μM GI_50_ against the MDA-MB-231 cell line derived from human breast cancer.^13^

In a recent study, Bonagas et al.^14^ reported the discovery of new diaminopyrimidine-based MTHFD2 inhibitors (TH7299, TH9028, and TH9619, Figure 2A), which showed IC_50_ values in the nanomolar range. The three diaminopyrimidine-based inhibitors were identified in a high throughput screening (HTS) assay of over 500,000 lead-like compounds, followed by structure-guided lead optimization.^14^ TH7299, TH9028 and TH9619 were cocrystallized with MTHFD2 (PDB codes: 6S4E, 6S4A, and 6S4F, respectively), occupy the substrate binding site of MTHFD2 and exhibited IC_50_ values of 254 nM, 11 nM, and 47 nM, respectively, in biochemical assays. Furthermore, MTHFD2 inhibitors were found to reduce the replication fork speed and result in replication stress followed by S-phase arrest and apoptosis of acute myeloid leukemia cells *in vitro* and *in vivo*. The aforementioned MTHFD2 inhibitors prevented thymidine production, causing misincorporation of uracil into DNA and replication stress. Despite the striking outcomes, the three compounds show inhibition of MTHFD1 and MTHFD2L (another isoform expressed in adult tissues^15^ and embryonic cells^16^), indicating nonselective MTHFD2 inhibition. TH7299, TH9028, and TH9619 showed IC_50_ values of 89 nM, 0.5 nM, and 16 nM, respectively, against MTHFD1. Similarly, TH7299, TH9028, and TH9619 were found to inhibit MTHFD2L in a biochemical screening with 126 nM, 27 nM, and 147 nM IC_50_ values, respectively.^14^ Moreover, the selectivity issues with these inhibitors were also addressed by the same research group,^17^ pinpointing the challenges associated with the development of selective MTHFD2 inhibitors that will show no or poor binding on MTHFD1 and MTHFD2L isoforms.

Lee et al.^18^ presented the discovery of xanthine derivatives as selective MTHFD2 inhibitors. Interestingly, the xanthine derivatives were found to occupy an allosteric site of MTHFD2 instead of the substrate binding site, consequently impeding the binding of the cofactor and phosphate to MTHFD2. Four X-ray crystal structures were solved for MTFHD2, three of which contained a selective allosteric inhibitor; compound **6** (PDB code: 7EHM), compound **7** (PDB code: 7EHV), and compound **8** (PDB code: 7EHN), which all coexisted with a folate-based inhibitor (compound **9**, resembling tetrahydrofolate), confirming that the binding of xanthine derivatives took place at the allosteric site, whereas compound **9** was accommodated into the substrate binding site (Figure 2B). The fourth X-ray crystal structure of MTHFD2 in this series was characterized by the absence of any allosteric inhibitor, however composed of the compound **9** in complex with cofactor NAD^+^ and pyrophosphate (P_i_) (PDB code: 7EHJ). The comparison and superposition of the above-mentioned X-ray crystal structures revealed that MTHFD2 underwent several conformational changes upon binding to allosteric inhibitors, that subsequently obstructed the binding of the cofactor and phosphate to MTHFD2. Kinetic studies on MTHFD2 inhibition by compound **6**, compound **8**, and compound **9** were furthermore performed in order to elucidate the mechanism of the enzymatic inhibition. The degree of MTHFD2 inhibitory activity at any fixed concentration of folate-based inhibitor reduced on increasing the substrate concentration (tetrahydrofolate), which highlights a competitive mode of MTHFD2 inhibition by compound **9**. On the contrary, xanthine derivatives (compound **6** and compound **8**) showed a completely different response in comparison to the folate-based inhibitor. The degree of MTHFD2 inhibitory activity instead increased with increasing substrate concentration at a given concentration of compound **6** and compound **8**, indicating a noncompetitive mode of MTHFD2 inhibition. This noncompetitive mode of inhibition by xanthine-based inhibitors correlates with the above-mentioned X-ray structures, specifying the formation of catalytically inactive ternary complexes (enzyme–substrate-inhibitor), thus confirming a novel allosteric binding mode.^18^

In the present study, we first gathered information on the availability of X-ray crystal structures, existence of reported/cocrystallized inhibitors that include both the substrate site binders and allosteric binders (as briefly discussed above), and compared the MTHFD2 binding sites (both substrate and allosteric) with the MTHFD1 binding site to get deeper insights toward selectivity inclination. In particular, we extensively focused on the substrate binding site of MTHFD2 and analyzed the cocrystallized X-ray structures with the aid of molecular dynamics (MD) simulations. A detailed investigation was carried out on the crystallographic binding mode, dynamic movement, and corresponding protein–ligand interactions of the potent and selective MTHFD2 inhibitors (particularly tricyclic coumarin-based compounds) that demonstrated promising selectivity and highly potent biological activity. Furthermore, utilizing the information from MD analyses and exploiting the crystallographic binding mode of one of the most important tricyclic coumarin-based inhibitors, we executed a structure-based drug design campaign involving R-group enumeration, bioisosteric replacement, molecular docking, ADME prediction, MM-GBSA analysis, and MD simulations. At the end of this study, a small library of new, selective, and potent inhibitors of MTHFD2 are proposed, that are derived from the tricyclic coumarin-based scaffold. Comparative docking, binding free energy calculations, and MD simulations of existing and proposed MTHFD2 inhibitors were also carried out on the MTHFD1 binding site, to validate the selectivity.

## 2 Results and Discussion

### Comparison of the MTHFD1 and MTHFD2 Binding Sites for Designing Selective MTHFD2 Inhibitors

**Figure 3:**
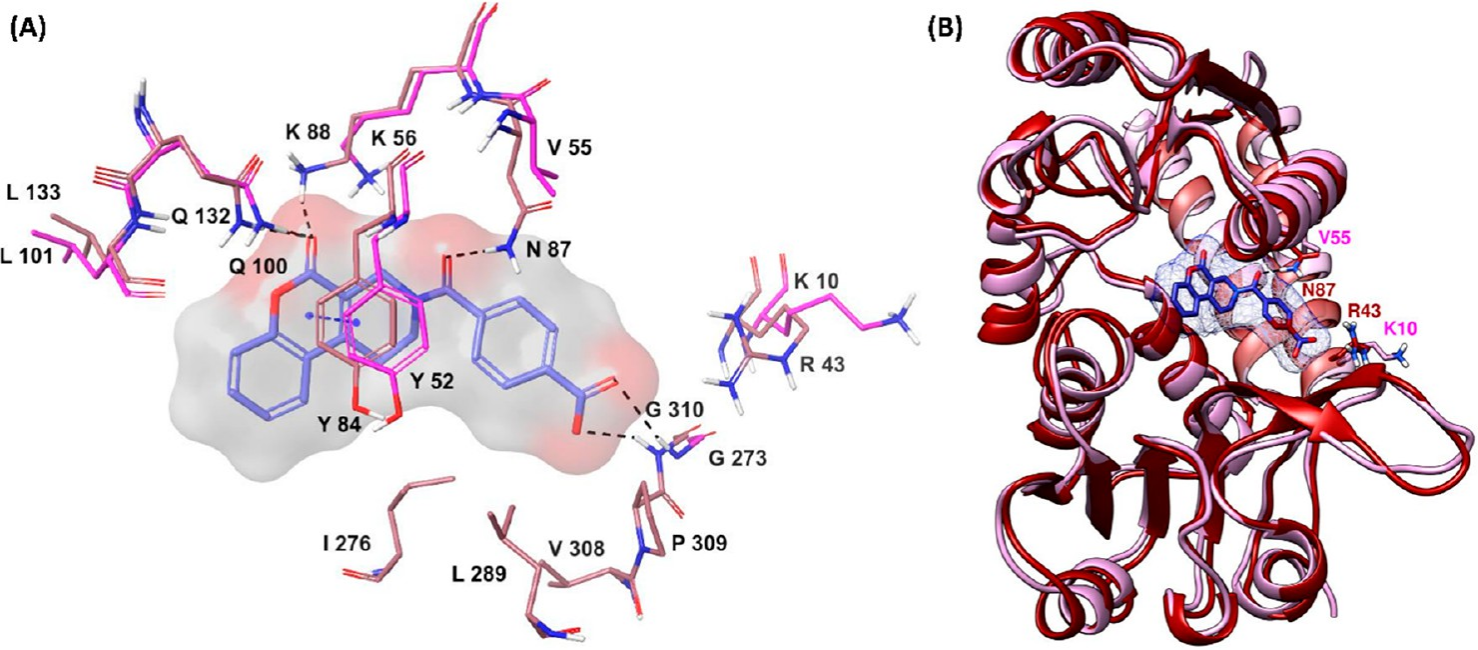
(A) Compound **2** (blue) in the substrate binding site of MTHFD2 (brown residues, PDB code: 6JIB), superposed with MTHFD1 (pink residues, PDB code: 6ECQ). (B) MTHFD2-MTHFD1 superposed structures (ribbon view). The MTHFD2 selectivity residues Arg43 and Asn87 (in brown) that are replaced by Lys10 and Val55 (in pink) in MTHFD1 are shown in the stick model.

As mentioned above, MTHFD2 is absent in healthy human tissues while exclusively expressed in a variety of cancer cells such as breast cancer and colorectal cancer.^8−10^ This makes MTHFD2 a promising therapeutic target for anticancer drug discovery programs, with the possibility of minimal side effects. On the other hand, MTHFD1, a homologous protein of MTHFD2 sharing 36.36% sequence identity and 53.64% sequence similarity with the MTHFD2 isoform is widely expressed in healthy human tissues.^19,20^ MTHFD2 inhibitors discovered in the past, such as LY345899^7,10^ (compound **5**) and carolacton,^11^ showed notable anticancer activities in the biological assays but were found to concurrently inhibit MTHFD1, thus presenting a potential safety risk. In this context, the discovery of selective MTHFD2 inhibitors is of great importance, that will broaden the therapeutic window and overcome the toxicity and side effects associated with targeting healthy tissues. For deeper insight at the binding site level, the X-ray crystal structures of MTHFD1 and MTHFD2 isoforms were superposed, taking into account the substrate binding site as well as the allosteric site. The X-ray crystal structure of MTHFD1 in complex with compound **5** and NADP (PDB code: 6ECQ)^21^ was used for comparison with the MTHFD2 X-ray crystal structures. As shown in Figure 3, the MTHFD2-compound **2** complex (PDB code: 6JIB)^12^ was superposed with the MTHFD1-compound **5** complex (PDB code: 6ECQ)^21^ (for clarity, only a few residues of MTHFD1 that have relevance to the substrate binding site are shown, while other residues and cocrystallized compound **5** of MTHFD1 are hidden). Compound **2** forms H-bond interactions with the side chains of Asn87, Lys88, and Gln132 and with the backbone nitrogen of Gly310 and a π–π stacking with Tyr84. These protein–ligand interactions are regarded as essential components, that contribute to the binding affinity for the target. Four out of six residues are identical in the substrate binding site of MTHFD1; Lys56, Gln100, Gly273, and Tyr52, while Val55 and Lys10 are different. The hydrophilic Asn87 in MTHFD2 is replaced by hydrophobic Val55 in MTHFD1, which is placed slightly away from the substrate binding site. The H-bond contact between the carbonyl of the amide linker in compound **3** and Asn87 of MTHFD2 proved to be a pivotal factor in facilitating selectivity. Furthermore, Arg43 in MTHFD2 is replaced by Lys10 in MTHFD1, which is another structural element crucial for selectivity. Lys10 is projected toward the solvent-exposed region, away from substrate binding site, whereas Arg43 is oriented adjacent to Gly310 within the binding site. Despite that compound **2** did not constitute any H-bond contact with Arg43 in the crystal structure, MD simulations (discussed later) reveal that Arg43 significantly contributes in extending the H-bond network at the substrate binding site. Thus, the presence of Arg43 and Asn87 in MTHFD2 (replaced by Lys10 and Val55 in MTHFD1, respectively), provide novel ligands additional H-bond interactions for selective MTHFD2 inhibition. In the current work, we exclusively aimed at the structural investigation of substrate site binders (compounds **1**–**4**) by molecular dynamics simulations, followed by structure-based drug design with reference to tricyclic coumarin-based compound **2**, comparative MM-GBSA analysis, and MD simulations of all compounds bound to the MTHFD1 isozyme, to analyze selective binding.

### Binding Mode of Cocrystallized Compounds **1**–**3** and Docking Pose of Compound **4**

**Figure 4:**
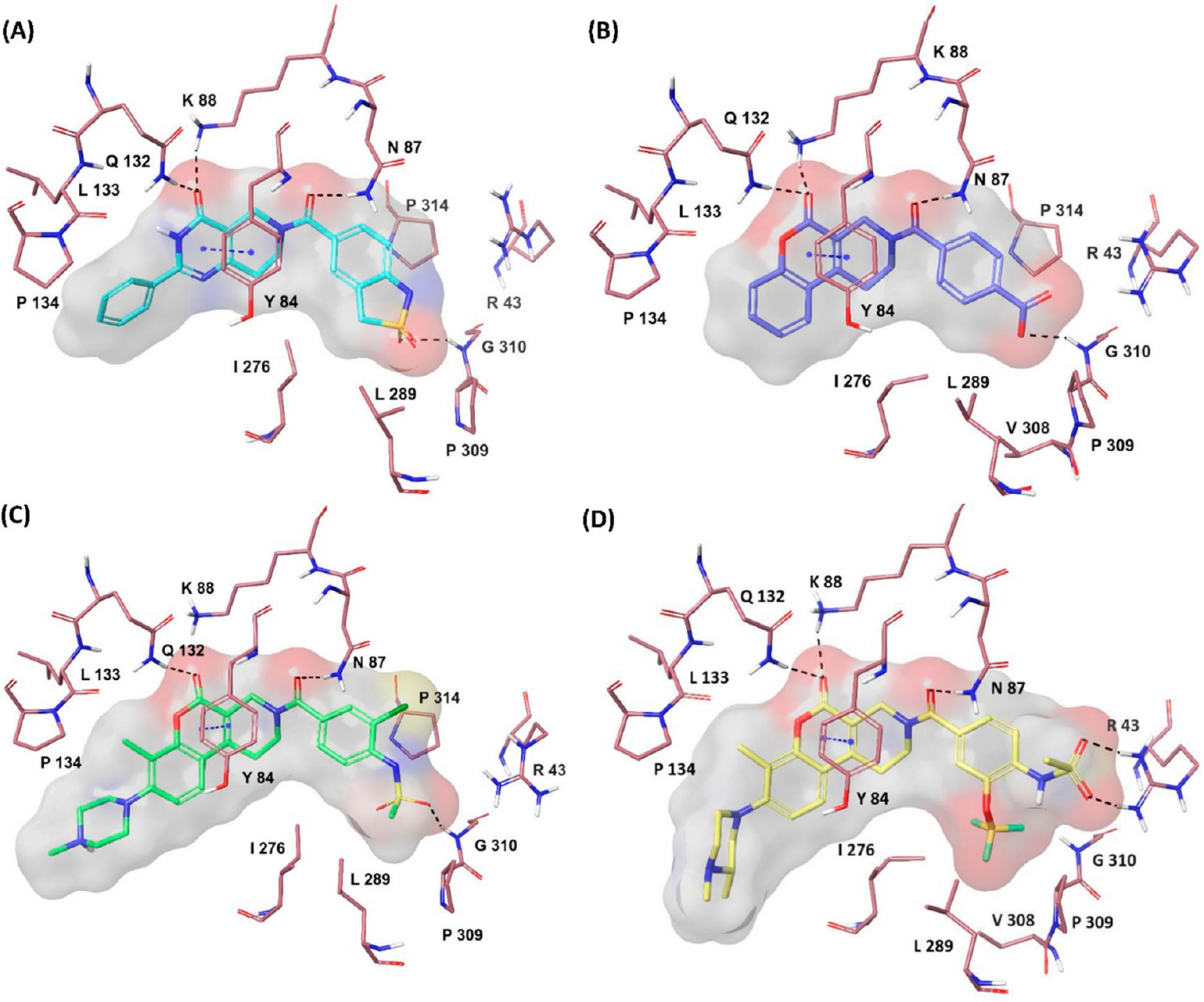
Binding modes of (A) compound **1**, cyan; (B) compound **2**, blue; (C) compound **3**, light green; (D) docking pose of compound **4**, yellow, within the substrate binding site of MTHFD2.

Compound **1** constitutes 4 H-bonds in the binding site of MTHFD2: (a) the carbonyl of the pyrimdine-4-one forms H-bonds with the side chains of Lys88 and Gln132; (b) the carbonyl of the amide linker is H-bonded to the side chain of Asn87; (c) and the sulfonyl of the benzothiazole establishes an H-bond with the backbone nitrogen of Gly310. In addition, the pyrimidine-4-one of compound **1** forms π–π stacking with Tyr84 (Figure 4A). Compound **2** was discovered as a tricyclic coumarin-based inhibitor,^12^ from the structure-guided optimization studies of pyridopyrimidine-based compound **1**, as discussed earlier (Figure 4B). The protein–ligand interactions and the corresponding binding affinity were significantly strengthened due to the modification of the central pyridopyrimidine scaffold to the tricyclic coumarin ring, improving the biological activity. The central tricyclic coumarin ring of compound **2** establishes π–π stacking with Tyr84, similar to the tetrahydropyrido-pyrimidin-4-one ring of compound **1**. Moreover, compound **2** shows maintenance of H-bond interactions with Asn87, Lys88, Gln132, and Gly310, displaying a binding mode similar to compound **1**. Compound **3** was proven to be the best compound among the cocrystallized inhibitors of MTHFD2.^13^ Similar to compounds **1** and **2**, the binding mode of compound **3** demonstrates the existence of essential protein–ligand interactions in the substrate binding site of MTHFD2. The piperazine ring, attached to the central tricyclic coumarin ring of compound **3**, is the new structural element which enhances the solubility and the pharmacokinetic profile^13^ (Figure 4C).

Compound **4** was identified as the most potent and highly selective inhibitor of the “DS” series with an IC_50_ of 0.0063 μM for MTHFD2. Moreover, compound **4** features a strong pharmacokinetic profile and highly potent cell-based activity of 0.14 μM against the MDA-MB-231 cell line derived from human breast cancer.^13^ However, no crystallographic data or hypothesized docking pose is reported for compound **4**. Thus, we identified and analyzed the plausible binding mode of compound **4** in the binding site of MTHFD2 by means of molecular docking and MD simulations. As anticipated from the docking pose, compound **4** retains all essential protein–ligand contacts in the MTHFD2 binding site (Figure 4D). The coumarin carbonyl of compound **4** forms H-bond contacts with the side chains of Lys88 and Gln132, while the tricyclic coumarin ring constitutes π–π stacking with Tyr84. The carbonyl of the amide linker connecting the tricyclic coumarin ring and the N-(2-(trifluoromethoxy) phenyl)methanesulfonamide moiety establishes an H-bond with the Asn87 side chain, which contributes to selectivity. Furthermore, the oxygen atoms of the sulfonamide group of compound **4** forms H-bond interactions with the side chain of Arg43, which is another element potentiating MTHFD2 selectivity. The terminal 1–2, dimethyl piperazine ring of compound **4** contributes to the solubility and the other pharmacokinetic profile. Notably, compound **4** is characterized by the presence of a 2-trifluoromethoxy group from the phenylmethanesulfomamide ring, with the other compounds (compounds **1**–**3**) remaining unsubstituted at this position. The trifluoromethoxy group of compound **4** is well-accommodated into the lipophilic cavity of MTHFD2, surrounded by the residues Ile276, Leu289, Val308, and Pro309, providing van der Waals contacts and contributing to the binding affinity.

### MD Simulations of Compounds **1**–**4**

In order to investigate the conformational dynamics, the cocrystallized substrate binders compounds **1**–**3** and the docked inhibitor (compound **4**) were subjected to 200 ns MD simulations, starting from their crystallographic/docking poses. We calculated the Root Mean Square Deviation (RMSD) of the ligand heavy atoms, as a measure of ligand mobility. The RMSD depicts the deviation of the atoms from their initial crystallographic/hypothesized binding pose, during the simulation trajectory. Likewise, we calculated the RMSD of the α-carbons of the protein as a measure of protein mobility, throughout the simulation.

**Figure 5:**
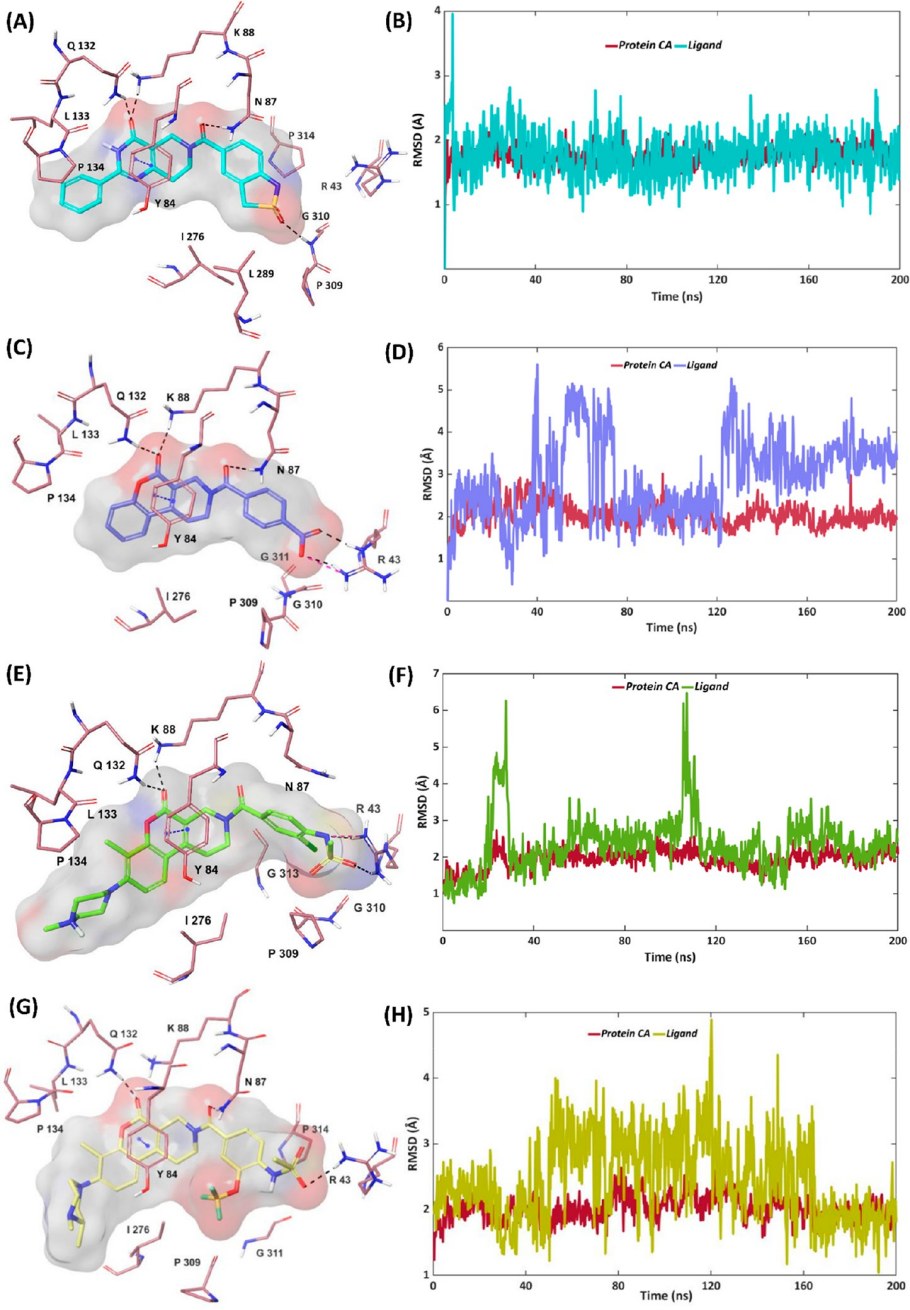
(A) Representative MD structure of MTHFD2-compound **1** complex (inhibitor in cyan, protein residues in brown, PDB code: 6JID). (B) RMSD analysis of MTHFD2-compound **1** complex (ligand in cyan, protein α-carbons in brown) during the 200 ns simulation. (C) Representative MD structure of MTHFD2-compound **2** complex (inhibitor in blue, protein residues in brown, PDB code: 6JIB). (D) RMSD analysis of MTHFD2-compound **2** complex (ligand in blue, protein α-carbons in brown). (E) Representative MD structure of MTHFD2-compound **3** complex (inhibitor in light green, protein residues in brown, PDB code: 6KG2). (F) RMSD analysis of MTHFD2-compound **3** complex (ligand in light green, protein α-carbons in brown). (G) Representative MD structure of MTHFD2-compound **4** complex (inhibitor in yellow, protein residues in brown). (H) RMSD analysis of MTHFD2-compound **4** complex (ligand in yellow, protein α-carbons in brown).

As evident from the RMSD plot (Figure 5B), compound **1** exhibits great stability in the MTHFD2 binding site, maintaining an average RMSD of 1.8 Å over the course of the simulation. Furthermore, the above-mentioned protein–ligand interactions exist predominantly during the simulation trajectory; π–π stacking between compound **1** and Tyr84 is present throughout the simulation, while the H-bond interactions between compound **1** and Asn87, Lys88, Gln132, and Gly310 were maintained for 86%, 88%, 98%, and 97% of the simulation time, respectively (Figures 5A, S1). Compound **2** shows some degrees of fluctuations during the first 125 ns of the trajectory until convergence is attained between the α-carbons of the protein and the inhibitor. A new H-bond interaction between the inhibitor and the Arg43 side chain is observed, which exists for the whole simulation time (Figure 5C,D). Arg43 is one of the important residues in the substrate binding site, which has the potential to influence the selectivity for MTHFD2, as it is replaced by a solvent-exposed Lys10 in MTHFD1. Furthermore, both Arg43 and Gly310, which are located adjacent to each other, can be exploited by potential ligands to extend their H-bond network in the substrate binding site. The presence of the tricyclic coumarin ring and the benzoic acid in compound **2** act as key anchoring points to reproduce the interaction profile as shown by compound **1** (i.e., with Tyr84, Asn87, Lys88, and Gln132) (Figure S2). Thus, despite some RMSD fluctuations, the substantial improvement in biological activity and selectivity of compound **2** is corroborated by occurrence of essential protein–ligand interactions, as well as the expansion of the H-bond network to Arg43. The dynamic stability of compound **3** seems to be better than compound **2**, with a few minor fluctuations in the simulation trajectory (Figure 5E,F). Compound **3** retains π–π stacking with Tyr84 for the whole simulation time, while the H-bond contacts with the side chains of Arg43, Asn87, Lys88, and Gln132 account for 79%, 44%, 37%, and 81% of the simulation trajectory, respectively (Figure S3). Identical to compound **2**, the H-bond/ionic interaction between compound **3** and Arg43 plays a pivotal role in facilitating highly potent biological activity and remarkable selectivity for MTHFD2. The said interaction between compound **1** and Arg43 was found to be negligible over the course of simulation, reflecting the moderate potency and relatively poor selectivity of compound **1**. Furthermore, the presence of the central pyridopyrimidine system in compound **1**, instead of the tricyclic coumarin scaffold, is believed to cause the moderate MTHFD2 inhibition thereof.

Conformational dynamics of compound **4** indicate a fluctuation in the RMSD plot, between 49 and 164 ns of the 200 ns simulation trajectory (Figure 5G,H). In particular, two different conformations of the terminal 1–2, dimethyl piperazine were noted during the simulation, one of which contributes to the increase in RMSD values. Figure S4 illustrates MD snapshots of compound **4** at 36th ns and 164th ns of the simulation, respectively. The 1,2-dimethyl piperazine moiety of compound **4** is located at the solvent-exposed region at 36th ns, corresponding to lower RMSD values, whereas at 164th ns, the 1,2-dimethyl piperazine system is projected toward Leu289, facilitating van der Waals contacts with the *N*-methyl group of the piperazine unit, however, thus yielding a fluctuation in the RMSD. This movement of the piperazine ring system shifts the OCF_3_ of compound **4** away from Leu289, however, closer to Ile276. Compound **4** forms lipophilic contacts with Tyr84 and H-bond contacts with Gln132 during 100% of the simulation time, while H-bond interactions between compound **4** and Asn87 and Lys88 account for 46% and 38% of the simulation time, respectively. As discussed above, the 2-trifluoromethyl group of compound **4** is suggested to crucially contribute in improving the binding affinity. This is verified by the MD analysis, through van der Waals contacts between compound **4** and Ile276 during 35% of the trajectory (Figure S5).

Based on the information gathered from the MD analyses of the cocrystallized inhibitors (compounds **1**–**3**) and the published inhibitor (compound **4**), we hypothesize that apart from the essential protein–ligand interactions, the H-bond contacts with the unique residues Arg43 and Asn87 substantially contribute to the binding affinity and selectivity for MTHFD2. The dynamic instabilities noted for compounds **2**–**4** can be anticipated also for the other existing tricyclic coumarin-based inhibitors as well as newly designed inhibitors based on the same scaffold. Similar to compound **4**, the lipophilic pocket in the substrate binding site of MTHFD2 can be exploited for designing potential inhibitors with enhanced binding affinity. Taking all these observations into account, we thus performed structure-based design of new tricyclic coumarin-based inhibitors, discussed below.

### Structure-Based Design of New Tricyclic Coumarin-Based Compounds

**Figure 6:**
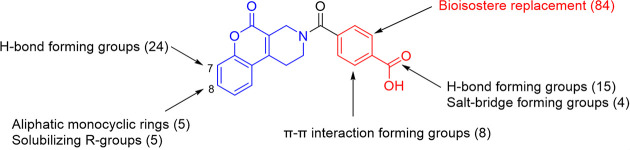
Structure-based design of new tricyclic coumarin derivatives with reference to compound **2**.

Based on experimental evidence and computational modeling of compounds **2**–**4**, the tricyclic coumarin scaffold appears to be an integral structural element facilitating desirable binding modes and providing essential protein–ligand interactions at the substrate binding site of MTHFD2. Therefore, we kept this tricyclic coumarin scaffold unaltered and performed structure-based modifications/enumerations on the remaining portions of the structure. Figure 6 shows the structure-based design approach we adopted and performed R-group enumeration at different positions of compound **2** using diverse chemical libraries: aliphatic monocyclic rings, H-bond forming groups, salt-bridge forming groups, and solubilizing R-groups. In addition, the benzoic acid moiety of compound **2**, attached to the tricyclic coumarin scaffold by an amide linker, was subjected to bioisosteric replacement. Targeting the carboxylate of benzoic acid, 15 new compounds with H-bond forming groups and 4 new compounds with salt-bridge forming groups were identified to reproduce/extend the H-bond network with Arg43 and Gly310. Furthermore, we targeted the lipophilic cavity in the MTHFD2 binding site and performed R-group enumeration at the second position of the benzoic acid ring in compound **2** (i.e., 2-OCF_3_ in compound **4**) using π–π interaction forming groups, which increased our library with 8 new compounds. The seventh position of the tricyclic coumarin scaffold was subjected to R-group enumeration with H-bond forming groups, aimed at offering additional H-bond contacts to Gln132 and Leu133, which led to 24 new compounds, Finally, with reference to compounds **3** and **4**, that are characterized by the presence of piperazine and 1–2, dimethyl piperazine respectively, the eighth position of the tricyclic coumarin scaffold was exploited for R-group enumeration using libraries of aliphatic monocyclic rings and solubilizing R-groups, with the purpose of improving aqueous solubility and pharmacokinetic parameters. This enumeration resulted in 10 new compounds: 5 derived from the aliphatic monocylic rings and 5 derived from the solubilizing R-groups. Counting each group of structures individually (outcomes of R-group enumeration and bioisosteric replacement) from the structure-based design approach, 145 compounds in total were developed as potential inhibitors of MTHFD2 (Figure 6).

Since selectivity is a major concern for MTHFD1/2 inhibition, the 145 designed compounds (Figure S28, Table S2) were docked into the substrate binding sites of both MTHFD2 and MTHFD1. 94 compounds were filtered based on satisfying the following two cutoff criteria: (a) docking score of ≤ −7.5 kcal/mol in the MTHFD2 binding site to anticipate desirable binding and retaining essential protein–ligand interactions and (b) docking score ≥ −6.5 kcal/mol in the MTHFD1 binding site, to ensure poor and undesirable binding.

#### ADME Properties

Poor ADME features such as absorption and extensive first-pass metabolism lead to the failure of a majority of drug candidates in preclinical stages of the drug development process. Through an early ADME screening, failure of potential drug candidates in the drug discovery programs has been reportedly reduced. Thus, accurate data on ADME properties not only helps in selecting good drug candidates but also provides crucial information for dosage form design and formulation. With this aim, the relevant descriptors of physicochemical and pharmacokinetic properties such as molecular weight, Lipinski Rule of Five (RO5), logP_o/w_, logS, PSA, logBB, CNS activity, log*K*_p_, logHERG, log*K*_hsa_, and percentage human oral absorption (HOA) were selected, and computed for the 94 selected compounds and compared with the existing tricyclic coumarin-based inhibitors (compounds **1**–**4**).^22,23^ 18 potential inhibitors of MTHFD2 (compounds **10**–**27**) were prioritized out of 94 compounds, based on possessing numerical values in the acceptable range (Figure S27, Table S1). First, all compounds were found to obey the Lipinski Rule of 5 and possess molecular weight <500 with the exception of compound **10** with the molecular weight of 556.461 which violates this, although it is still considered tolerable. The octanol/water partition coefficient (logP_o/w_) of all 18 compounds range between 0.6 and 3.7, which is within the recommended values. The aqueous solubility (logS) lies between −6.3 and −2.5 mol/L, suggesting that all molecules are ideal. The polar solvent accessible area (PSA) denotes the ability of a compound to interact with the solvent by dipolar or H-bond interaction. All 18 inhibitors span between 87 to 154 Å of PSA, falling within the acceptable limit. The brain/blood partition coefficient (logBB) of all compounds ranges from −2.6 to −0.5, which is again within the tolerable limit. Furthermore, 16 out of 18 molecules are indicated to have no CNS activity by possessing the value −2.0, while the remaining two analogues were predicted for medium CNS activity with value 1.0, which is within the acceptable limit. The skin permeability parameter (log*K*_p_) is used to predict the penetration of compounds through the skin; all molecules possess values between −5.6 to −2.7, which is inside the ideal range. The prediction of blockage of the HERG K+ channel (logHERG), which represents evaluation of cardiac toxicity of drug molecules, was also computed. All 18 compounds vary between −6.5 and −3.5, falling within the tolerable threshold of logHERG. Finally, other ADME parameters such as prediction of binding to serum albumin, also known as plasma protein binding (log*K*_hsa_), and percentage human oral absorption (HOA), are considered important for drug likeness. The potential 18 compounds possess log*K*_hsa_ between −0.8 and 0.6 and %HOA between 45% and 95%, again all within the acceptable range. In addition, the ADME properties of the reference tricyclic-coumarin based compounds **1**–**4** were also computed and compared with the 18 potential inhibitors. Only compounds **3**–**4** were found to violate the Lipinski Rule of 5 (similar to the proposed compound **10**) possessing molecular weight of 545.052 and 608.631, respectively, whereas all other above-mentioned ADME features lie within the acceptable and recommended values for compounds **1**–**4**, similar or close to the proposed analogues. With all these results taken together, the 18 potential inhibitors of MTHFD2 seem to possess acceptable and promising pharmacokinetic profiles, with the comparative study with the reference compounds **1**–**4** providing an important validation of their drug likeness potential. However, we emphasize that these pharmacokinetics predictions should be subject to experimental evaluations in order to confirm the reliability of the QikProp^22,24^ tool used in this study. The selected 18 compounds were thus subjected to further postdocking refinement by MD simulations.

#### MD Simulations of the Proposed Compounds

**Figure 7:**
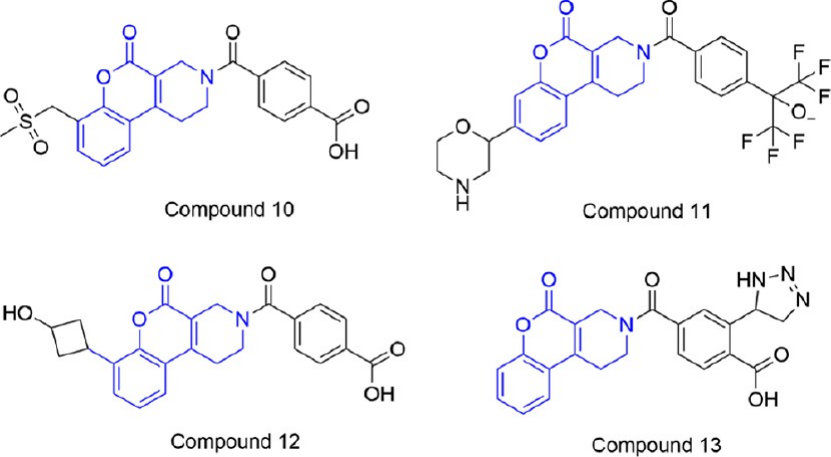
Potential MTHFD2 inhibitors, resulting from structure-based drug design.

**Figure 8:**
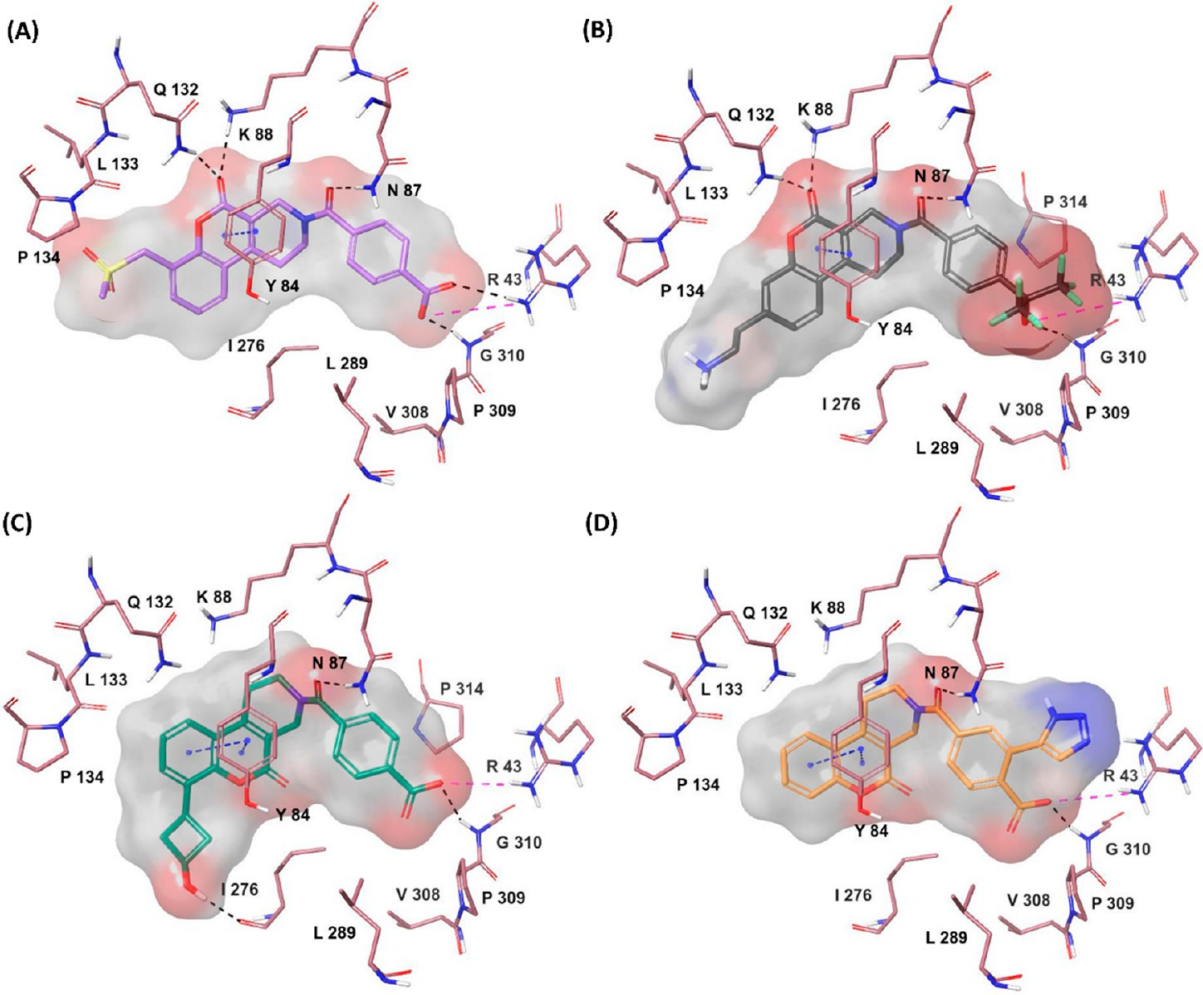
Docking poses of (A) compound **10**, purple; (B) compound **11**, gray; (C) compound **12**, dark green; (D) compound **13**, orange, within the substrate binding site of MTHFD2.

We performed 200 ns MD simulations for the selected 18 compounds, starting from their putative binding poses as suggested by the docking experiment. Conformational dynamics and stability of the key protein–ligand interactions in the MTHFD2 binding site were thoroughly investigated for the selected 18 compounds. Furthermore, the set of compounds was fine-tuned to the best 4 potential inhibitors (compounds **10**–**13**, Figure 7), based on meeting the following two criteria: (a) despite some anticipated RMSD fluctuations, exhibiting a reasonable stability during the course of the simulation, relative to the simulation trajectories of existing tricyclic coumarin-derived inhibitors (compounds **2**–**4**), and (b) forming interactions with at least three out of the five essential residues Arg43, Asn87, Lys88, Gln132, and Gly310 for at least ∼50% of the simulation time. As apparent from the docking poses (Figure 8), compounds **10** and **11** form interactions with Arg43, Tyr84, Asn87, Lys88, Gln132, and Gly310, whereas compounds **12** and **13** are characterized by the absence of H-bond contact with Lys88/Gln132 in the substrate binding site of MTHFD2. Interestingly, a new H-bond interaction is observed between the cyclobutanol (−OH) terminal of compound **12** and Ile276.

**Figure 9:**
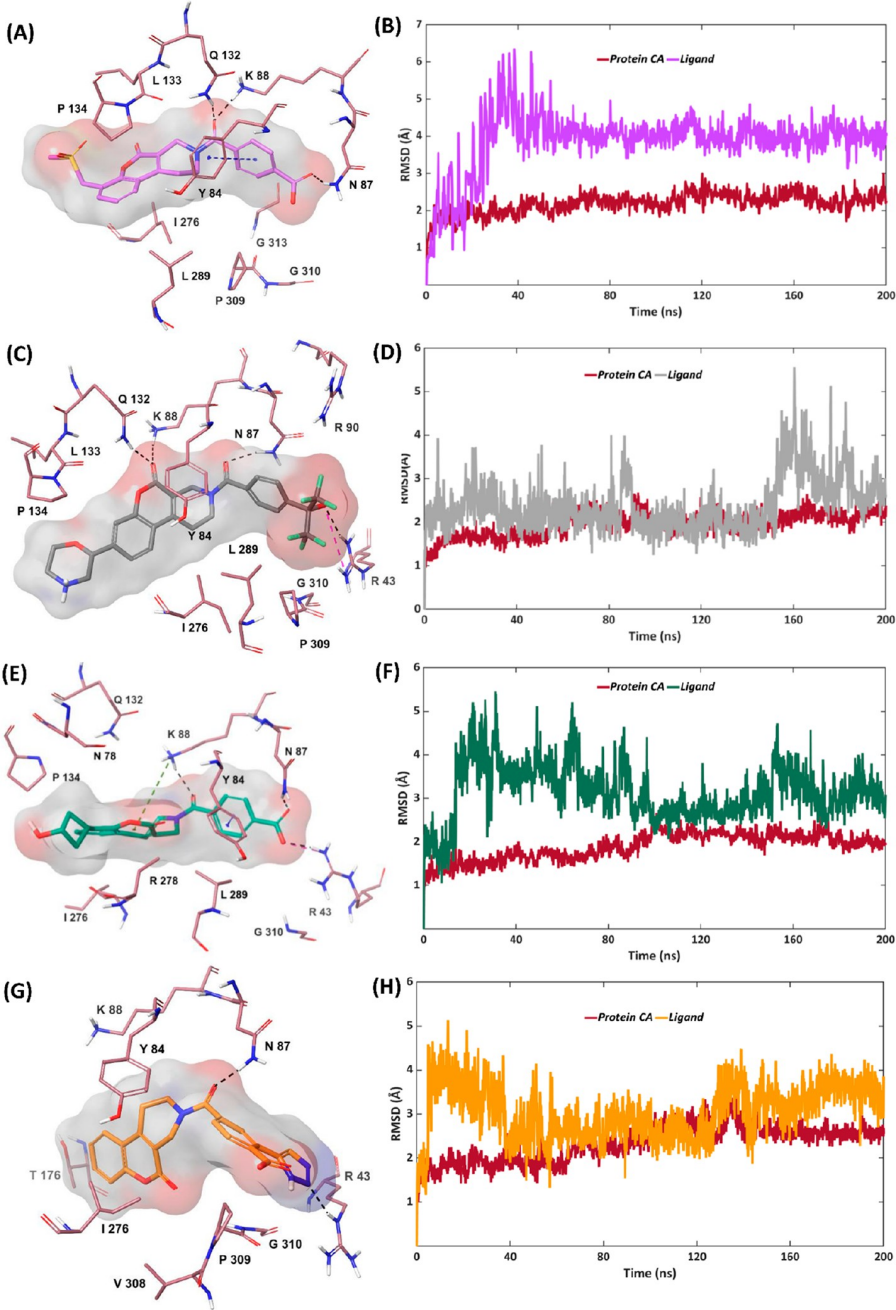
(A) Representative MD structure of MTHFD2-compound **10** complex (inhibitor in purple, protein residues in brown, PDB code: 6JID). (B) RMSD analysis of MTHFD2-compound **10** complex (ligand in purple, protein α-carbons in brown) during the 200 ns simulation. (C) Representative MD structure of MTHFD2-compound **11** complex (inhibitor in gray, protein residues in brown. (D) RMSD analysis of MTHFD2-compound **11** complex (ligand in gray, protein α-carbons in brown). (E) Representative MD structure of MTHFD2-compound **12** complex (inhibitor in dark green, protein residues in brown). (F) RMSD analysis of MTHFD2-compound **12** complex (ligand in dark green, protein α-carbons in brown). (G) Representative MD structure of MTHFD2-compound **13** complex (inhibitor in orange, protein residues in brown). (H) RMSD analysis of MTHFD2-compound **13** complex (ligand in orange, protein α-carbons in brown).

Relative to the trajectories of compounds **2**–**4**, the proposed compound **10** demonstrates a low degree of RMSD fluctuation (between 0 and 50 ns) and then remains stable over the course of simulation (Figure 9A,B). Compound **10** establishes 4 H-bond interactions in the MTHFD2 binding site; (a) between the carbonyl of the amide linker and the side chains of Lys88, Gln132 for ∼55% and ∼80% of the simulation time, respectively; (b) between the terminal carboxylate and the Arg43 side chain for ∼80% of the trajectory; (c) the dynamic mobility of compound **10** leads to a H-bond contact between the terminal carboxylate and the Asn87 side chain, that accounts for ∼70% of the time. Furthermore, the lipophilic contact between the tricyclic coumarin ring system and Tyr84 was maintained abundantly during the course of simulation (Figure S14). Compound **11** features a relatively stable RMSD trend over the course of simulation (Figure 9C,D). Identical to compound **10**, π–π stacking between the central tricyclic coumarin ring of compound **11** and Tyr84 accounted for 100% of the trajectory. H-bond contacts between the carbonyl linker – Asn87 side chain, coumarin carbonyl – Lys88 side chain, and coumarin carbonyl – Gln132 side chain were maintained for ∼50%, ∼60%, and ∼80% of the simulation time, respectively (Figure S15). Unlike compounds **10** and **11**, compound **12** displays RMSD fluctuations during the first 100 ns of the simulation (Figure 9E,F); however, this trend was anticipated for the tricyclic coumarin-based compounds, as discussed earlier. Strikingly, as a result of the dynamic mobility, the H-bond interaction between the terminal carboxylate of compound **12** and the Asn87 side chain was maintained for the whole simulation time, which is hypothesized to potentiate MTHFD2 selectivity. The Lys88 side chain is involved in interactions with the coumarin carbonyl of compound **12** via an H-bond and to the central coumarin ring via π–cation interaction, which altogether accounts for >50% of the simulation trajectory. π–π stacking between the tricyclic coumarin system of compound **12** and Tyr84 was maintained for ∼70% of the simulation time (Figure S16). The proposed compounds **10** and **12** (Figure 7) share a great structural similarity with the cocrystallized compound **2** (Figure 2A); however, compound **2** is characterized by the absence of any substitution at the seventh or eighth position of the tricyclic coumarin ring system, while compound **10** and compound **12** have solvent-exposed extensions of methyl sulfonylmethyl and cyclobutanol groups, respectively, at the seventh position of the central tricyclic coumarin system, improving dynamic stability and binding toward MTHFD2. On comparing the protein–ligand interaction profiles and RMSD fluctuations, both compounds **10** and **12** seem to be consistent and notably better than compound **2**. Compound **2** possesses RMSD fluctuations until 125 ns of the simulation time, forming interactions with Arg43 (100%), Tyr84 (98%), Asn87 (40%), Lys88 (40%), and Gln132 (40%) whereas both compounds **10** and **12** demonstrate improved ligand stability (possibly due to the solvent-exposed substituents at the seventh position) as well as maintaining key interactions similar to compound **2**: Arg43 (80%), Asn87 (70%), Tyr84 (100%), Lys88 (55%), and Gln132 (80%) for compound **10**, and Asn87 (100%), Tyr84 (100%), and Lys88 (50%) for compound **12**, respectively. In particular, the strong interactions of compounds **10** and **12** with the selectivity residue Asn87 can be considered as another integral element facilitating promising and comparable binding to MTHFD2.

The last proposed inhibitor of the series, compound **13** shows a notable RMSD stability over the course of simulation (Figure 9G,H). The central tricyclic coumarin ring of compound **13** retains π–π stacking with Tyr84 during 50% of the simulation. Though the H-bond/salt-bridge contacts with both Arg43 (carbonyl of amide linker) and Asn87 (azetidine nitrogen) are each present for only 30% of the simulation, taken together (60% of the simulation trajectory), compound **13** seems to exhibit promising selectivity for MTHFD2. Interestingly, a new lipophilic interaction between the tricyclic coumarin ring system and Ile276 was established, existing for ∼50% of the trajectory, and is believed to additionally contribute to the binding affinity (Figure S17). Based on the observations from the suggested binding poses, promising docking scores on MTHFD2 and poor docking scores on MTHFD1 (Table 1), evaluation of the ligand mobility, and interaction stability by MD simulations, we infer compounds **10**–**13** as potential inhibitors of MTHFD2, that are predicted to exhibit anticancer activities.

**Table 1:** Glide Scores of All Compounds in the MTHFD2 and MTHFD1 Binding Site

Entry	MTHFD2 (−kcal/mol)	MTHFD1 (−kcal/mol)
Compound **1**	–8.8	–7.8
Compound **2**	–8.8	–5.5
Compound **3**	–7.9	–5.4
Compound **4**	–8.2	–6.7
Compound **10**	–9.0	–5.3
Compound **11**	–8.8	–6.1
Compound **12**	–9.0	–5.8
Compound **13**	–9.4	–6.5

### MM-GBSA Binding Free Energy Results

We carried out binding free energy evaluations using MM-GBSA^25^ on the proposed inhibitors (compounds **10**–**13**) to authenticate the binding potential for MTHFD1/MTHFD2, and to compare with the binding energies of the existing tricyclic coumarin-derived inhibitors (compounds **1**–**4**). The cocrystallized inhibitor of MTHFD1, which is a folate analogue (LY345899, compound **5**, PDB code: 6ECQ(21)), was used as a control to assess the poor binding of compounds **10**–**13** on MTHFD1. As depicted in Table 2, a significant difference in the binding free energies (Δ*G*_Bind_) was observed for all compounds across the two binding sites. For instance, compound **2**, which is the first tricyclic coumarin-derived inhibitor, possessed a high −83.60 kcal/mol binding free energy toward MTHFD2, while it demonstrates a critical reduction in Δ*G* Bind (to −40.89 kcal/mol) for MTHFD1. In addition, a crucial difference in the Coulomb energy contribution (Δ*G* Coulomb) was observed for compound **2** between MTHFD2 (−72.76 kcal/mol) and MTHFD1 (22.16 kcal/mol). The experimental binding affinities of compound **2** (1.6 μM IC_50_, >18-fold selectivity) are thus in agreement with the binding free energy results. Likewise, the experimental binding affinities of the other cocrystallized/published inhibitors, compound **1** (8.3 μM IC_50_, > 12-fold selectivity), compound **3** (0.048 μM IC_50_, > 133-fold selectivity), and compound **4** (0.0063 μM IC_50_, > 250-fold selectivity) illustrate a good correlation with the calculated binding free energies in the MTHFD2 and MTHFD1 binding sites. In the same framework, the herein proposed inhibitors (compounds **10**–**13**) show significantly higher Δ*G* Bind (from −76.20 to −70.23 kcal/mol) and Δ*G* Coulomb (from −88.32 to −31.42 kcal/mol) for MTHFD2, as compared to MTHFD1 (−56.84 to −36.56 kcal/mol for Δ*G* Bind and 8.70 to 26.08 for Δ*G* Coulomb). In addition, MM-GBSA calculations were also performed on the cocrystallized pose of the folate analogue (compound **5**) in the MTHFD1 binding site, giving −62.98 kcal/mol for Δ*G* Bind and 34.41 kcal/mol for Δ*G* Coulomb. In relation to this, the proposed inhibitors (compounds **10**–**13**) hence illustrate poor binding energetics toward MTHFD1, which authenticate their potential to selectively inhibit MTHFD2.

**Table 2:** MM-GBSA Binding Free Energy Results (Δ*G* Bind and Δ*G* Coulomb in kcal/mol) of the Docked/Cocrystallized Structures in MTHFD2 and MTHFD1

Entry	Δ*G* Bind (MTHFD2)	Δ*G* Bind (MTHFD1)	Δ*G* Coulomb (MTHFD2)	Δ*G* Coulomb (MTHFD1)
Compound **1**	–79.95	–55.09	–72.76	22.16
Compound **2**	–83.60	–40.89	–77.45	23.05
Compound **3**	–88.64	–57.74	–33.64	43.71
Compound **4**	–85.00	–61.08	–28.2	–4.93
Compound **5**	—-	–62.98	—-	34.14
Compound **10**	–76.20	–36.56	–78.39	26.08
Compound **11**	–75.57	–55.49	–31.42	13.24
Compound **12**	–75.94	–56.84	–88.32	8.70
Compound **13**	–70.23	–51.07	–81.84	17.72

### MD Simulations of MTHFD2 Inhibitors Bound to MTHFD1

**Figure 10:**
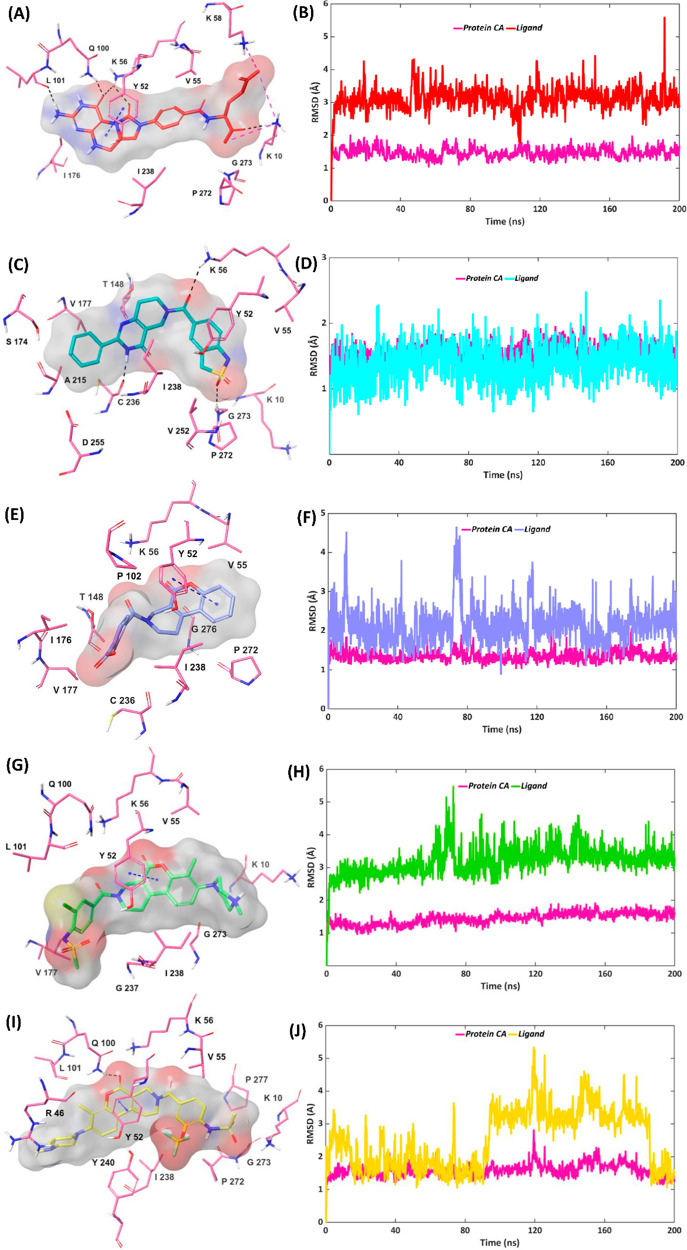
(A) Representative MD structure of MTHFD1-compound **5** complex (inhibitor in red, protein residues in pink, PDB code: 6ECQ). (B) RMSD analysis of MTHFD1-compound **5** complex (ligand in red, protein α-carbons in pink) during the 200 ns simulation. (C) Representative MD structure of MTHFD1-compound **1** complex (inhibitor in cyan, protein residues in pink). (D) RMSD analysis of MTHFD1-compound **1** complex (ligand in cyan, protein α-carbons in pink). (E) Representative MD structure of MTHFD1-compound **2** complex (inhibitor in blue, protein residues in pink). (F) RMSD analysis of MTHFD1-compound **2** complex (ligand in blue, protein α-carbons in pink). (G) Representative MD structure of MTHFD1-compound **3** complex (inhibitor in light green, protein residues in pink). (H) RMSD analysis of MTHFD1-compound **3** complex (ligand in light green, protein α-carbons in pink). (I) Representative MD structure of MTHFD1-compound **4** complex (inhibitor in yellow, protein residues in pink). (J) RMSD analysis of MTHFD1-compound **4** complex (ligand in yellow, protein α-carbons in pink).

In relation to the crystallographic binding mode of compound **5** (LY345899, folate analogue, PDB code: 6ECQ), undesirable binding or improper accommodation of compounds **1**–**4** and compounds **10**–**13** is anticipated in the MTHFD1 binding site, to facilitate selectivity for MTHFD2. Docking results reveal that compounds **1**–**4** and compounds **10**–**13** adopt binding modes dissimilar to the cocrystallized pose of compound **5** (Figures S6–S13), with only partial accommodation into the MTHFD1 binding site. However, some protein–ligand interactions were noted, that were further investigated through MD simulations. The docking scores of compounds **1**–**4** and compounds **10**–**13** are all less than in MTHFD2, thus in agreement with the observed poor binding toward MTHFD1. Before investigating the conformational dynamics of these compounds, we performed MD simulations on the MTHFD1–compound **5** cocrystallized complex. Compound **5** was found to be consistently stable throughout the simulation trajectory, starting from the initial crystallographic pose, as observed in the RMSD plot (Figure 10A,B). Compound **5** is characterized by the presence of central pteridine-imidazole nucleus, attached to glutamic acid by a phenyl ring. The carbonyl of the imidazolidin-2-one unit forms one H-bond with the side chain of Lys56 (Lys88 in MTHFD2) throughout the simulation time and another H-bond with the side chain of Gln100 (Gln132 in MTHFD2) during 95% of the trajectory. Due to the long and bulky glutamic acid of compound **5** being able to protrude into the MTHFD1 binding site, its carboxy side chain terminal engages in a H-bond interaction with the Lys58 side chain (Arg90 in MTHFD2) for ∼60% of the time, whereas the carboxy backbone of the glutamic acid forms an H-bond with the backbone nitrogen of Gly273 (Gly310 in MTHFD2) during a small fraction of the simulation. The crystallographic binding mode depicts π–π stacking between the phenyl of compound **5** and Tyr52; however, the MD analysis reveals that π–π stacking favorably occurs with the pyrazine of the compound **5** pteridine unit, for 77% of the simulation trajectory. Other notable interactions of compound **5** include an H-bond between the amine of pteridine and the backbone oxygen of Leu101 for 50% of the simulation time and lipophilic contact between the phenyl ring of compound **5** and the Val55 side chain (selectivity present in MTHFD1) for 41% of the trajectory (Figure S18). The dynamic stability and the existence of protein–ligand interactions of compound **5** could serve as a reference for comparing MD results of compounds **1**–**4** and compounds **10**–**13** in MTHFD1.

The suggested binding mode of compound **1** from the docking studies (Figure S6) shows only one interaction in the MTHFD1 binding site: between the sulfonyl part of the benzothiazole unit and the backbone nitrogen of Gly273, which is present during 78% of the simulation. Furthermore, MD studies revealed the presence of a few additional and unusual interactions between compound **1** and MTHFD1. An H-bond between the pyrimidine nitrogen of compound **1** and the backbone oxygen of Cys236 was observed throughout the simulation, that was not seen in either the crystallographic pose or during the MD simulation of compound **5**. The phenyl ring of the benzothiazole group of compound **1** forms π–π stacking with Tyr52 for 53%, while the pyrido-pyrimidine ring establishes lipophilic contact with the Ile238 side chain for 68% of the simulation time. The carbonyl of the amide linker connecting the pyrido-pyrimidine and benzothiazole forms a H-bond with the side chain of Lys56 during 42% of the simulation. Despite a significant RMSD stability (Figure 10C,D), no interactions were observed between compound **1** and the selectivity residues Lys10 (Arg43 in MTHFD2) and Val55 (Asn87 in MTHFD2), nor with Gln100 and Leu101, which explains to the poor MTHFD1 inhibition with an IC_50_ > 100 μM (Figure S19). Only two interactions were noted from the suggested docking pose of compound **2** in the MTHFD1 binding site: (a) a H-bond between the carbonyl of the amide linker and the Gln100 side chain (34%) and (b) a H-bond between the carbonyl of the tricyclic coumarin ring and the Lys56 side chain, during 28% of the simulation trajectory. The phenyl ring and the central tricyclic coumarin system of compound **2** contribute to π–π interaction with Tyr52 for the entire simulation (Figure S20).

Compound **2** displays minor fluctuations over the course of simulation (Figure 10E,F); however, the peculiar binding mode and the absence of essential protein–ligand interactions are assumed to result in the poor MTHFD1 binding affinity, while on the contrary, facilitating >18-fold selectivity for MTHFD2. As suggested from the docking pose, compound **3** exerts a flipped binding mode and only partial occupancy of the MTHFD1 binding site, further characterized by the following two interactions: (a) a H-bond between the carbonyl of the tricyclic coumarin ring and the Gln100 side chain, (b) a H-bond between the coumarin oxygen and the Lys56 side chain. π–π stacking between the pyrone of the tricyclic coumarin scaffold and Tyr52 was the only major interaction, present during 90% of the simulation time (Figure S21). Compound **3** shows a stable RMSD trend over the course of simulation (Figure 10G,H); however, the flipped binding mode and the absence of essential protein–ligand interactions explains >133-fold selectivity for MTHFD2. Compound **4**, which was identified as the most potent and highly selective MTHFD2 inhibitor, exhibits only one interaction in the MTHFD1 binding site. As hypothesized from the docking pose, compound **4** forms π–π stacking with Tyr52, that accounts for 73% of the simulation time. From the MD analysis, compound **4** furthermore establishes H-bond contact with the Gln100 side chain during 45% of the trajectory, while interactions with other important residues Lys56, Lys58, Leu101, Val55, and Gly273 were either negligible or absent over the course of the simulation (Figure S22). Taken together, these reflect the >250-fold selectivity for MTHFD2. Interestingly, the RMSD plot of compound **4** depicts some degrees of ligand fluctuation between ∼90–180 ns (Figure 10I,J), which were found to be similar to the hallmark demonstrated by the tricyclic coumarin-based inhibitors in the MTHFD2 binding site (compounds **2**–**4**).

**Figure 11:**
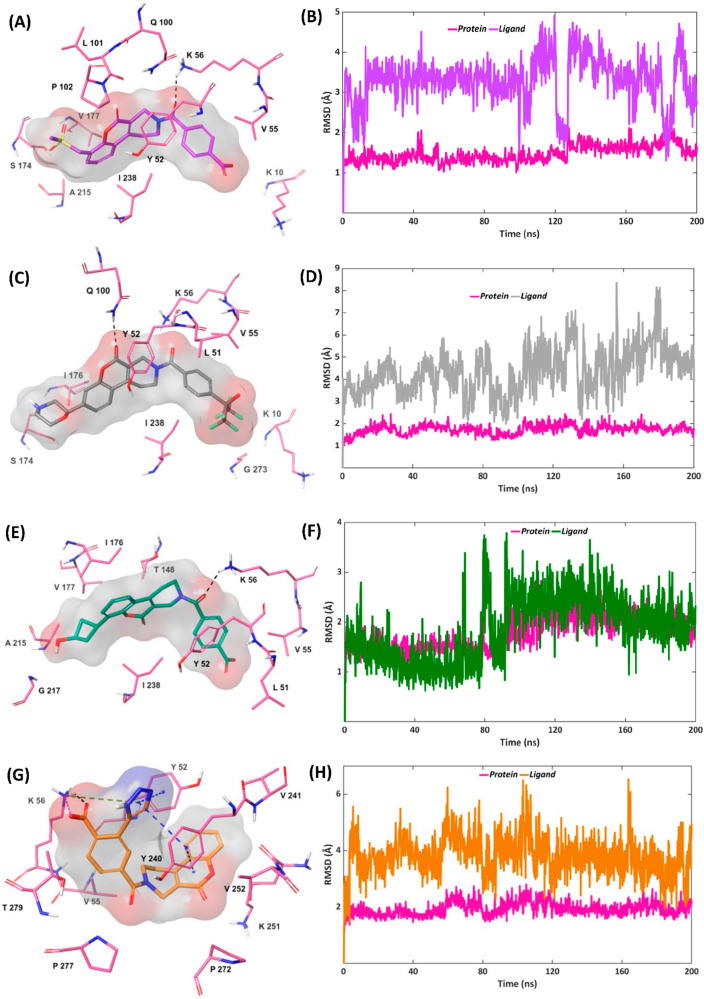
(A) Representative MD structure of MTHFD1-compound **10** complex (inhibitor in purple, protein residues in pink). (B) RMSD analysis of MTHFD1-compound **10** complex (ligand in purple, protein α-carbons in pink) during the 200 ns simulation. (C) Representative MD structure of MTHFD1-compound **11** complex (inhibitor in gray, protein residues in pink). (D) RMSD analysis of MTHFD1-compound **11** complex (ligand in gray, protein α-carbons in pink). (E) Representative MD structure of MTHFD1-compound **12** complex (inhibitor in dark green, protein residues in pink). (F) RMSD analysis of MTHFD2-compound **12** complex (ligand in dark green, protein α-carbons in pink). (G) Representative MD structure of MTHFD1-compound **13** complex (inhibitor in orange, protein residues in pink). (H) RMSD analysis of MTHFD1-compound **13** complex (ligand in orange, protein α-carbons in pink).

The first proposed inhibitor of MTHFD2 from our structure-based design campaign, compound **10**, shows the existence of only one main interaction in the MTHFD1 binding site from the putative docking pose. The carboxylate of compound **10** forms an H-bond contact with the Lys56 side chain, which is maintained for ∼70% of the simulation time. MD simulations also show an additional interaction, π–π stacking between the tricyclic coumarin ring and Tyr52 for ∼70% of the time (Figure S23), while interactions with other important residues were either negligible or missing over the course of simulation. Furthermore, relatively high RMSD with notable fluctuations was observed for compound **10** during the simulation (Figure 11A,B). Similar to compound **10**, the hypothesized binding pose of compound **11** is characterized by the presence of only one interaction at the MTHFD1 binding site, an H-bond between the oxyanion of compound **11** and the backbone nitrogen of Gly273. This was however completely absent during the course of the simulation. Instead, a couple of new interactions were noted from the MD analysis: (a) π–π stacking between the tricyclic coumarin ring and Tyr52, for 99% of the simulation; (b) an H-bond between the coumarin carbonyl and the Gln100 side chain, for 48% of the trajectory (Figure S24). A high degree of ligand fluctuation is again observed in the RMSD plot (Figure 11C,D) during the simulation, which clearly shows a prominent and desirable instability of compound **11** in the MTHFD1 binding site. Compound **12** features notable dynamic stability and convergence with the α-carbons of MTHFD1 over the course of the simulation (Figure 11E,F). Despite that, the accommodation of compound **12** into the substrate binding site of MTHFD1 is peculiar and only partially agrees with that of the cocrystallized compound **5**. The suggested docking pose of compound **12** depicts only two interactions with MTHFD1: (a) an H-bond between the carbonyl of the amide linker (connecting the tricyclic coumarin ring and the benzoic acid) and the Gln100 side chain, found to be absent during the simulation trajectory (the same group, however, forms a H-bond with the Lys56 side chain during >50% of the simulation); (b) π–π stacking between the tricyclic coumarin ring and Tyr52, present for >60% of the simulation. An unusual lipophilic contact between the tricyclic coumarin ring of compound **12** and Ile238 is noted from the MD analysis, that is accounted for during >40% of the simulation (Figure S25). Finally, for compound **13**, the docking experiment predicts an atypical binding pose and incomplete occupancy of the compound in the MTHFD1 binding site. The RMSD plot indicates fairly large fluctuations of the ligand over the course of the simulation (Figure 11G,H). The putative binding pose of compound **13** features only one interaction; a H-bond/salt bridge formation between the carboxylate and the Lys56 side chain. MD analysis confirms that the said interaction is accounted for during 97% of the simulation trajectory. In addition, the dynamic movement of both the triazole and the phenyl ring in compound **13** leads to π–π stacking with Tyr52 and Tyr240, that are maintained abundantly throughout the simulation trajectory (Figure S26).

Comparative MD analyses of the proposed MTHFD2 inhibitors (compounds **10**–**13**) with the existing tricyclic coumarin analogues (compounds **1**–**4**) and with the cocrystallized folate-based inhibitor (compound **5**, LY345899), substantiate their poor binding and emphasize the potential to selectively inhibit MTHFD2. In general, compounds **10**–**13** were in part found to occupy the MTHFD1 binding site in a peculiar manner, that correlate with RMSD fluctuations and the absence of essential protein–ligand interactions. Despite the reasonable ligand stability and existence of a few strong protein–ligand interactions in some cases (e.g., the high RMSD stability of compound **12** and strong lipophilic contacts seen for compound **13**), none of the proposed compounds have shown any H-bond interaction with Lys10 (Arg43 in MTHFD2) or any lipophilic contact with Val55 (Asn87 in MTHFD2), that have critical contribution toward MTHFD2 selectivity. Thus, based on the observations from the putative docking poses and MD simulations, we hypothesize that compounds **10**–**13** can be considered as potential and selective inhibitors of MTHFD2.

## 4 Experimental Section

### Protein Preparation

The X-ray crystal structures of MTHFD2 in complex with compound **1** (PDB code: 6JID), compound **2** (PDB code: 6JIB), and compound **3** (PDB code: 6KG2), MTHFD1 in complex with compound **5** (PDB code: 6ECQ), and MTHFD2 in complex with the allosteric inhibitor (compound **6**, PDB code: 7EHM), were downloaded from the protein data bank.^26^ All cocrystallized structures were prepared using protein preparation wizard,^27,28^ as implemented in Maestro, Schrödinger.^29^ Hydrogen atoms were added, and the possible metal binding states were generated. The Prime module^30^ of Schrödinger was used to add missing atoms, side chains, and loops^27,31,32^ to the X-ray complexes (if required), followed by assigning protonation states and generation of tautomeric states for Asp, Glu, Arg, Lys, and His at pH 7.0 ± 2.0. H-bond refinement of all protein structures was done using the PROPKA module^33^ of Schrödinger^29^ at pH 7.0, and water molecules with fewer than two hydrogen bonds to nonwaters were removed. Finally, geometry refinements of all protein–ligand complexes were done using the OPLS4 force field^34−37^ in restrained minimization, to fix molecular overlaps and strains. The restrained minimization was completed as soon as the average root-mean-square deviation (RMSD) of the protein heavy atoms had converged to 0.3 Å.

### Ligand Preparation

The cocrystallized inhibitors (compounds **1**–**3**, **5**), the published inhibitor (compound **4**), and the proposed inhibitors (compounds **10**–**13**) were prepared using the LigPrep module^38^ of the Schrödinger suite.^29^ Epik,^39−41^ a tool for p*K*_a_ prediction, was used to assign possible ionization and tautomeric states at pH 7.0 ± 2.0. Energy minimization of the ligands were carried out using OPLS4 force field.^34^

### Molecular Docking

Docking experiments were performed by using Glide^42−45^ employing the prepared ligand structures as discussed above. The prepared protein complex structures of MTHFD2-compound **2** (PDB code: 6JIB) and MTHFD1-compound **5** (PDB code: 6ECQ) were used for generating receptor grids for docking into the MTHFD2 and MTHFD1 binding sites, respectively. Cubic receptor grids were generated with respect to the centroid of the bound ligand in the substrate binding site, with a side length of 20 Å. No constraints were applied to any of the receptor grids. Docking was carried out under default settings, using the standard precision (SP) mode, allowing flexible ligand sampling also involving nitrogen inversions and ring conformations of the ligands. The default parameters of the 0.8 scaling factor for van der Waals radii of nonpolar ligand atoms and 0.15 partial charge cutoff were used. Postdocking minimization was performed, and a maximum of 100 poses per ligand was retained during docking. The default Glide scoring function was used to select the top-ranked docking pose for each ligand. OPLS4 force field^34^ was used during the docking procedure. The reliability of the docking experiment was verified by performing redocking/self-docking of the cocrystallized ligands, compound **2** and compound **5** on MTHFD2 and MTHFD1 binding sites, respectively. In both cases, the Glide SP docking mode was able to reproduce the crystallographic poses, with marginal RMSD differences.

### ADME Prediction and Drug Likeness of Potential MTHFD2 Inhibitors

Estimation of ADME properties and the prediction of pharmacokinetic and physicochemical parameters reportedly boost the selection of drug candidates in preclinical trials, accounting for the “drugability” of a molecule. The QikProp module^22−24^ in Schrödinger^29^ was used to calculate ADME parameters of the tricyclic coumarin-based inhibitors (compounds **1**–**4**) as well as the initial 94 compounds resulting from the structure-based design workflow. This set was reduced to 18 compounds, based on required values to lie within the recommended ranges. The following pharmacokinetic and physicochemical properties were computed in this study: molecular weight, Lipinski Rule of Five (RO5), octanol/water partition coefficient (logP_o/w_), aqueous solubility (logS), polar solvent accessible area (PSA), blood/brain partition coefficient (logBB), CNS activity, skin permeability parameter (log*K*_p_), blockage of HERG K+ channel (logHERG), prediction of binding to serum albumin/plasma protein binding (log*K*_hsa_), and percentage human oral absorption (HOA).

### MD Simulations and Clustering

Starting from the crystallographic pose or the putative docking pose of the existing/proposed inhibitors in MTHFD2 (PDB code: 6JIB) and MTHFD1 (PDB code: 6ECQ) binding sites, classical MD simulations were performed using the Desmond program^46,47^ in Schrödinger suite 2022–1.^29^ Each protein–ligand complex was solvated in TIP3P water^48^ using an orthorhombic box with periodic boundary conditions, placed in such a way that the walls were at a minimum 10 Å distance from any atom in the system. Na^+^ and Cl^–^ ions were added to neutralize the overall charge of each system, as appropriate. A salt concentration of 0.15 M NaCl was added to the simulation box to reproduce physiological conditions. The default Desmond protocol in the NPT ensemble^47^ was used for minimization and relaxation of each system. The OPLS4 force field^34^ was applied during all simulations. Each simulation was run for a total of 200 ns with a recording interval of 200 ps. Employing the Nose-Hoover thermostat and Martyna–Tobias–Klein barostat with isotropic coupling,^49−51^ the temperature and pressure of the system were kept constant at 300 K and 1.01325 bar atmospheric pressure, respectively. Using the simulation interaction diagram (SID) panel as implemented in Schrödinger, the simulation trajectories were analyzed for root-mean-square deviations (RMSD), protein–ligand interactions and root-mean-square fluctuations (RMSF) of both proteins and ligands. The obtained trajectories were clustered according to RMSD using the “Desmond Trajectory Clustering” module,^47^ setting up a frequency value of 10 (every 10th ns) and up to a maximum of 10 clusters. With regard to the MD simulations of cocrystallized complexes, the obtained cluster resembling the crystallographic pose was used as a representative structure, while with respect to the MD simulations of docked complexes, the most populated cluster was used as a representative structure.

### Binding Free Energy Calculation

Molecular mechanics with generalized Born and surface area solvation (MM-GBSA) is a widely used method to estimate the binding free energy of a ligand, bound to a protein.^25^ The Prime module^30^ in Schrödinger suite 2022–1^29^ was employed to compute MM-GBSA free energy of binding (Δ*G* Bind) for the crystallographic and docking poses, with respect to MTHFD2 and MTHFD1, using the following equation:
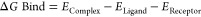
where *E*_Complex_, *E*_Ligand_, and *E*_Receptor_ represent the energies calculated in the Prime MM GBSA module for the optimized complex (complex), optimized free ligand (ligand), and optimized free receptor (receptor), respectively. The OPLS4 force field^34^ and VSGB solvation model^52^ were used in the calculations, featuring minimization of protein–ligand complexes as the sampling method. The binding site residues (protein residues within 5 Å from each ligand) were treated as flexible. The binding free energies (Δ*G* Bind) and the Coulomb energy contribution (Δ*G* Coulomb) of each ligand were discussed in this work.

## 5 Conclusions

In this study, the structural basis of MTHFD2 inhibition by the existing pyrido-pyrimidine based inhibitor^12^ (compound **1**) and the tricyclic coumarin-based compounds^12,13^ (compounds **2**–**3**) has been elucidated with the aid of computational modeling. The cocrystallized poses of these inhibitors were further analyzed by MD simulations, with the purpose of interrogating ligand conformational flexibility and the stability of essential protein–ligand interactions in the substrate binding site of MTHFD2, that contribute to the overall binding affinity. In addition, the binding mode and interactions of the most potent and highly selective MTHFD2 inhibitor discovered from the “DS” series of tricyclic coumarin-based inhibitors^13^ (compound **4**) were predicted by molecular docking followed by MD analysis, the results of which were found to be well correlated with the experimental data. Observations from the crystallographic/docking poses and MD analyses of the existing MTHFD2 inhibitors outlined essential protein–ligand interactions (with Arg43, Tyr84, Asn87, Lys88, Gln132, and Gly310), that are required for MTHFD2 inhibition. In particular, the H-bond interactions with Arg43 and Asn87, that are substituted by Lys10 and Val55 in MTHFD1, are of critical importance to facilitate selectivity for MTHFD2, taking into account the fact that MTHFD1 is exclusively present in human healthy tissues while MTHFD2 is found to be overexpressed in certain cancer types such as breast and colorectal cancer.^10,19,20^ Furthermore, docking/MD results of compound **4** displayed additional interactions in the lipophilic cavity of the MTHFD2 binding site (2-OCF_3_ of compound **4** with Ile276, Leu289, Val308, and Pro309), that crucially enhanced the MTHFD2 inhibitory activity and selectivity. The lipophilic cavity along with the essential protein residues could be targeted to develop promising MTHFD2 inhibitors. In order to validate the selective MTHFD2 inhibition, the existing inhibitors were also docked into the MTHFD1 binding site, followed by MM-GBSA binding free energy calculations and MD simulations. These highlighted their poor binding to MTHFD1, thus in good agreement with experimental data. With regard to the structural insights on the existing inhibitors binding to MTHFD2, we infer that the tricyclic coumarin ring system is a promising scaffold in facilitating essential/additional protein–ligand contacts and desirable binding modes at the MTHFD2 binding site. We thus implemented a structure-based drug design campaign with reference to compound **2**, keeping the tricyclic coumarin system unaltered while making modifications at the other portions. The structure-based design workflow involved R-group enumeration, bioisostere replacement, molecular docking, ADME prediction, MM-GBSA binding free energy calculations, and MD simulations, to identify new and potential hit compounds. Notably, in order to identify selective molecules, the structure-based design strategy involved refinement of hit compounds based on possessing high docking scores on MTHFD2 and poor docking scores on MTHFD1, as a preliminary filter. The pharmacokinetic predictions, MM-GBSA calculations, and MD simulations enabled us to identify 4 new compounds as potential MTHFD2 inhibitors. To validate poor/undesirable binding on MTHFD1, the proposed inhibitors were tested on the MTHFD1 binding site by implementing a computational protocol (MM-GBSA analysis and MD simulations) identical with that used for the existing MTHFD2 inhibitors, in order to verify selectivity. The proposed inhibitors were not only found to be the good binders to MTHFD2, but demonstrated fluctuating RMSD values, improper accommodation and absence of essential protein–ligand interactions in the MTHFD1 binding site, thus confirming results in accordance with the existing inhibitors and verifying their selectivity for MTHFD2. The findings in this work could provide a better structural understanding of MTHFD2 inhibitors occupying the substrate binding site and simultaneously showing poor binding toward MTHFD1 binding site, justifying their selective MTHFD2 inhibition. We believe that the outcome of our study provides medicinal chemists useful guidelines for rational structure-based design of highly potent and selective MTHFD2 inhibitors for cancer treatment, taking into account the formation of essential protein–ligand interactions and desirable binding modes. Furthermore, the computational methodology employed in this study could be used as a benchmark for new compounds to predict their potency and selective binding toward MTHFD2. Experimental evaluation of the proposed compounds for both MTHFD2 inhibition and pharmacokinetic properties will provide a robust validation to the computational setup employed in this study.

## Data Availability

The structures of the docked complexes (compounds **10**–**13** in MTHFD2 and compounds **1**–**4**, compounds **10**–**13** in the MTHFD1), MD trajectory files (compounds **1**–**4**, compounds **10**–**13** in MTHFD2, and compounds **1**–**5**, compounds **10**–**13** in MTHFD1) are provided as tarballs (.tar.gz) freely accessible at https://zenodo.org/, through DOI: 10.5281/zenodo.7097374. The Root-Mean Square Fluctuation (RMSF) analysis from the 200 ns MD simulations of compounds **1**–**4** and compounds **10**–**13** with respect to MTHFD2 and MTHFD1 is enclosed as “RMSF-Analysis.pdf” in the same Zenodo archive.

## Supporting Information Available

Crystallographic binding poses, docking poses, protein–ligand interaction histograms of compounds **1**–**5** and compounds **10**–**13** with respect to both MTHFD1 and MTHFD2 isoforms, and structures and docking scores of all 145 designed compounds (PDF)

## References

[ref1] Bray F.; Laversanne M.; Weiderpass E.; Soerjomataram I. The Ever-Increasing Importance of Cancer as a Leading Cause of Premature Death Worldwide. Cancer 2021, 127 (16), 3029–3030. 10.1002/cncr.33587. 34086348

[ref2] Sung H.; Ferlay J.; Siegel R. L.; Laversanne M.; Soerjomataram I.; Jemal A.; Bray F. Global Cancer Statistics 2020: GLOBOCAN Estimates of Incidence and Mortality Worldwide for 36 Cancers in 185 Countries. CA. Cancer J. Clin. 2021, 71 (3), 209–249. 10.3322/caac.21660. 33538338

[ref3] Ferlay J.; Colombet M.; Soerjomataram I.; Parkin D. M.; Piñeros M.; Znaor A.; Bray F. Cancer Statistics for the Year 2020: An Overview. Int. J. Cancer 2021, 149 (4), 778–789. 10.1002/ijc.33588. 33818764

[ref4] Ferlay J.; Soerjomataram I.; Dikshit R.; Eser S.; Mathers C.; Rebelo M.; Parkin D. M.; Forman D.; Bray F. Cancer Incidence and Mortality Worldwide: Sources, Methods and Major Patterns in GLOBOCAN 2012. Int. J. Cancer 2015, 136 (5), E359 10.1002/ijc.29210. 25220842

[ref5] Reich S.; Nguyen C. D. L.; Has C.; Steltgens S.; Soni H.; Coman C.; Freyberg M.; Bichler A.; Seifert N.; Conrad D.; Knobbe-Thomsen C. B.; Tews B.; Toedt G.; Ahrends R.; Medenbach J. A Multi-Omics Analysis Reveals the Unfolded Protein Response Regulon and Stress-Induced Resistance to Folate-Based Antimetabolites. Nat. Commun. 2020, 11 (1), 2936 10.1038/s41467-020-16747-y. 32522993 PMC7287054

[ref6] Nilsson R.; Jain M.; Madhusudhan N.; Sheppard N. G.; Strittmatter L.; Kampf C.; Huang J.; Asplund A.; Mootha V. K. Metabolic Enzyme Expression Highlights a Key Role for MTHFD2 and the Mitochondrial Folate Pathway in Cancer. Nat. Commun. 2014, 5 (1), 3128 10.1038/ncomms4128. 24451681 PMC4106362

[ref7] Gustafsson R.; Jemth A.-S.; Gustafsson N. M. S.; Färnegårdh K.; Loseva O.; Wiita E.; Bonagas N.; Dahllund L.; Llona-Minguez S.; Häggblad M.; Henriksson M.; Andersson Y.; Homan E.; Helleday T.; Stenmark P. Crystal Structure of the Emerging Cancer Target MTHFD2 in Complex with a Substrate-Based Inhibitor. Cancer Res. 2017, 77 (4), 937–948. 10.1158/0008-5472.CAN-16-1476. 27899380

[ref8] Liu F.; Liu Y.; He C.; Tao L.; He X.; Song H.; Zhang G. Increased MTHFD2 Expression Is Associated with Poor Prognosis in Breast Cancer. Tumor Biol. 2014, 35 (9), 8685–8690. 10.1007/s13277-014-2111-x. 24870594

[ref9] Ju H.-Q.; Lu Y.-X.; Chen D.-L.; Zuo Z.-X.; Liu Z.-X.; Wu Q.-N.; Mo H.-Y.; Wang Z.-X.; Wang D.-S.; Pu H.-Y.; Zeng Z.-L.; Li B.; Xie D.; Huang P.; Hung M.-C.; Chiao P. J.; Xu R.-H. Modulation of Redox Homeostasis by Inhibition of MTHFD2 in Colorectal Cancer: Mechanisms and Therapeutic Implications. JNCI J. Natl. Cancer Inst. 2019, 111 (6), 584–596. 10.1093/jnci/djy160. 30534944 PMC6579745

[ref10] Tedeschi P. M.; Vazquez A.; Kerrigan J. E.; Bertino J. R. Mitochondrial Methylenetetrahydrofolate Dehydrogenase (MTHFD2) Overexpression Is Associated with Tumor Cell Proliferation and Is a Novel Target for Drug Development. Mol. Cancer Res. 2015, 13 (10), 1361–1366. 10.1158/1541-7786.MCR-15-0117. 26101208 PMC4618031

[ref11] Fu C.; Sikandar A.; Donner J.; Zaburannyi N.; Herrmann J.; Reck M.; Wagner-Döbler I.; Koehnke J.; Müller R. The Natural Product Carolacton Inhibits Folate-Dependent C1Metabolism by Targeting FolD/MTHFD. Nat. Commun. 2017, 8 (1), 1529 10.1038/s41467-017-01671-5. 29142318 PMC5688156

[ref12] Kawai J.; Ota M.; Ohki H.; Toki T.; Suzuki M.; Shimada T.; Matsui S.; Inoue H.; Sugihara C.; Matsuhashi N.; Matsui Y.; Takaishi S.; Nakayama K. Structure-Based Design and Synthesis of an Isozyme-Selective MTHFD2 Inhibitor with a Tricyclic Coumarin Scaffold. ACS Med. Chem. Lett. 2019, 10 (6), 893–898. 10.1021/acsmedchemlett.9b00069. 31223444 PMC6580548

[ref13] Kawai J.; Toki T.; Ota M.; Inoue H.; Takata Y.; Asahi T.; Suzuki M.; Shimada T.; Ono K.; Suzuki K.; Takaishi S.; Ohki H.; Matsui S.; Tsutsumi S.; Hirota Y.; Nakayama K. Discovery of a Potent, Selective, and Orally Available MTHFD2 Inhibitor (DS18561882) with in Vivo Antitumor Activity. J. Med. Chem. 2019, 62 (22), 10204–10220. 10.1021/acs.jmedchem.9b01113. 31638799

[ref14] Bonagas N.; Gustafsson N. M. S.; Henriksson M.; Marttila P.; Gustafsson R.; Wiita E.; Borhade S.; Green A. C.; Vallin K. S. A.; Sarno A.; Svensson R.; Göktürk C.; Pham T.; Jemth A.-S.; Loseva O.; Cookson V.; Kiweler N.; Sandberg L.; Rasti A.; Unterlass J. E.; Haraldsson M.; Andersson Y.; Scaletti E. R.; Bengtsson C.; Paulin C. B. J.; Sanjiv K.; Abdurakhmanov E.; Pudelko L.; Kunz B.; Desroses M.; Iliev P.; Färnegårdh K.; Krämer A.; Garg N.; Michel M.; Häggblad S.; Jarvius M.; Kalderén C.; Jensen A. B.; Almlöf I.; Karsten S.; Zhang S. M.; Häggblad M.; Eriksson A.; Liu J.; Glinghammar B.; Nekhotiaeva N.; Klingegård F.; Koolmeister T.; Martens U.; Llona-Minguez S.; Moulson R.; Nordström H.; Parrow V.; Dahllund L.; Sjöberg B.; Vargas I. L.; Vo D. D.; Wannberg J.; Knapp S.; Krokan H. E.; Arvidsson P. I.; Scobie M.; Meiser J.; Stenmark P.; Berglund U. W.; Homan E. J.; Helleday T. Pharmacological Targeting of MTHFD2 Suppresses Acute Myeloid Leukemia by Inducing Thymidine Depletion and Replication Stress. Nat. Cancer 2022, 3 (2), 156–172. 10.1038/s43018-022-00331-y. 35228749 PMC8885417

[ref15] Bolusani S.; Young B. A.; Cole N. A.; Tibbetts A. S.; Momb J.; Bryant J. D.; Solmonson A.; Appling D. R. Mammalian MTHFD2L Encodes a Mitochondrial Methylenetetrahydrofolate Dehydrogenase Isozyme Expressed in Adult Tissues. J. Biol. Chem. 2011, 286 (7), 5166–5174. 10.1074/jbc.M110.196840. 21163947 PMC3037629

[ref16] Shin M.; Bryant J. D.; Momb J.; Appling D. R. Mitochondrial MTHFD2L Is a Dual Redox Cofactor-Specific Methylenetetrahydrofolate Dehydrogenase/Methenyltetrahydrofolate Cyclohydrolase Expressed in Both Adult and Embryonic Tissues. J. Biol. Chem. 2014, 289 (22), 15507–15517. 10.1074/jbc.M114.555573. 24733394 PMC4140906

[ref17] Scaletti E. R.; Gustafsson Westergren R.; Andersson Y.; Wiita E.; Henriksson M.; Homan E. J.; Jemth A.-S.; Helleday T.; Stenmark P. The First Structure of Human MTHFD2L and Its Implications for the Development of Isoform-Selective Inhibitors. ChemMedChem. 2022, 17 (18), e202200274 10.1002/cmdc.202200274. 35712863 PMC9796130

[ref18] Lee L.-C.; Peng Y.-H.; Chang H.-H.; Hsu T.; Lu C.-T.; Huang C.-H.; Hsueh C.-C.; Kung F.-C.; Kuo C.-C.; Jiaang W.-T.; Wu S.-Y. Xanthine Derivatives Reveal an Allosteric Binding Site in Methylenetetrahydrofolate Dehydrogenase 2 (MTHFD2). J. Med. Chem. 2021, 64 (15), 11288–11301. 10.1021/acs.jmedchem.1c00663. 34337952 PMC8389891

[ref19] Patel H.; Christensen K. E.; Mejia N.; MacKenzie R. E. Mammalian Mitochondrial Methylenetetrahydrofolate Dehydrogenase-Cyclohydrolase Derived from a Trifunctional Methylenetetrahydrofolate Dehydrogenase-Cyclohydrolase-Synthetase. Arch. Biochem. Biophys. 2002, 403 (1), 145–148. 10.1016/S0003-9861(02)00203-5. 12061812

[ref20] Allaire M.; Li Y.; MacKenzie R. E.; Cygler M. The 3-D Structure of a Folate-Dependent Dehydrogenase/Cyclohydrolase Bifunctional Enzyme at 1.5 å Resolution. Structure 1998, 6 (2), 173–182. 10.1016/S0969-2126(98)00019-7. 9519408

[ref21] Bueno R.; Dawson A.; Hunter W. N. An Assessment of Three Human Methylenetetrahydrofolate Dehydrogenase/Cyclohydrolase-Ligand Complexes Following Further Refinement. Acta Crystallogr. Sect. F 2019, 75 (3), 148–152. 10.1107/S2053230X18018083. PMC6404858 30839287

[ref22] Jorgensen W. L.; Duffy E. M. Prediction of Drug Solubility from Structure. Adv. Drug Delivery Rev. 2002, 54 (3), 355–366. 10.1016/S0169-409X(02)00008-X. 11922952

[ref23] Duffy E. M.; Jorgensen W. L. Prediction of Properties from Simulations: Free Energies of Solvation in Hexadecane, Octanol, and Water. J. Am. Chem. Soc. 2000, 122 (12), 2878–2888. 10.1021/ja993663t.

[ref24] Schrödinger Release 2022–1; QikProp, Schrödinger, LLC, New York, NY, 2021.

[ref25] Massova I.; Kollman P. A. Combined Molecular Mechanical and Continuum Solvent Approach (MM-PBSA/GBSA) to Predict Ligand Binding. Perspect. Drug Discovery Des. 2000, 18 (1), 113–135. 10.1023/A:1008763014207.

[ref26] Berman H. M.; Westbrook J.; Feng Z.; Gilliland G.; Bhat T. N.; Weissig H.; Shindyalov I. N.; Bourne P. E. The Protein Data Bank. Nucleic Acids Res. 2000, 28 (1), 235–242. 10.1093/nar/28.1.235. 10592235 PMC102472

[ref27] Madhavi Sastry G.; Adzhigirey M.; Day T.; Annabhimoju R.; Sherman W. Protein and Ligand Preparation: Parameters, Protocols, and Influence on Virtual Screening Enrichments. J. Comput. Aided. Mol. Des. 2013, 27 (3), 221–234. 10.1007/s10822-013-9644-8. 23579614

[ref28] Schrödinger Release 2022–1: Protein Preparation Wizard; Epik; Schrödinger, LLC, New York, NY, 2021; Impact, Schrödinger, LLC, New York, NY; Prime, Schrödinger, LLC, New York, NY, 2021.

[ref29] Schrödinger Release 2022–1: Maestro; Schrödinger, LLC, New York, NY, 2021.

[ref30] Schrödinger Release 2022–1: Prime; Schrödinger, LLC, New York, NY, 2021.

[ref31] Jacobson M. P.; Pincus D. L.; Rapp C. S.; Day T. J. F.; Honig B.; Shaw D. E.; Friesner R. A. A Hierarchical Approach to All-Atom Protein Loop Prediction. Proteins Struct. Funct. Bioinforma. 2004, 55 (2), 351–367. 10.1002/prot.10613. 15048827

[ref32] Jacobson M. P.; Friesner R. A.; Xiang Z.; Honig B. On the Role of the Crystal Environment in Determining Protein Side-Chain Conformations. J. Mol. Biol. 2002, 320 (3), 597–608. 10.1016/S0022-2836(02)00470-9. 12096912

[ref33] Olsson M. H. M.; Søndergaard C. R.; Rostkowski M.; Jensen J. H. PROPKA3: Consistent Treatment of Internal and Surface Residues in Empirical PKa Predictions. J. Chem. Theory Comput. 2011, 7 (2), 525–537. 10.1021/ct100578z. 26596171

[ref34] Lu C.; Wu C.; Ghoreishi D.; Chen W.; Wang L.; Damm W.; Ross G. A.; Dahlgren M. K.; Russell E.; Von Bargen C. D.; Abel R.; Friesner R. A.; Harder E. D. OPLS4: Improving Force Field Accuracy on Challenging Regimes of Chemical Space. J. Chem. Theory Comput. 2021, 17 (7), 4291–4300. 10.1021/acs.jctc.1c00302. 34096718

[ref35] Jorgensen W. L.; Tirado-Rives J. The OPLS [Optimized Potentials for Liquid Simulations] Potential Functions for Proteins, Energy Minimizations for Crystals of Cyclic Peptides and Crambin. J. Am. Chem. Soc. 1988, 110 (6), 1657–1666. 10.1021/ja00214a001. 27557051

[ref36] Jorgensen W. L.; Maxwell D. S.; Tirado-Rives J. Development and Testing of the OPLS All-Atom Force Field on Conformational Energetics and Properties of Organic Liquids. J. Am. Chem. Soc. 1996, 118 (45), 11225–11236. 10.1021/ja9621760.

[ref37] Shivakumar D.; Williams J.; Wu Y.; Damm W.; Shelley J.; Sherman W. Prediction of Absolute Solvation Free Energies Using Molecular Dynamics Free Energy Perturbation and the OPLS Force Field. J. Chem. Theory Comput. 2010, 6 (5), 1509–1519. 10.1021/ct900587b. 26615687

[ref38] Schrödinger Release 2022–1: LigPrep; Schrödinger, LLC: New York, NY, 2021.

[ref39] Shelley J. C.; Cholleti A.; Frye L. L.; Greenwood J. R.; Timlin M. R.; Uchimaya M. Epik: A Software Program for PKaprediction and Protonation State Generation for Drug-like Molecules. J. Comput. Aided. Mol. Des. 2007, 21 (12), 681–691. 10.1007/s10822-007-9133-z. 17899391

[ref40] Greenwood J. R.; Calkins D.; Sullivan A. P.; Shelley J. C. Towards the Comprehensive, Rapid, and Accurate Prediction of the Favorable Tautomeric States of Drug-like Molecules in Aqueous Solution. J. Comput. Aided. Mol. Des. 2010, 24 (6), 591–604. 10.1007/s10822-010-9349-1. 20354892

[ref41] Schrödinger Release 2022–1: Epik; Schrödinger, LLC, New York, NY, 2021.

[ref42] Friesner R. A.; Banks J. L.; Murphy R. B.; Halgren T. A.; Klicic J. J.; Mainz D. T.; Repasky M. P.; Knoll E. H.; Shelley M.; Perry J. K.; Shaw D. E.; Francis P.; Shenkin P. S. Glide: A New Approach for Rapid, Accurate Docking and Scoring. 1. Method and Assessment of Docking Accuracy. J. Med. Chem. 2004, 47 (7), 1739–1749. 10.1021/jm0306430. 15027865

[ref43] Halgren T. A.; Murphy R. B.; Friesner R. A.; Beard H. S.; Frye L. L.; Pollard W. T.; Banks J. L. Glide: A New Approach for Rapid, Accurate Docking and Scoring. 2. Enrichment Factors in Database Screening. J. Med. Chem. 2004, 47 (7), 1750–1759. 10.1021/jm030644s. 15027866

[ref44] Friesner R. A.; Murphy R. B.; Repasky M. P.; Frye L. L.; Greenwood J. R.; Halgren T. A.; Sanschagrin P. C.; Mainz D. T. Extra Precision Glide: Docking and Scoring Incorporating a Model of Hydrophobic Enclosure for Protein-Ligand Complexes. J. Med. Chem. 2006, 49 (21), 6177–6196. 10.1021/jm051256o. 17034125

[ref45] Schrödinger Release 2022–1: Glide; Schrödinger, LLC, New York, NY, 2021.

[ref46] Bowers K. J.; Chow D. E.; Xu H.; Dror R. O.; Eastwood M. P.; Gregersen B. A.; Klepeis J. L.; Kolossvary I.; Moraes M. A.; Sacerdoti F. D.; Salmon J. K.; Shan Y.; Shaw D. E. Scalable Algorithms for Molecular Dynamics Simulations on Commodity Clusters. In SC ’06: Proceedings of the 2006 ACM/IEEE Conference on Supercomputing; 2006; p 43.

[ref47] Schrödinger Release 2022–1: Desmond Molecular Dynamics System; D. E. Shaw Research, New York, NY, 2021. Maestro: Desmond Interoperability Tools; Schrödinger, New York, NY, 2021.

[ref48] Jorgensen W. L.; Chandrasekhar J.; Madura J. D.; Impey R. W.; Klein M. L. Comparison of Simple Potential Functions for Simulating Liquid Water. J. Chem. Phys. 1983, 79 (2), 926–935. 10.1063/1.445869.

[ref49] Martyna G. J.; Klein M. L.; Tuckerman M. Nosé-Hoover Chains: The Canonical Ensemble via Continuous Dynamics. J. Chem. Phys. 1992, 97 (4), 2635–2643. 10.1063/1.463940.

[ref50] Wentzcovitch R. M. Invariant Molecular-Dynamics Approach to Structural Phase Transitions. Phys. Rev. B 1991, 44 (5), 2358–2361. 10.1103/PhysRevB.44.2358. 9999791

[ref51] Nosé S. A Unified Formulation of the Constant Temperature Molecular Dynamics Methods. J. Chem. Phys. 1984, 81, 511–519. 10.1063/1.447334.

[ref52] Li J.; Abel R.; Zhu K.; Cao Y.; Zhao S.; Friesner R. A. The VSGB 2.0 Model: A next Generation Energy Model for High Resolution Protein Structure Modeling. Proteins Struct. Funct. Bioinforma. 2011, 79 (10), 2794–2812. 10.1002/prot.23106. PMC3206729 21905107

