# Supplementary material for: Binding Analysis and Structure-Based Design of Tricyclic Coumarin-Derived MTHFD2 Inhibitors as Anticancer Agents: Insights from Computational Modeling
Source: ACS Omega. 2023 Apr 12;8(16):14440–58. doi: 10.1021/acsomega.2c08025 (PMC10134251; doi:10.1021/acsomega.2c08025)
Supplement: Supplementary file 1 — ao2c08025_si_001.pdf [file ao2c08025_si_001.pdf]

SUPPORTING INFORMATION

Binding analysis and structure-based design of tricyclic coumarin-derived MTHFD2 inhibitors as anticancer agents: Insights from computational modeling

Vibhu Jha, Fredrik Lannestam Holmelin and Leif A. Eriksson*

Department of Chemistry and Molecular Biology, University of Gothenburg, 405 30 Göteborg, Sweden

Correspondence:

*Leif A. Eriksson, Department of Chemistry and Molecular Biology,
University of Gothenburg, 405 30 Göteborg, Sweden.
Email: leif.eriksson@chem.gu.se

Table of Content

Figure S1. Protein-ligand interaction histogram from MD simulations of compound 1 in the binding site of MTHFD2. S4

Figure S2. Protein-ligand interaction histogram from MD simulations of compound 2 in the binding site of MTHFD2. S4

Figure S3. Protein-ligand interaction histogram from MD simulations of compound 3 in the binding site of MTHFD2. S5

Figure S4. Superposed MD trajectory snapshots of compound 4 at 36th ns and 164th ns in the MTHFD2 binding site, showing conformational changes. S5

Figure S5. Protein-ligand interaction histogram from MD simulations of compound 4 in the binding site of MTHFD2. S6

Figure S6. Docking pose of compound 1 in the MTHFD1 binding site, relative to the crystallographic pose of compound 5. S6

Figure S7. Docking pose of compound 2 in the MTHFD1 binding site, relative to the crystallographic pose of compound 5. S7

Figure S8. Docking pose of compound 3 in the MTHFD1 binding site, relative to the crystallographic pose of compound 5. S7

Figure S9. Docking pose of compound 4 in the MTHFD1 binding site, relative to the crystallographic pose of compound 5. S8

Figure S10. Docking pose of compound 10 in the MTHFD1 binding site, relative to the crystallographic pose of compound 5. S8

Figure S11. Docking pose of compound 11 in the MTHFD1 binding site, relative to the crystallographic pose of compound 5. S9

Figure S12. Docking pose of compound 12 in the MTHFD1 binding site, relative to the crystallographic pose of compound 5. S9

Figure S13. Docking pose of compound 13 in the MTHFD1 binding site, relative to the crystallographic pose of compound 5. S10

Figure S14. Protein-ligand interaction histogram from the MD simulations of compound 10 in the binding site of MTHFD2. S10

Figure S15. Protein-ligand interaction histogram from MD simulations of compound 11 in the binding site of MTHFD2. S11

Figure S16. Protein-ligand interaction histogram from the MD simulations of compound 12 in the binding site of MTHFD2. S11

Figure S17. Protein-ligand interaction histogram from the MD simulations of compound 13 in the binding site of MTHFD2. S12

Figure S18. Protein-ligand interaction histogram from the MD simulations of compound 5 in the binding site of MTHFD1. S12

Figure S19. Protein-ligand interaction histogram from the MD simulations of compound 1 in the binding site of MTHFD1. S13

Figure S20. Protein-ligand interaction histogram from the MD simulations of compound 2 in the binding site of MTHFD1. S13

Figure S21. Protein-ligand interaction histogram from the MD simulations of compound 3 in the binding site of MTHFD1. S14

Figure S22. Protein-ligand interaction histogram from the MD simulations of compound 4 in the binding site of MTHFD1. S14

Figure S23. Protein-ligand interaction histogram from the MD simulations of compound 10 in the binding site of MTHFD1. S15

Figure S24. Protein-ligand interaction histogram from the MD simulations of compound 11 in the MTHFD1 binding site of MTHFD1. S15

Figure S25. Protein-ligand interaction histogram from the MD simulations of compound 12 in the binding site of MTHFD1. S16

Figure S26. Protein-ligand interaction histogram from the MD simulations of compound 13 in the binding site of MTHFD1. S16

Figure S27. 2D structures of the 18 selected potential inhibitors of MTHFD2 from the ADME analysis (compounds 10-27). S17

Figure S28. 2D structures of selected 145 compounds from structure-based drug design approach (compounds 10-154) S18 – S21

Table S1. Computed pharmacokinetic and physicochemical properties of the existing tricyclic coumarin-based MTHFD2 inhibitors (compounds 1-4) and the 18 selected potential MTHFD2 inhibitors (compound 10-27). S22

Table S2. SMILES and Glide scores of selected 145 compounds in the MTHFD2 and MTHFD1 binding sites (compounds 10-154). S23 – S26

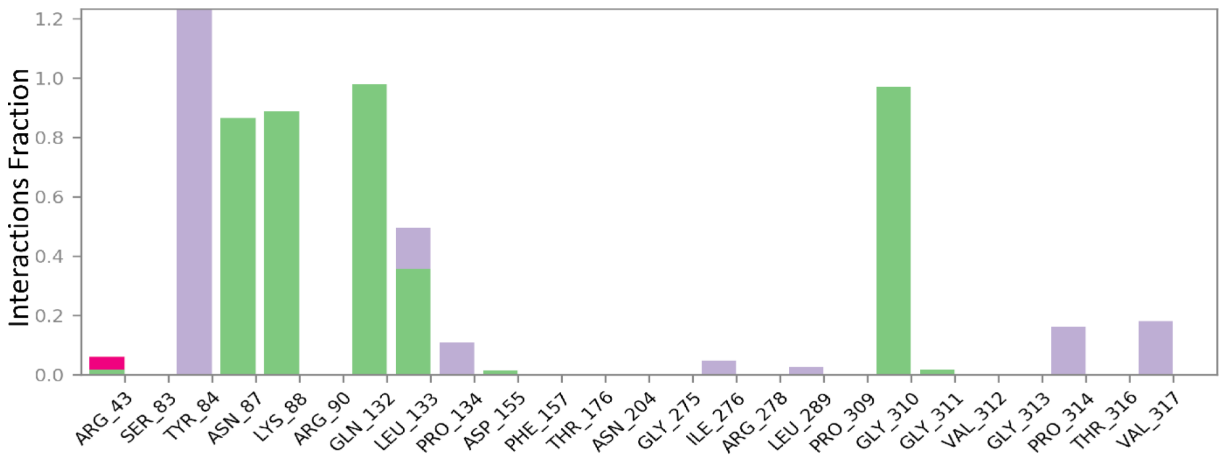

Figure S1. Protein-ligand interaction histogram from the MD simulations of compound 1 in the MTHFD2 binding site. (H-bonds are shown in green, and lipophilic contacts are shown in grey).

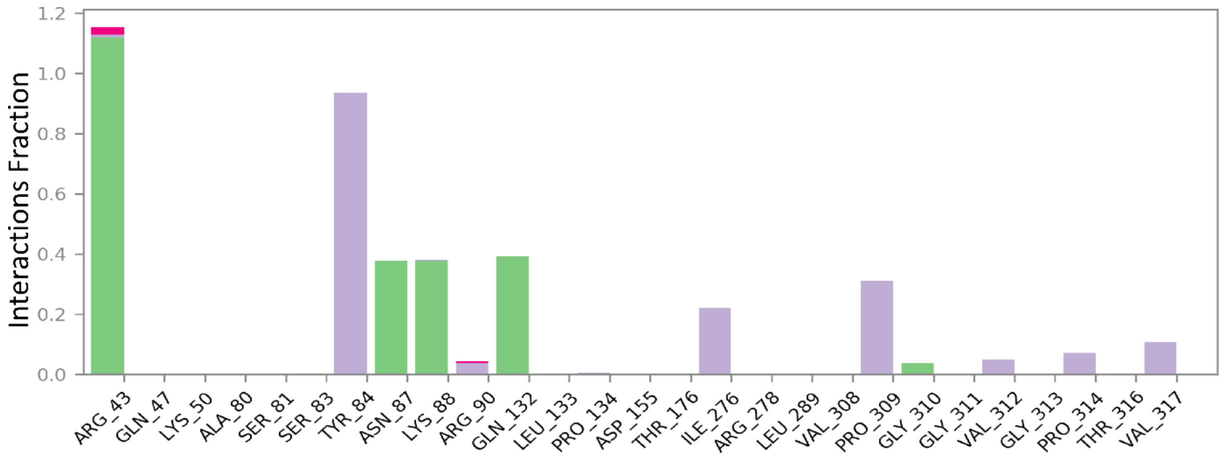

Figure S2. Protein-ligand interaction histogram from the MD simulations of compound 2 in the MTHFD2 binding site. (H-bonds are shown in green, salt-bridge interactions are shown in pink, and lipophilic contacts are shown in grey).

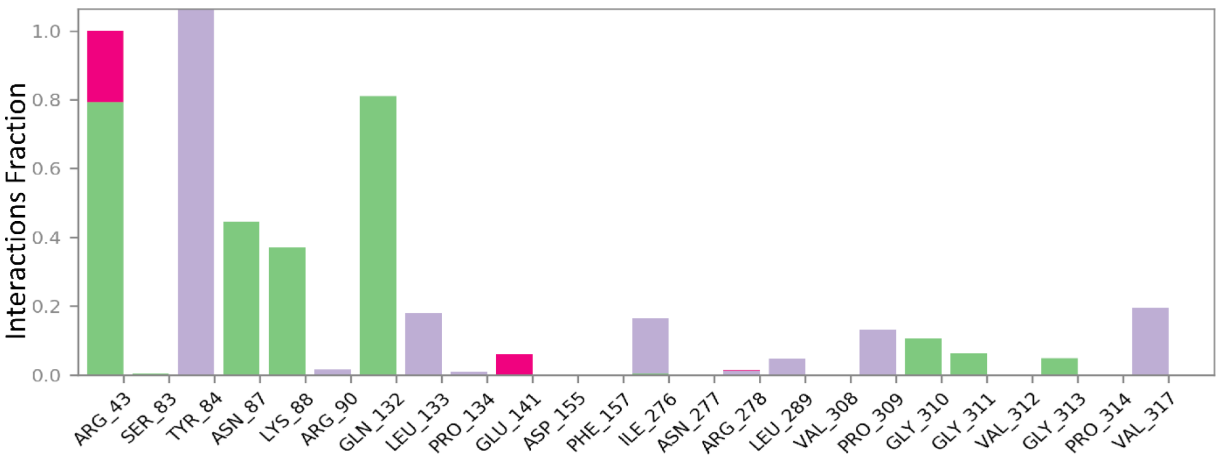

Figure S3. Protein-ligand interaction histogram from the MD simulations of compound 3 in the MTHFD2 binding site. (H-bonds are shown in green, salt-bridge interactions are shown in pink, and lipophilic contacts are shown in grey).

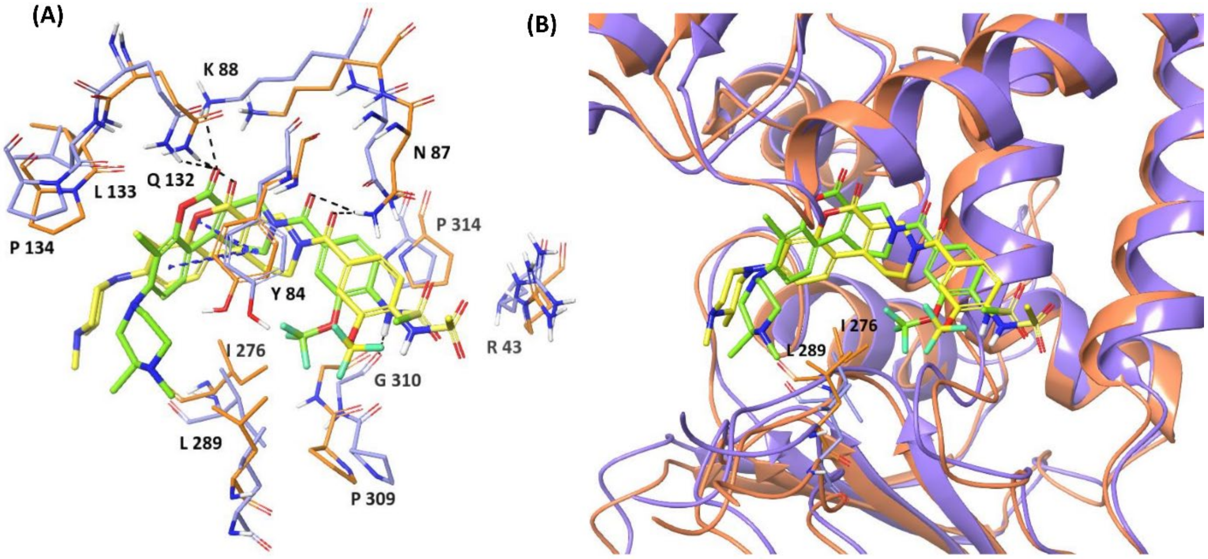

Figure S4. (A) Superposed MD trajectory snapshots of compound 4 at 36th ns (yellow) and 164th ns (green) in the MTHFD2 binding site (protein residues in blue for 36th ns, orange for 164th ns). **(B)** Ribbon view: protein ribbons in blue at 36th ns, in orange at 164th ns. Only Ile276 and Leu289 are shown while other residues are hidden.

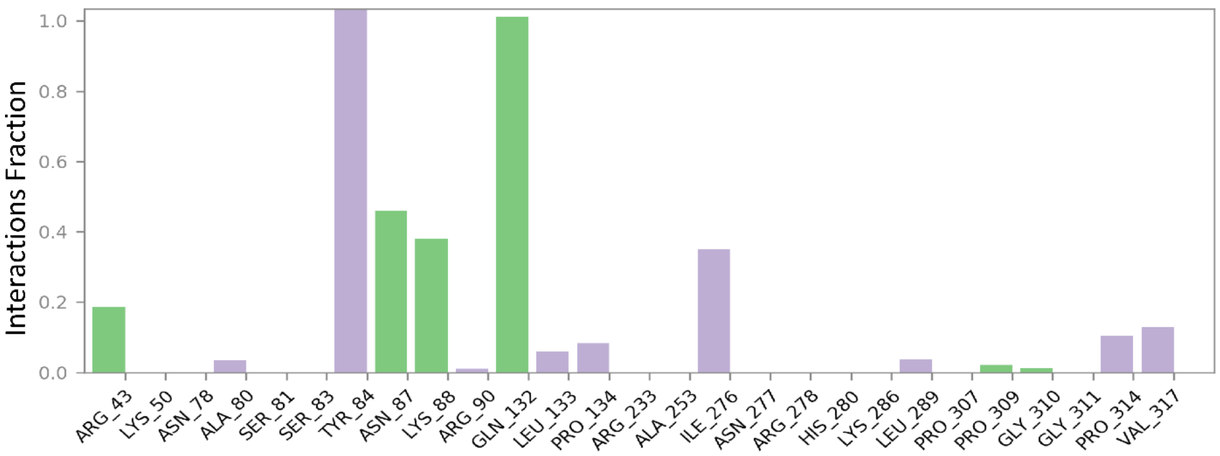

Figure S5. Protein-ligand interaction histogram from the MD simulations of compound 4 in the MTHFD2 binding site. (H-bonds are shown in green and, lipophilic contacts are shown in grey).

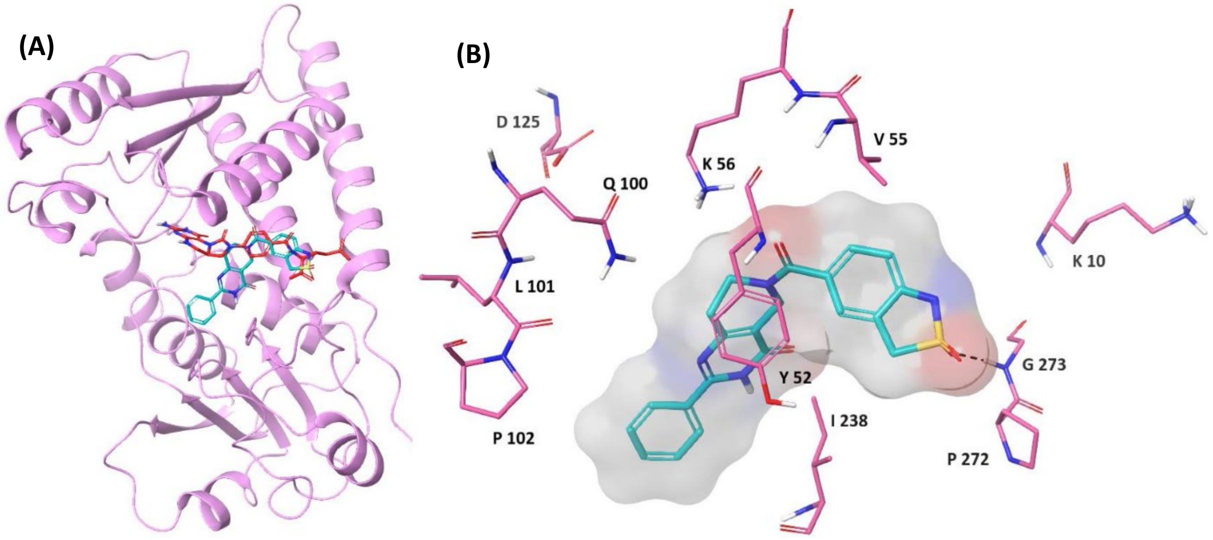

Figure S6. Docking pose of compound 1 (A. ribbon view, B. binding site view), in the MTHFD1 binding site, relative to the crystallographic pose of compound 5. Ribbons, protein residues are colored in pink, compound 5 in red and compound 1 in cyan.

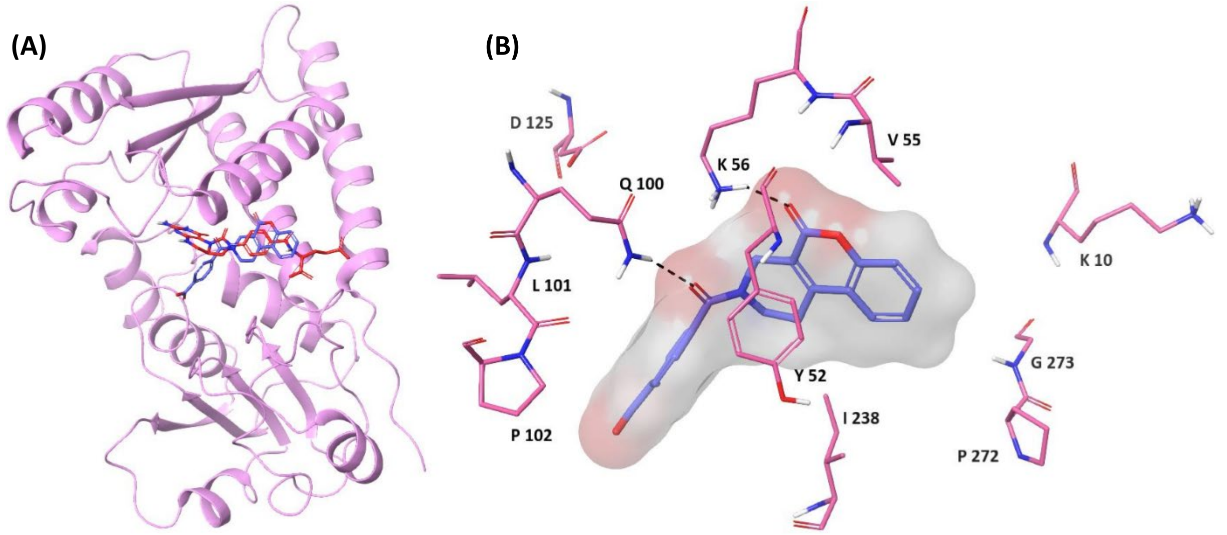

Figure S7. Docking pose of compound 2 (A. ribbon view, B. binding site view), in the MTHFD1 binding site, relative to the crystallographic pose of compound 5. Ribbons, protein residues are colored in pink, compound 5 in red and compound 2 in blue.

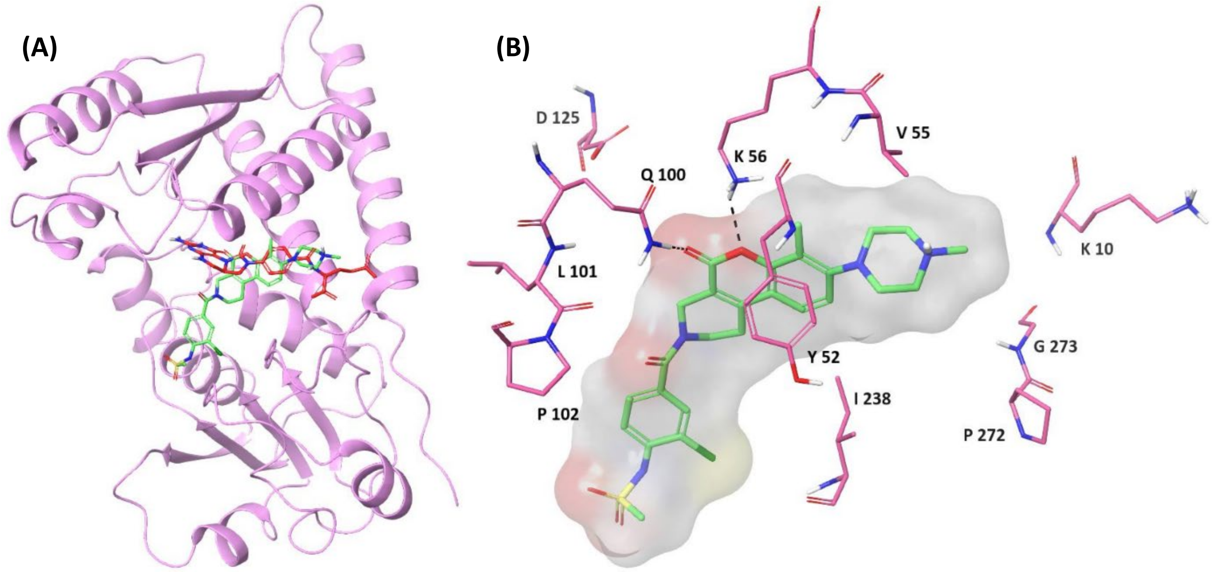

Figure S8. Docking pose of compound 3 (A. ribbon view, B. binding site view), in the MTHFD1 binding site, relative to the crystallographic pose of compound 5. Ribbons, protein residues are colored in pink, compound 5 in red and compound 3 in light green.

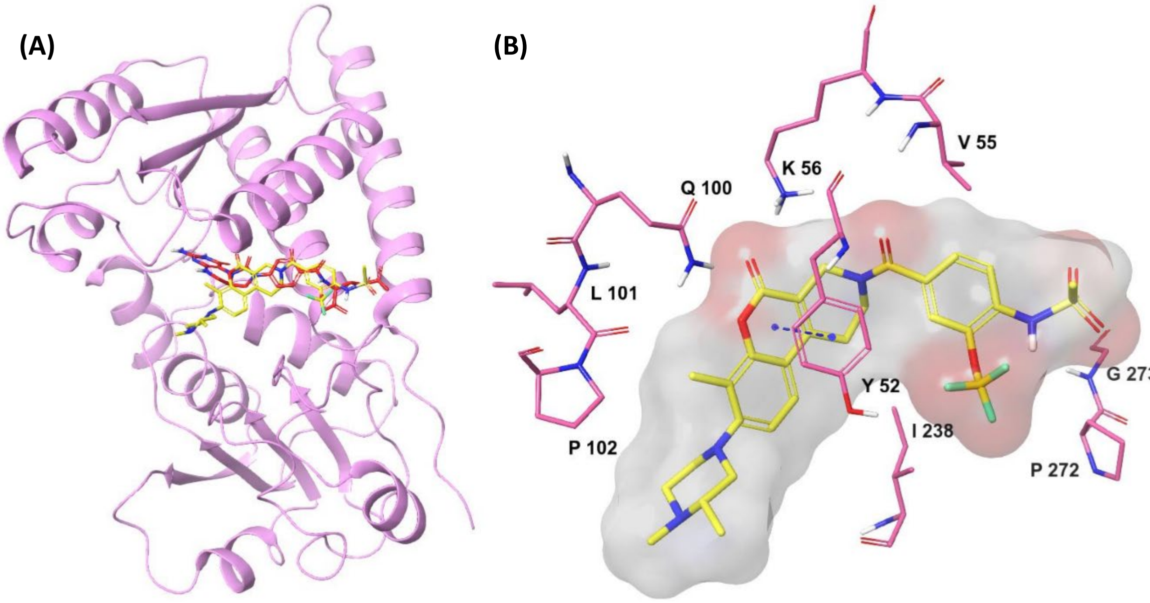

Figure S9. Docking pose of compound 4 (A. ribbon view, B. binding site view), in the MTHFD1 binding site, relative to the crystallographic pose of compound 5. Ribbons, protein residues are colored in pink, compound 5 in red and compound 4 in yellow.

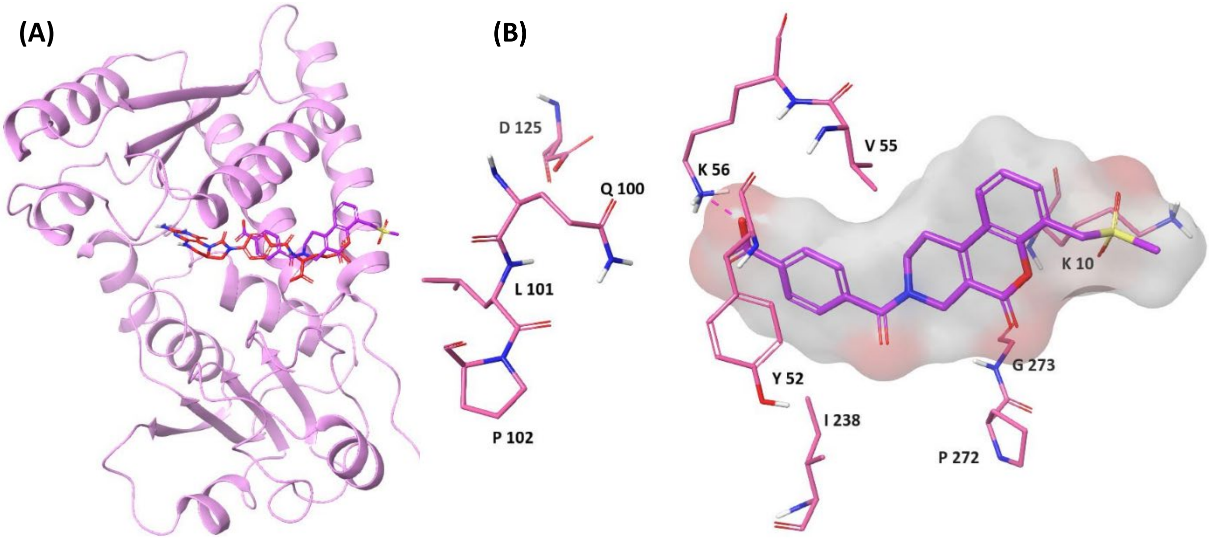

Figure S10. Docking pose of compound 10 (A. ribbon view, B. binding site view), in the MTHFD1 binding site, relative to the crystallographic pose of compound 5. Ribbons, protein residues are colored in pink, compound 5 in red and compound 10 in purple.

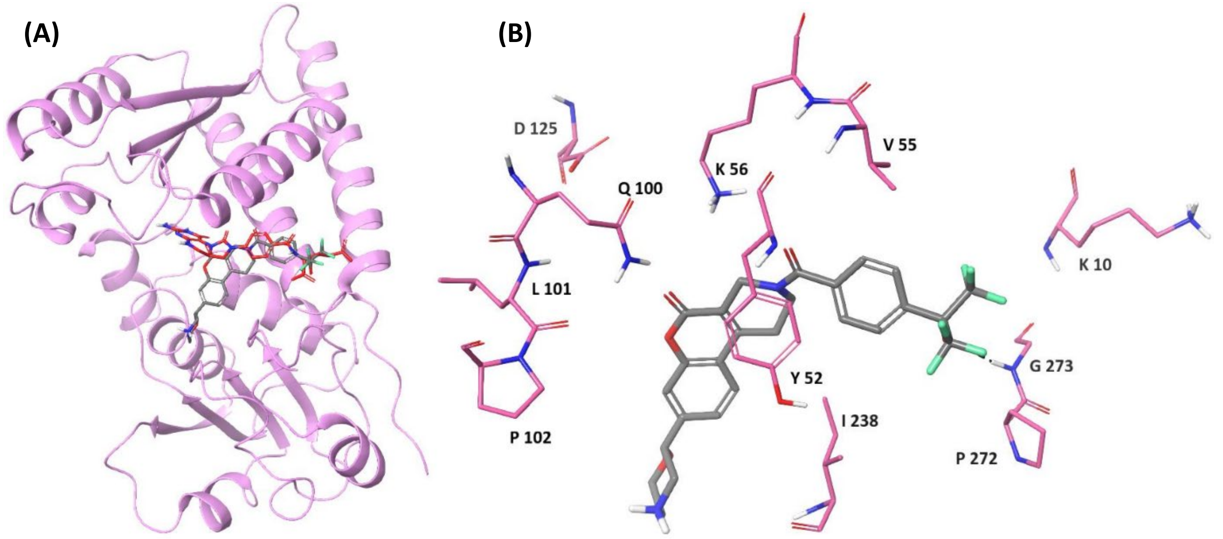

Figure S11. Docking pose of compound 11 (A. ribbon view, B. binding site view), in the MTHFD1 binding site, relative to the crystallographic pose of compound 5. Ribbons, protein residues are colored in pink, compound 5 in red and compound 11 in grey.

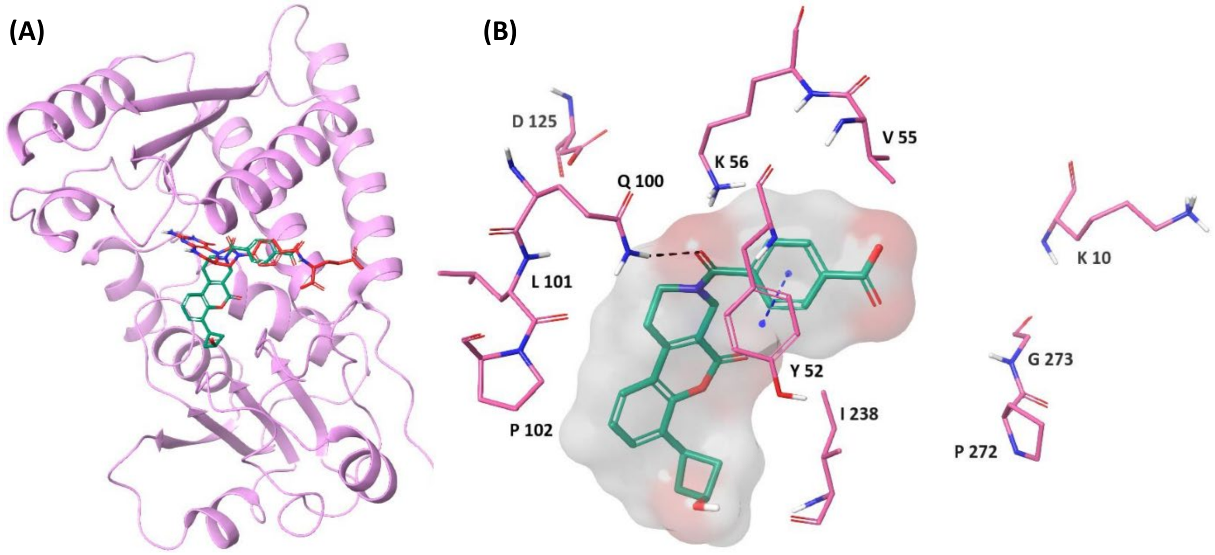

Figure S12. Docking pose of compound 12 (A. ribbon view, B. binding site view), in the MTHFD1 binding site, relative to the crystallographic pose of compound 5. Ribbons, protein residues are colored in pink, compound 5 in red and compound 12 in dark green.

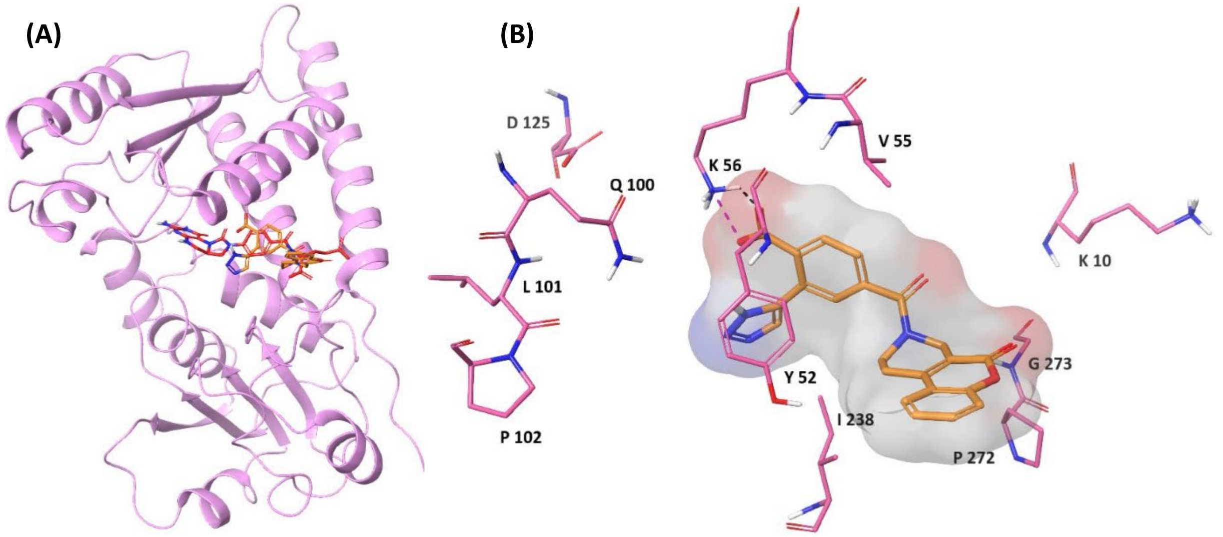

Figure S13. Docking pose of compound 13 (A. ribbon view, B. binding site view), in the MTHFD1 binding site, relative to the crystallographic pose of compound 5. Ribbons, protein residues are colored in pink, compound 5 in red and compound 13 in orange.

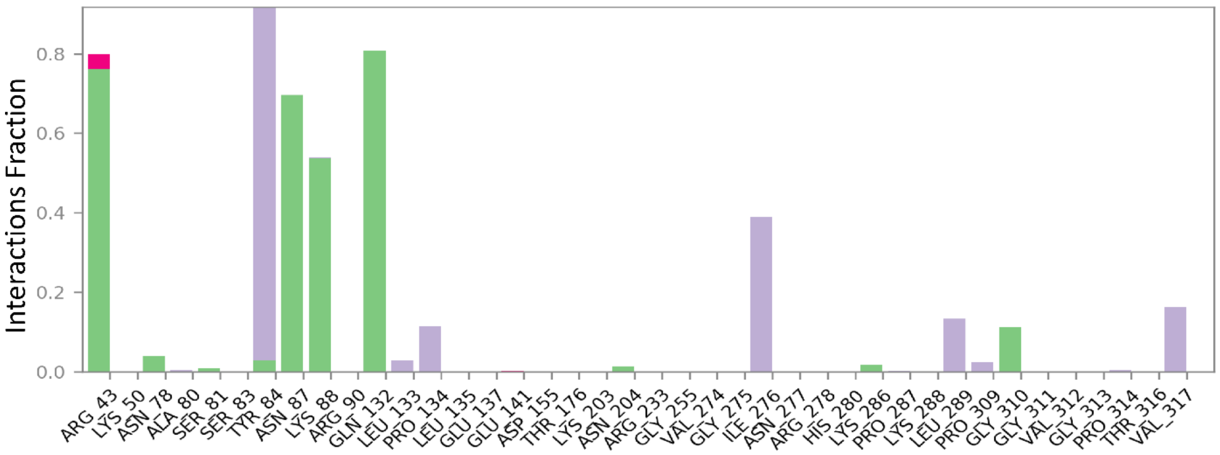

Figure S14. Protein-ligand interaction histogram from the MD simulations of compound 10 in the MTHFD2 binding site. (H-bonds are shown in green and, lipophilic contacts are shown in grey).

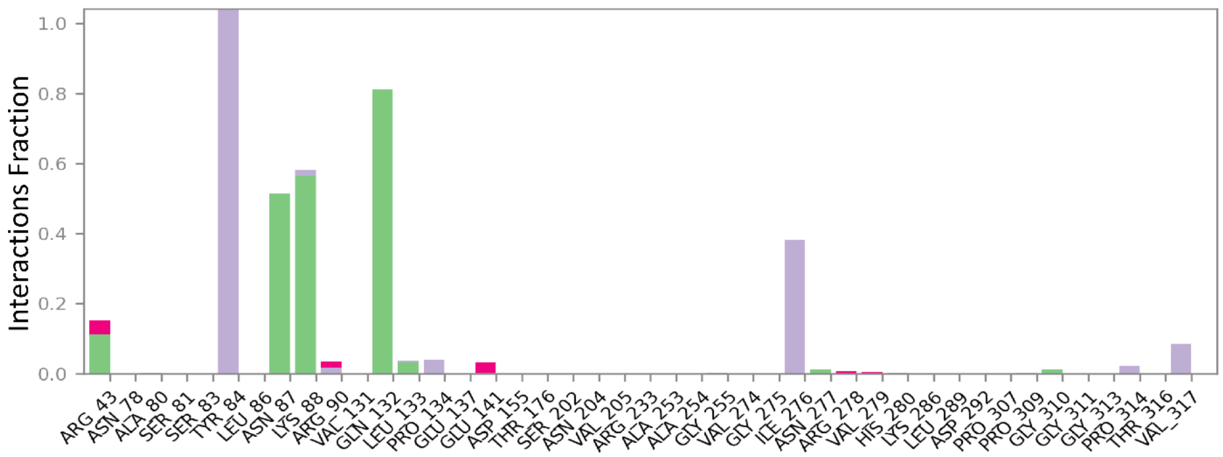

Figure S15. Protein-ligand interaction histogram from the MD simulations of compound 11 in the MTHFD2 binding site. (H-bonds are shown in green and, lipophilic contacts are shown in grey).

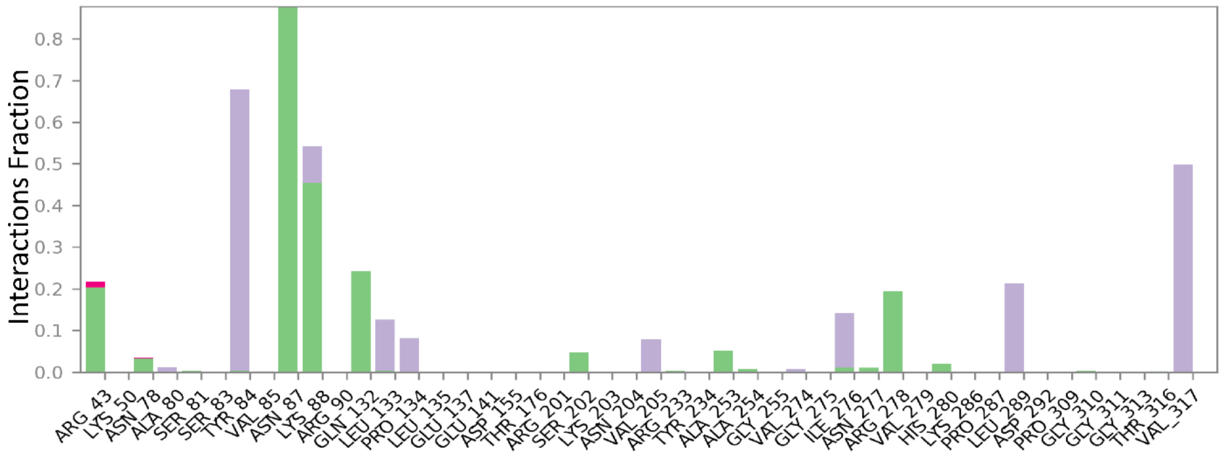

Figure S16. Protein-ligand interaction histogram from the MD simulations of compound 12 in the MTHFD2 binding site. (H-bonds are shown in green and, lipophilic contacts are shown in grey).

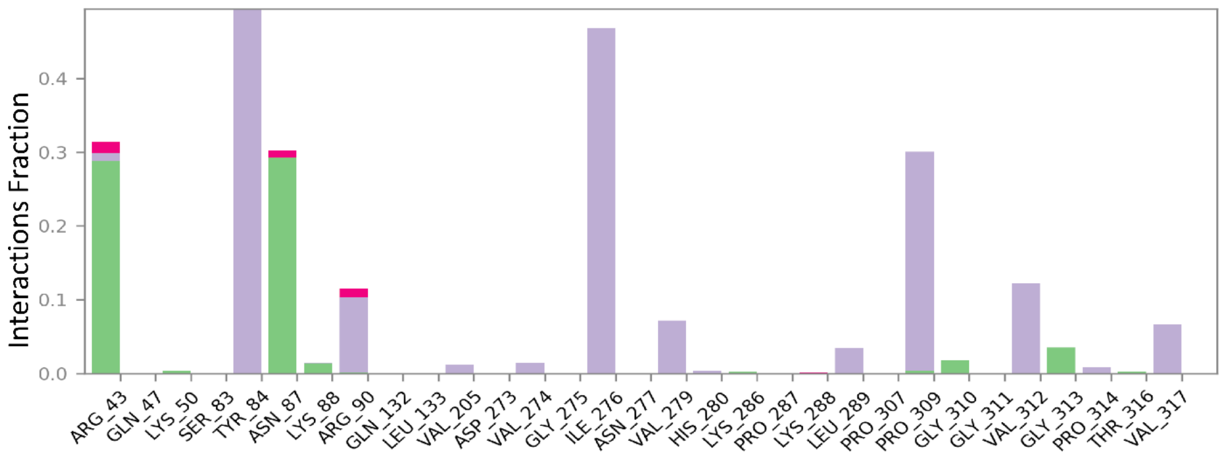

Figure S17. Protein-ligand interaction histogram from the MD simulations of compound 13 in the MTHFD2 binding site. (H-bonds are shown in green and, lipophilic contacts are shown in grey).

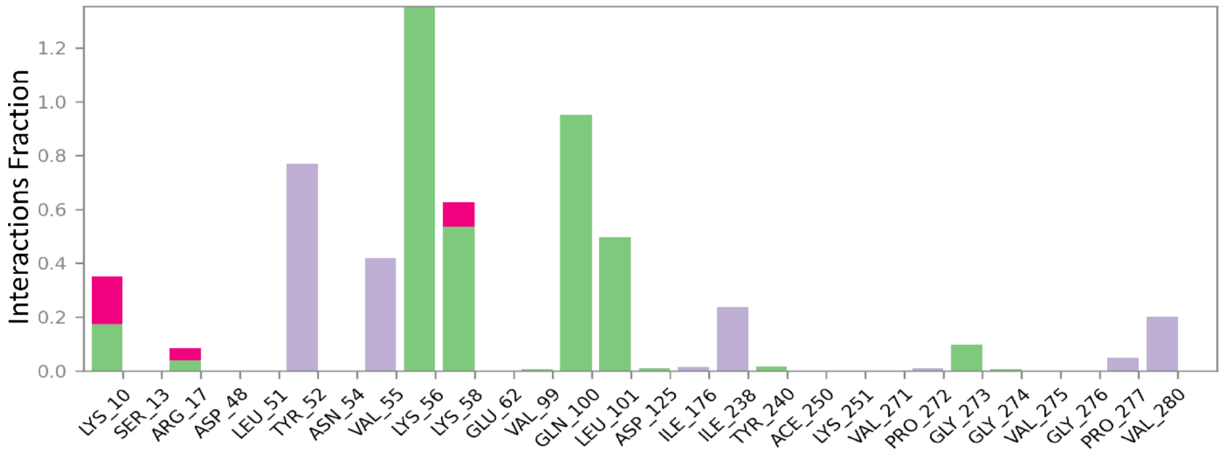

Figure S18. Protein-ligand interaction histogram from the MD simulations of compound 5 in the MTHFD1 binding site. (H-bonds are shown in green and, lipophilic contacts are shown in grey).

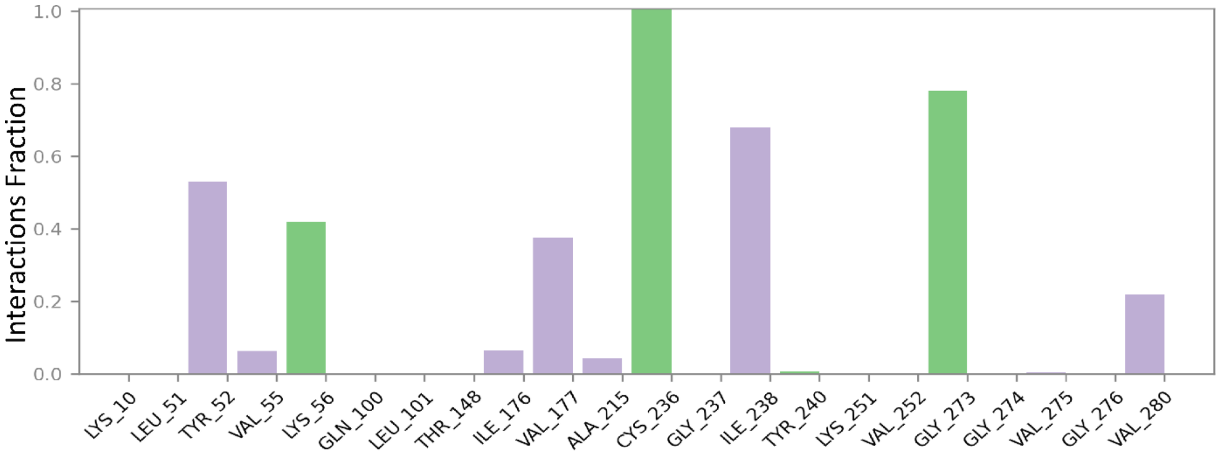

Figure S19. Protein-ligand interaction histogram from the MD simulations of compound 1 in the MTHFD1 binding site. (H-bonds are shown in green and, lipophilic contacts are shown in grey).

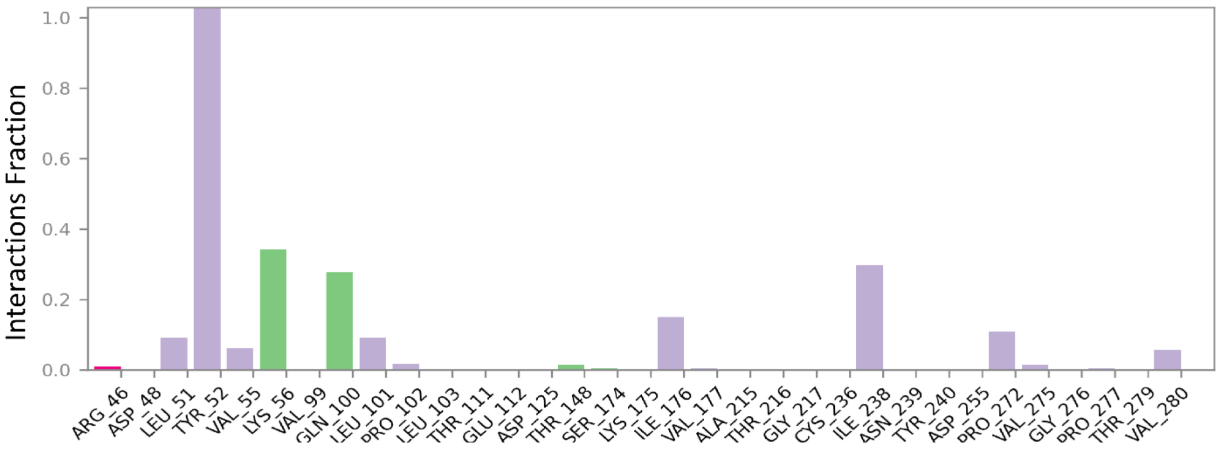

Figure S20. Protein-ligand interaction histogram from the MD simulations of compound 2 in the MTHFD1 binding site. (H-bonds are shown in green and, lipophilic contacts are shown in grey).

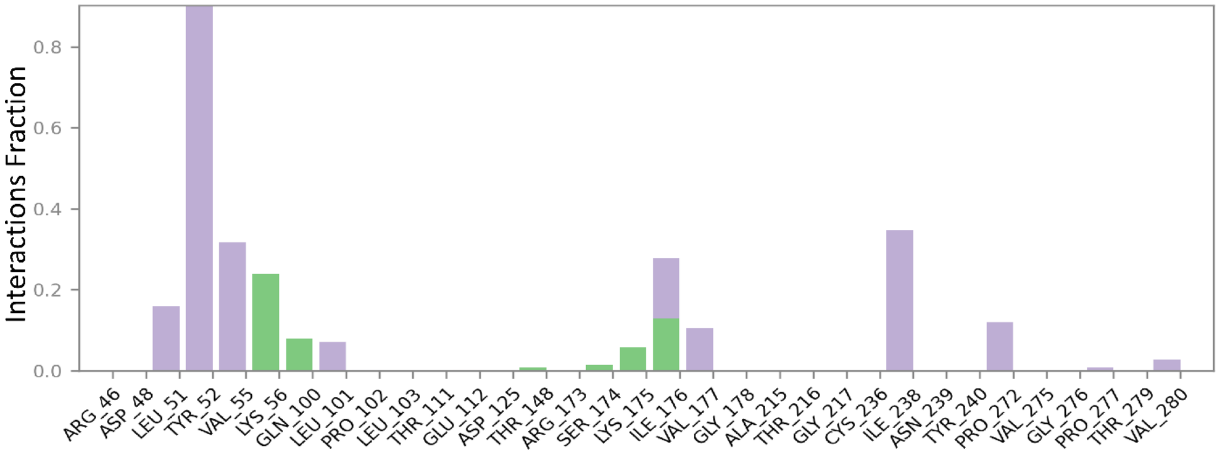

Figure S21. Protein-ligand interaction histogram from the MD simulations of compound 3 in the MTHFD1 binding site. (H-bonds are shown in green and, lipophilic contacts are shown in grey).

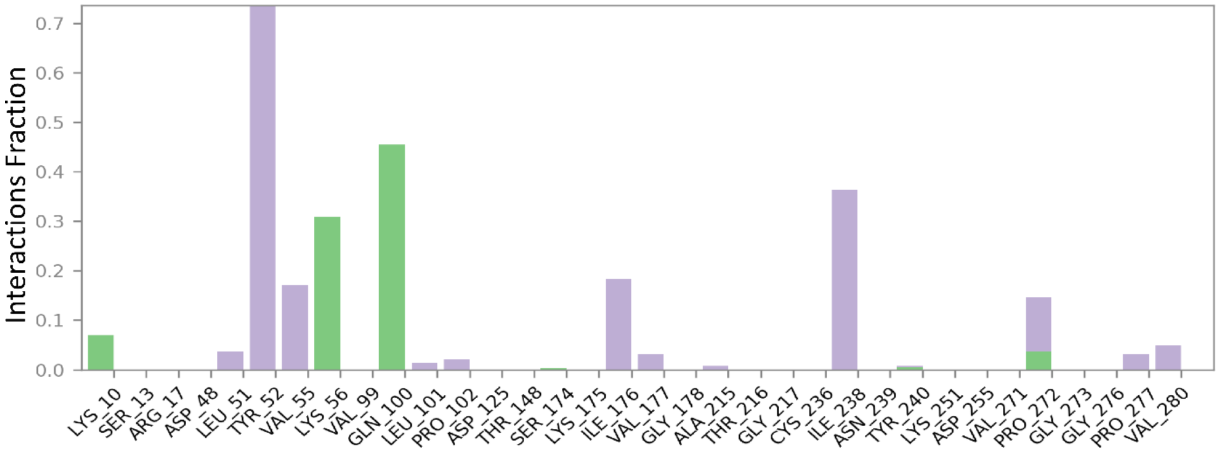

Figure S22. Protein-ligand interaction histogram from the MD simulations of compound 4 in the MTHFD1 binding site. (H-bonds are shown in green and, lipophilic contacts are shown in grey).

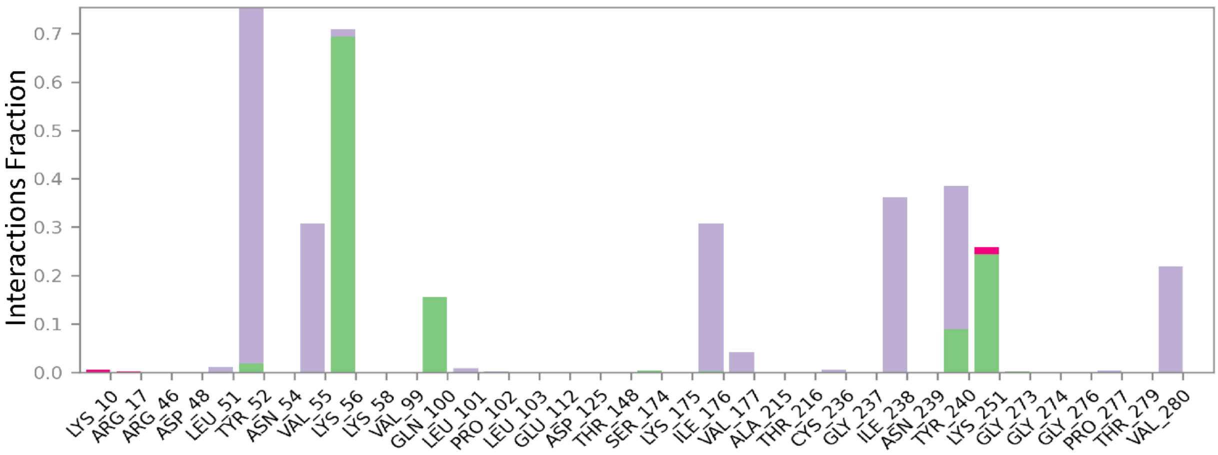

Figure S23. Protein-ligand interaction histogram from the MD simulations of compound 10 in the MTHFD1 binding site. (H-bonds are shown in green and, lipophilic contacts are shown in grey).

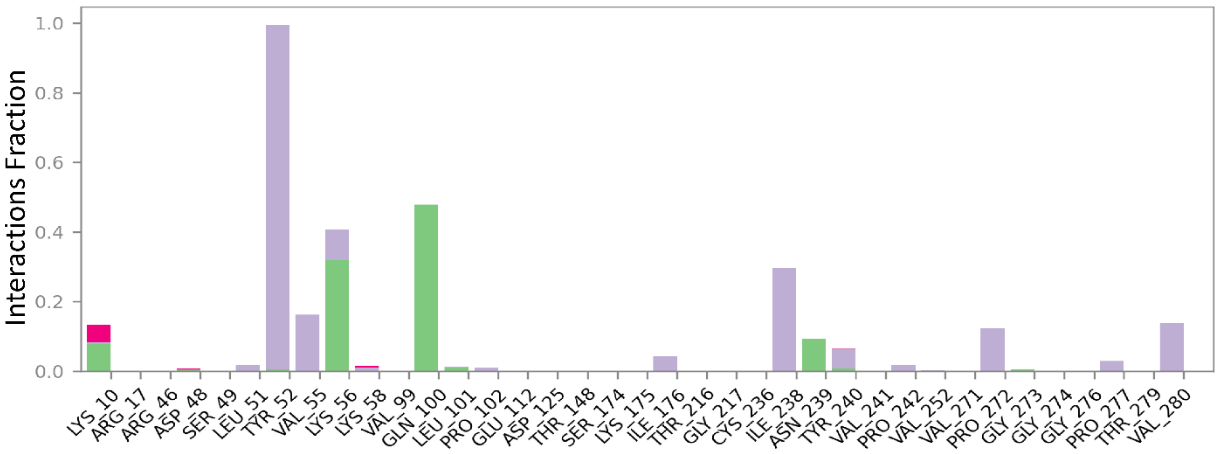

Figure S24. Protein-ligand interaction histogram from the MD simulations of compound 11 in the MTHFD1 binding site. (H-bonds are shown in green and, lipophilic contacts are shown in grey).

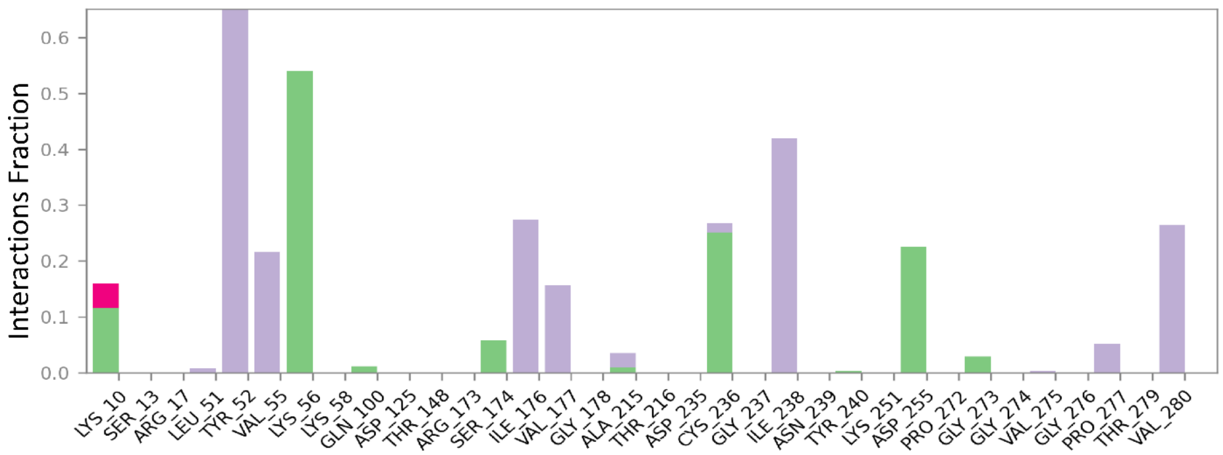

Figure S25. Protein-ligand interaction histogram from the MD simulations of compound 12 in the MTHFD1 binding site. (H-bonds are shown in green and, lipophilic contacts are shown in grey).

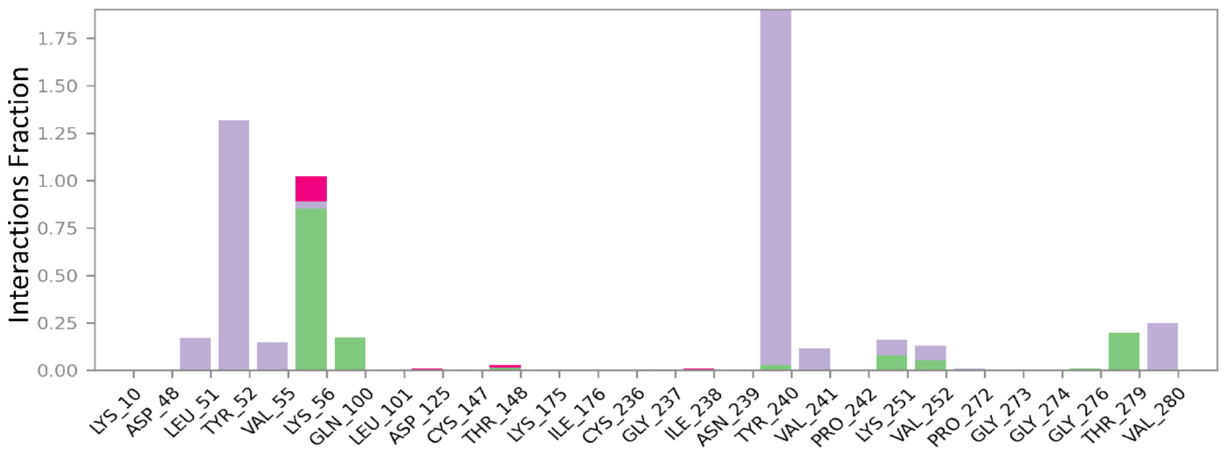

Figure S26. Protein-ligand interaction histogram from the MD simulations of compound 13 in the MTHFD1 binding site. (H-bonds are shown in green and, lipophilic contacts are shown in grey).

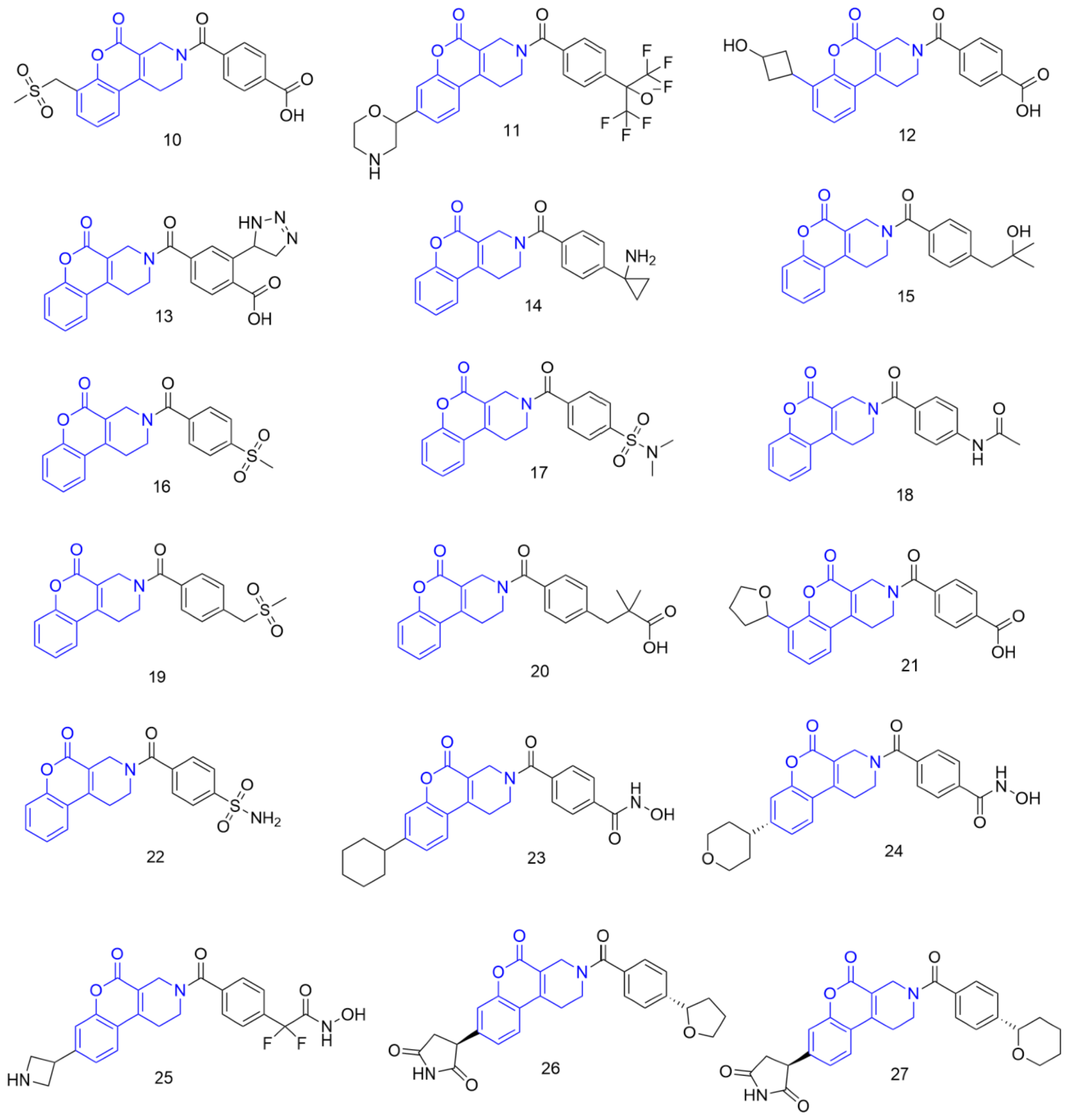

Figure S27. 2D structures of the 18 selected potential MTHFD2 inhibitors from ADME analysis (compound 10-27).

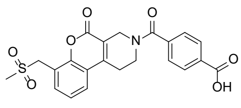
10
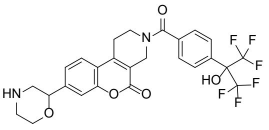
11
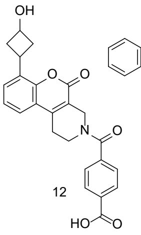
12
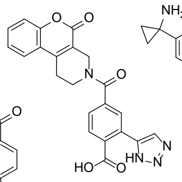
13
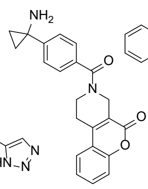
14
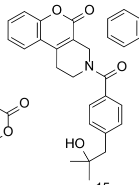
15
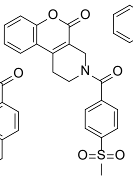
16
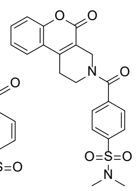
17
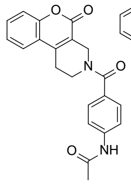
18
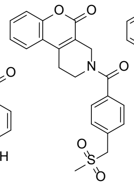
19
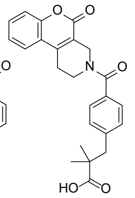
20
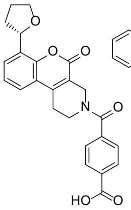
21
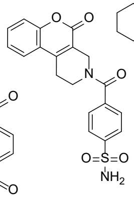
22
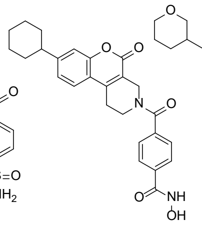
23
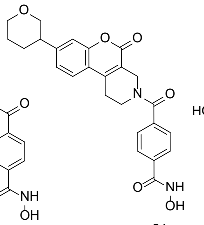
24
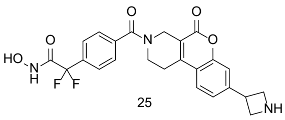
25
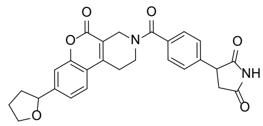
26
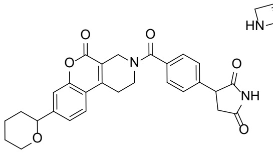
27
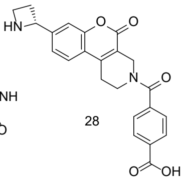
28
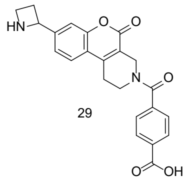
29
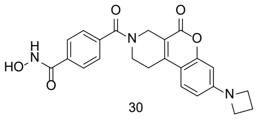
30
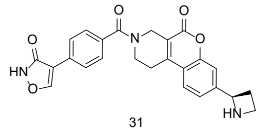
31
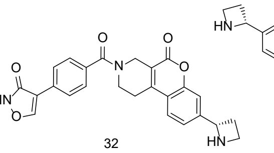
32
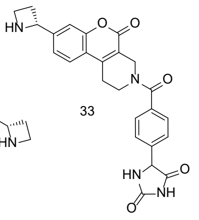
33
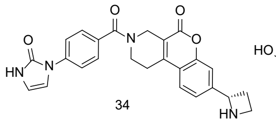
34
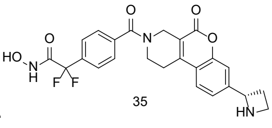
35
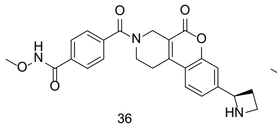
36
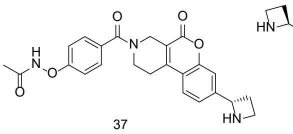
37
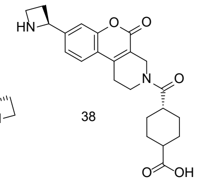
38
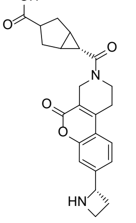
39
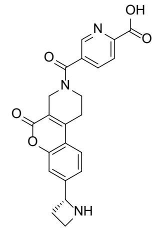
40
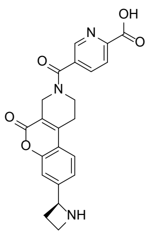
41
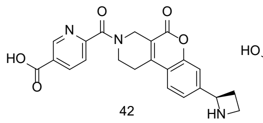
42
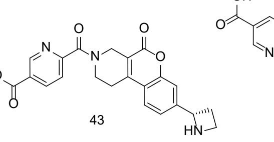
43
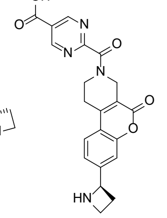
44
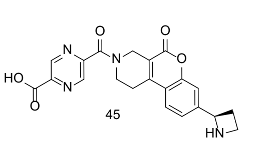
45
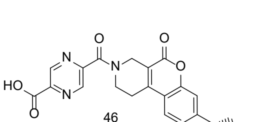
46

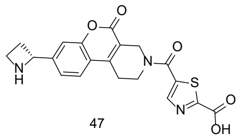
47

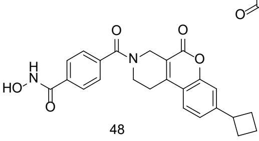
48

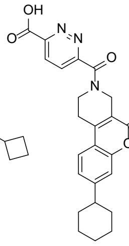
49

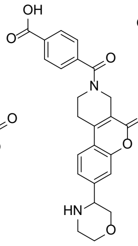
50

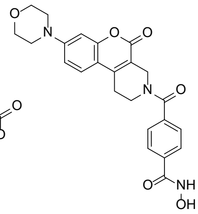
51

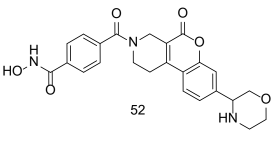
52

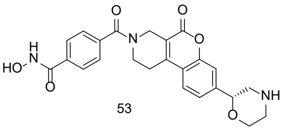
53

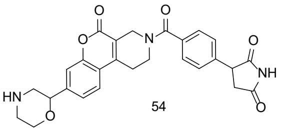
54

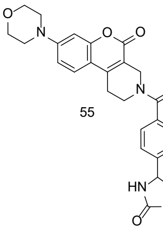
55

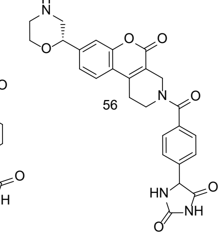
56

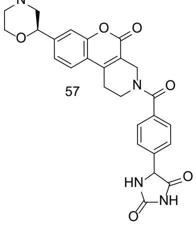
57

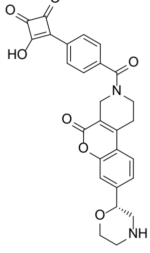
58

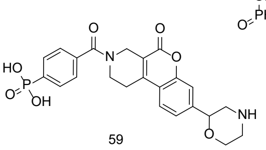
59

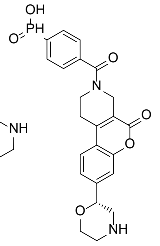
60

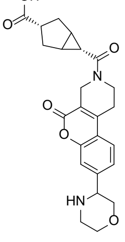
61

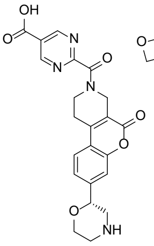
62

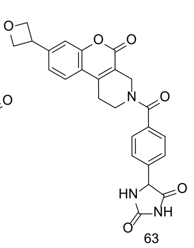
63

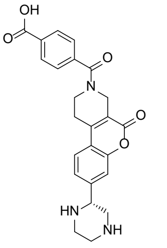
64

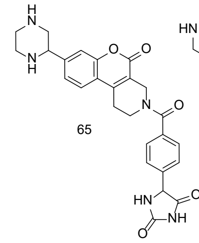
65

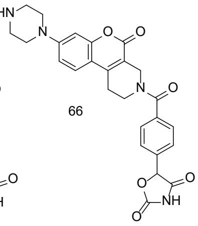
66

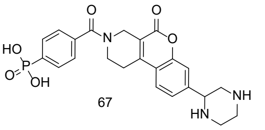
67

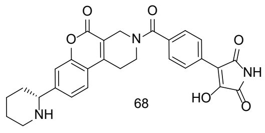
68

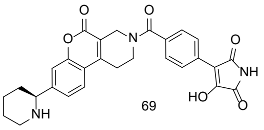
69

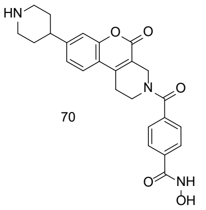
70

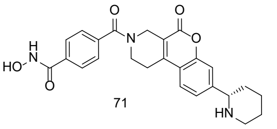
71

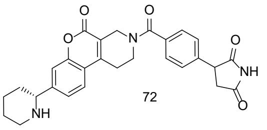
72

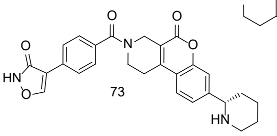
73

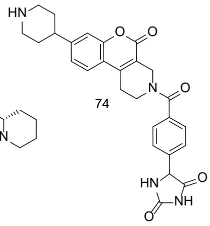
74

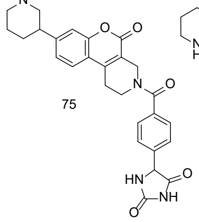
75

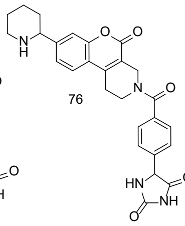
76

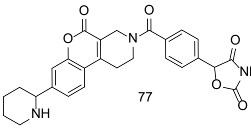
77

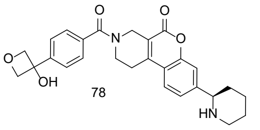
78

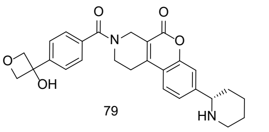
79

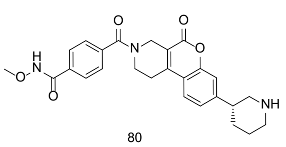
80

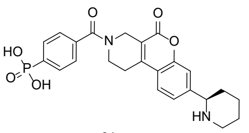
81

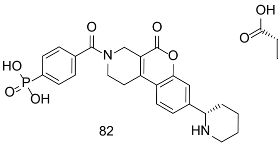
82

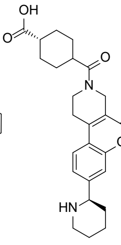
83

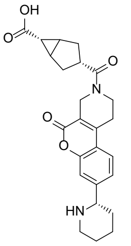
84

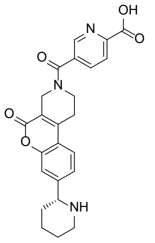
85

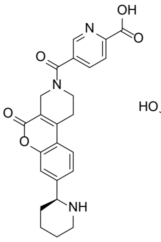
86

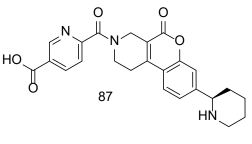
87

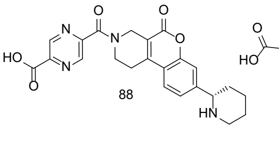
88

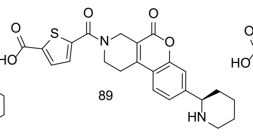
89

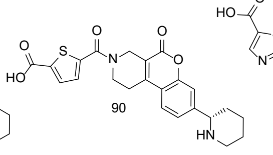
90

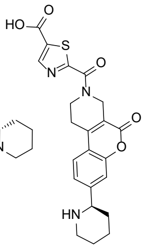
91

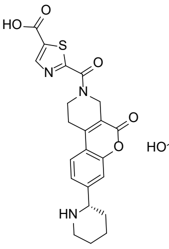
92

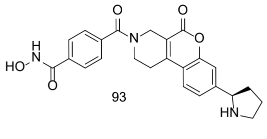
93

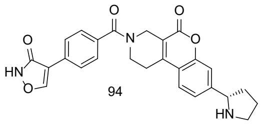
94

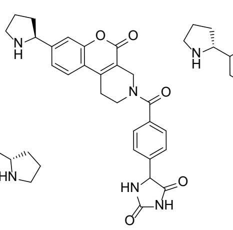
95

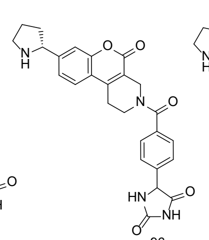
96

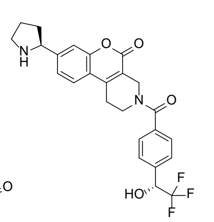
97

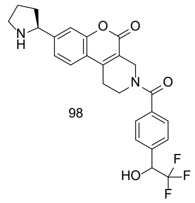
98

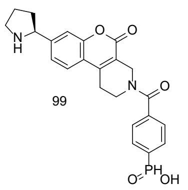
99

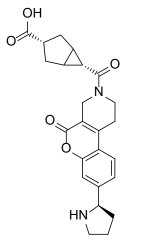
100

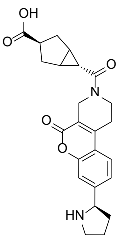
101

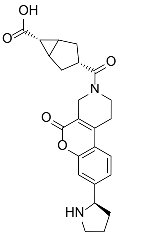
102

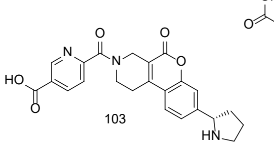
103

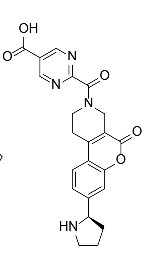
104

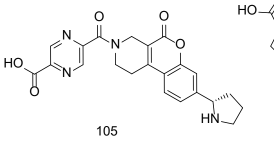
105

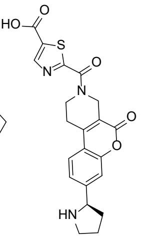
106

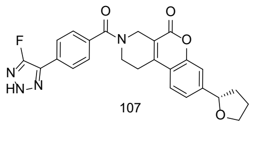
107

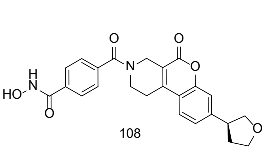
108

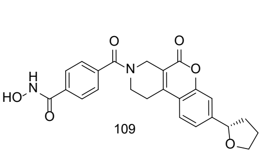
109

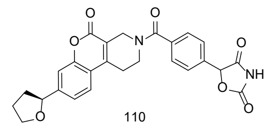
110

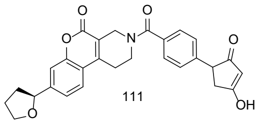
111

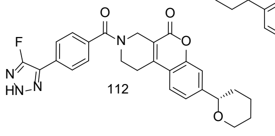
112

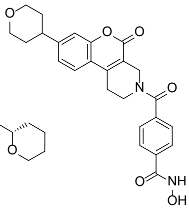
113

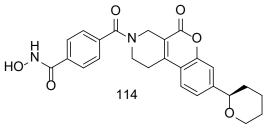
114

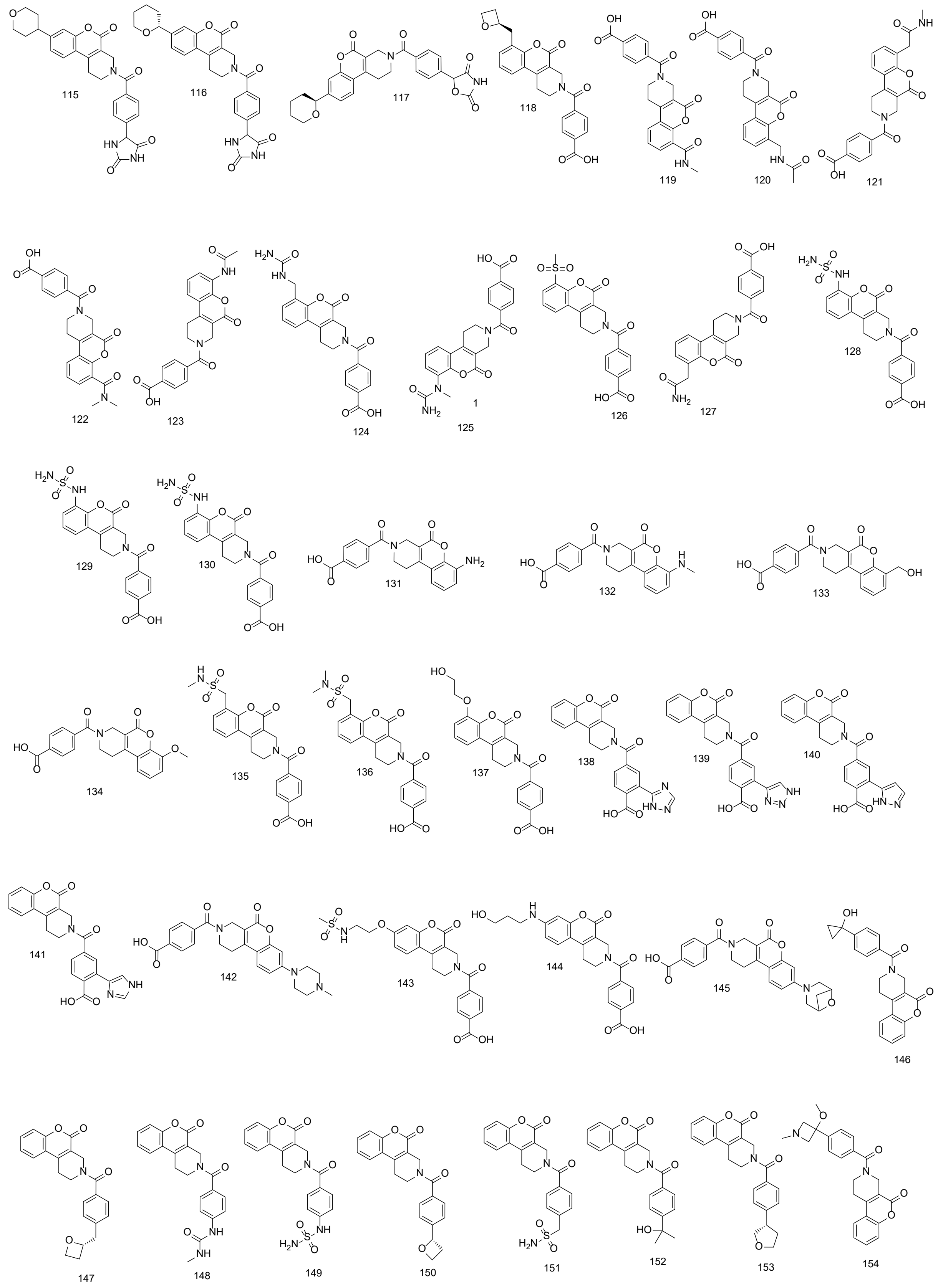

Figure S28. 2D structures of the selected 145 compounds (no 10-154) from the structure-based drug design approach.

Table S1. Computed pharmacokinetic and physicochemical properties of the tricyclic coumarin-based MTHFD2 inhibitors (compounds 1-4) and the 18 selected potential inhibitors of MTHFD2 (compounds 10-27), along with the recommended range of each property (below).

Entry	MW	RO5	logPo/w	logS	PSA	logBB	CNS	logKp	logHERG	logKhsa	%HOA
**1**	422.458	0	1.009	-4.630	135.198	-2.013	-2	-4.635	-6.188	-0.262	64.006
**2**	349.342	0	1.937	-3.727	114.932	-1.441	-2	-4.095	-3.490	-0.308	64.324
**3**	545.052	1	1.872	-4.915	122.575	-1.256	-2	-6.466	-6.558	-0.045	52.212
**4**	608.631	1	2.991	-5.928	130.320	-1.156	-2	-6.183	-6.674	0.295	60.359
**10**	441.455	0	1.156	-4.181	153.956	-2.681	-2	-5.683	-4.003	-0.592	45.581
**11**	556.461	1	3.783	-6.330	111.181	-0.566	1	-5.730	-6.542	0.663	68.483
**12**	419.433	0	2.307	-4.922	134.188	-1.930	-2	-4.744	-3.704	-0.076	61.783
**13**	416.392	0	1.751	-4.888	154.751	-2.274	-2	-5.221	-4.199	-0.210	53.013
**14**	360.412	0	2.280	-3.978	91.974	-0.647	1	-5.183	-6.472	0.323	75.405
**15**	377.439	0	3.468	-5.351	87.706	-1.076	-2	-2.798	-5.697	0.454	94.718
**16**	383.418	0	1.057	-2.555	101.671	-1.065	-2	-3.339	-5.390	-0.814	77.524
**17**	412.459	0	1.346	-2.651	103.384	-1.067	-2	-3.212	-5.266	-0.760	80.098
**18**	362.384	0	2.082	-4.581	105.226	-1.125	-2	-3.204	-5.860	-0.016	83.916
**19**	397.445	0	1.404	-3.162	105.456	-1.407	-2	-3.632	-5.685	-0.635	75.774
**20**	405.449	0	3.504	-4.882	105.759	-1.170	-2	-3.129	-3.595	0.117	81.855
**21**	419.433	0	2.691	-4.802	118.788	-1.300	-2	-3.805	-3.647	-0.113	73.434
**22**	384.406	0	0.664	-3.358	127.972	-1.631	-2	-4.304	-5.262	-0.421	65.328
**23**	446.502	0	2.320	-5.908	131.721	-2.078	-2	-4.757	-5.901	0.276	72.813
**24**	448.474	0	1.401	-4.977	140.452	-2.026	-2	-4.720	-5.815	-0.133	67.523
**25**	469.444	0	1.453	-4.221	142.345	-1.884	-2	-4.768	-4.122	-0.215	62.344
**26**	472.496	0	2.161	-5.304	146.351	-1.616	-2	-4.730	-5.291	0.183	74.599
**27**	486.523	0	2.553	-5.948	146.442	-1.712	-2	-4.733	-5.573	0.342	76.722

MW: Molecular weight (<500).

RO5: Lipinski Rule of Five.

logPo/w: Octanol/water partition coefficient (<5).

logS: Aqueous solubility (-6.5 to 0.5 mol/L).

PSA: Polar surface area (7–200).

logBB: Predicted brain/blood partition coefficient (-3.0 to 1.2).

CNS: Predicted central nervous system activity (-2.0 to 2.0), -2 = completely inactive, -1 = very low activity, 0 = low activity, 1 = medium activity, 2 = completely active.

logKp: Predicted skin permeability (-8.0 to -1.0).

logHERG: Predicted IC_{50} value for blockage of HERG K+ channels (concern < -7).

logKhsa: Prediction of binding to human serum albumin (-1.5 to 1.5).

%HOA: Percent human oral absorption (>80% is high, <25% is poor).

Table S2. SMILES and Glide scores of the selected 145 compounds (Figure S28) in MTHFD2 and MTHFD1.

Compound	SMILES	Glide Score (kcal/mol) in MTHFD2	Glide Score (kcal/mol) in MTHFD1
10	c1cc(C([O-])=O)ccc1C(=O)N(CC2)Cc(c2c34)c(=O)oc3c(CS(=O)(=O)C)ccc4	-9.0	-5.3
11	FC(F)(F)C([O-])(C(F)(F)F)c1ccc(cc1)C(=O)N(CC2)Cc(c2c34)c(=O)oc3cc(cc4)[C@@H]5C[NH2+]CCO5	-8.8	-6.1
12	c1cc(C([O-])=O)ccc1C(=O)N(CC2)Cc(c2c34)c(=O)oc3c(ccc4)[C@@H]5C[C@@H](C5)O	-9.0	-5.8
13	[nH]1nncc1-c(c2C([O-])=O)cc(cc2)C(=O)N(CC3)Cc(c3c45)c(=O)oc4cccc5	-9.4	-6.5
14	C1CC1(N)c(cc2)ccc2C(=O)N(CC3)Cc(c3c45)c(=O)oc4cccc5	-8.2	-5.8
15	CC(C)(O)Cc(cc1)ccc1C(=O)N(CC2)Cc(c2c34)c(=O)oc3cccc4	-8.0	-6.0
16	CS(=O)(=O)c(cc1)ccc1C(=O)N(CC2)Cc(c2c34)c(=O)oc3cccc4	-8.2	-5.5
17	CN(C)S(=O)(=O)c(cc1)ccc1C(=O)N(CC2)Cc(c2c34)c(=O)oc3cccc4	-7.7	-5.3
18	CC(=O)Nc(cc1)ccc1C(=O)N(CC2)Cc(c2c34)c(=O)oc3cccc4	-7.6	-5.5
19	CS(=O)(=O)Cc(cc1)ccc1C(=O)N(CC2)Cc(c2c34)c(=O)oc3cccc4	-8.3	-5.7
20	[O-]C(=O)C(C)(C)Cc(cc1)ccc1C(=O)N(CC2)Cc(c2c34)c(=O)oc3cccc4	-8.0	-6.0
21	c1cc(C([O-])=O)ccc1C(=O)N(CC2)Cc(c2c34)c(=O)oc3c(ccc4)[C@@H]5CCCO5	-8.6	-6.0
22	c1cc(S(=O)(=O)N)ccc1C(=O)N(CC2)Cc(c2c34)c(=O)oc3cccc4	-8.1	-5.3
23	[O-]NC(=O)c1ccc(cc1)C(=O)N(CC2)Cc(c2c34)c(=O)oc3cc(cc4)C5CCCCC5	-9.7	-5.7
24	[O-]NC(=O)c1ccc(cc1)C(=O)N(CC2)Cc(c2c34)c(=O)oc3cc(cc4)[C@H]5CCCOC5	-9.8	-7.0
25	[O-]NC(=O)C(F)(F)c(cc1)ccc1C(=O)N(CC2)Cc(c2c34)c(=O)oc3cc(cc4)C5C[NH2+]C5	-8.7	-5.7
26	[N-]1C(=O)C[C@H](C1=O)c(cc2)ccc2C(=O)N(CC3)Cc(c3c45)c(=O)oc4cc(cc5)[C@@H]6CCCO6	-10.3	-7.0
27	[N-]1C(=O)C[C@H](C1=O)c(cc2)ccc2C(=O)N(CC3)Cc(c3c45)c(=O)oc4cc(cc5)[C@@H]6CCCCO6	-10.2	-7.4
28	c1cc(C([O-])=O)ccc1C(=O)N(CC2)Cc(c2c34)c(=O)oc3cc(cc4)[C@H]5CCN5	-9.7	-7.3
29	c1cc(C([O-])=O)ccc1C(=O)N(CC2)Cc(c2c34)c(=O)oc3cc(cc4)[C@@H]5CCN5	-9.5	-7.1
30	[O-]NC(=O)c1ccc(cc1)C(=O)N(CC2)Cc(c2c34)c(=O)oc3cc(cc4)N5CCC5	-9.3	-5.7
31	c1o[n-]c(=O)c1-c(cc2)ccc2C(=O)N(CC3)Cc(c3c45)c(=O)oc4cc(cc5)[C@H]6CCN6	-10.3	-6.9
32	c1o[n-]c(=O)c1-c(cc2)ccc2C(=O)N(CC3)Cc(c3c45)c(=O)oc4cc(cc5)[C@@H]6CCN6	-10.2	6.8
33	[N-]1C(=O)N[C@H](C1=O)c(cc2)ccc2C(=O)N(CC3)Cc(c3c45)c(=O)oc4cc(cc5)[C@H]6CC[NH2+]6	-9.4	-6.9
34	[nH]1ccn(c1=O)-c(cc2)ccc2C(=O)N(CC3)Cc(c3c45)c(=O)oc4cc(cc5)[C@@H]6CCN6	-9.3	-6.0
35	[O-]NC(=O)C(F)(F)c(cc1)ccc1C(=O)N(CC2)Cc(c2c34)c(=O)oc3cc(cc4)[C@@H]5CC[NH2+]5	-8.5	-5.6
36	CONC(=O)c1ccc(cc1)C(=O)N(CC2)Cc(c2c34)c(=O)oc3cc(cc4)[C@H]5CCN5	-9.6	-6.9
37	CC(=O)NOc(cc1)ccc1C(=O)N(CC2)Cc(c2c34)c(=O)oc3cc(cc4)[C@@H]5CCN5	-9.4	-6.3
38	C1C[C@@H](C([O-])=O)CC[C@@H]1C(=O)N(CC2)Cc(c2c34)c(=O)oc3cc(cc4)[C@@H]5CCN5	-9.3	-7.2

39	[O-]C(=O)[C@@H](C1)C[C@@H]([C@@H]12)[C@H]2C(=O)N(CC3)Cc(c3c45)c(=O)oc4cc(cc5)[C@@H]6CCN6	-9.3	-7.2
40	c1nc(C([O-])=O)ccc1C(=O)N(CC2)Cc(c2c34)c(=O)oc3cc(cc4)[C@H]5CCN5	-9.6	-7.2
41	c1nc(C([O-])=O)ccc1C(=O)N(CC2)Cc(c2c34)c(=O)oc3cc(cc4)[C@@H]5CCN5	-9.4	-7.2
42	[O-]C(=O)c1ccc(nc1)C(=O)N(CC2)Cc(c2c34)c(=O)oc3cc(cc4)[C@H]5CCN5	-9.5	-7.0
43	[O-]C(=O)c1ccc(nc1)C(=O)N(CC2)Cc(c2c34)c(=O)oc3cc(cc4)[C@@H]5CCN5	-9.4	-7.0
44	[O-]C(=O)c1cnc(nc1)C(=O)N(CC2)Cc(c2c34)c(=O)oc3cc(cc4)[C@H]5CCN5	-9.5	-5.9
45	n1cc(C([O-])=O)ncc1C(=O)N(CC2)Cc(c2c34)c(=O)oc3cc(cc4)[C@H]5CCN5	-9.4	-5.8
46	n1cc(C([O-])=O)ncc1C(=O)N(CC2)Cc(c2c34)c(=O)oc3cc(cc4)[C@@H]5CCN5	-9.3	-5.7
47	[O-]C(=O)c(s1)ncc1C(=O)N(CC2)Cc(c2c34)c(=O)oc3cc(cc4)[C@H]5CCN5	-9.3	-6.8
48	[O-]NC(=O)c1ccc(cc1)C(=O)N(CC2)Cc(c2c34)c(=O)oc3cc(cc4)C5CCC5	-9.8	-6.9
49	n1nc(C([O-])=O)ccc1C(=O)N(CC2)Cc(c2c34)c(=O)oc3cc(cc4)C5CCCCC5	-8.5	-4.6
50	c1cc(C([O-])=O)ccc1C(=O)N(CC2)Cc(c2c34)c(=O)oc3cc(cc4)[C@@H]5COCC[NH2+]5	-7.4	-6.0
51	[O-]NC(=O)c1ccc(cc1)C(=O)N(CC2)Cc(c2c34)c(=O)oc3cc(cc4)N5CCOCC5	-9.2	-7.0
52	[O-]NC(=O)c1ccc(cc1)C(=O)N(CC2)Cc(c2c34)c(=O)oc3cc(cc4)[C@@H]5COCC[NH2+]5	-9.8	-7.2
53	[O-]NC(=O)c1ccc(cc1)C(=O)N(CC2)Cc(c2c34)c(=O)oc3cc(cc4)[C@@H]5C[NH2+]CCO5	-10.7	-5.9
54	[N-]1C(=O)C[C@H](C1=O)c(cc2)ccc2C(=O)N(CC3)Cc(c3c45)c(=O)oc4cc(cc5)[C@@H]6C[NH2+]CCO6	-10.1	-6.7
55	[N-]1C(=O)N[C@H](C1=O)c(cc2)ccc2C(=O)N(CC3)Cc(c3c45)c(=O)oc4cc(cc5)N6CCOCC6	-9.4	-6.7
56	[N-]1C(=O)N[C@H](C1=O)c(cc2)ccc2C(=O)N(CC3)Cc(c3c45)c(=O)oc4cc(cc5)[C@H]6C[NH2+]CCO6	-9.3	-4.7
57	[N-]1C(=O)N[C@H](C1=O)c(cc2)ccc2C(=O)N(CC3)Cc(c3c45)c(=O)oc4cc(cc5)[C@@H]6C[NH2+]CCO6	-10.2	-6.9
58	O=c1c(=O)c([O-])c1-c(cc2)ccc2C(=O)N(CC3)Cc(c3c45)c(=O)oc4cc(cc5)[C@@H]6CNCCO6	-10.2	-7.5
59	c1cc(P([O-])(=O)O)ccc1C(=O)N(CC2)Cc(c2c34)c(=O)oc3cc(cc4)[C@H]5C[NH2+]CCO5	-9.1	-5.5
60	c1cc(P([O-])=O)ccc1C(=O)N(CC2)Cc(c2c34)c(=O)oc3cc(cc4)[C@@H]5CNCCO5	-11.0	-7.7
61	[O-]C(=O)[C@H](C1)C[C@@H]([C@@H]12)[C@H]2C(=O)N(CC3)Cc(c3c45)c(=O)oc4cc(cc5)[C@H]6COCCN6	-9.5	-5.7
62	[O-]C(=O)c1cnc(nc1)C(=O)N(CC2)Cc(c2c34)c(=O)oc3cc(cc4)[C@@H]5CNCCO5	-10.0	-6.8
63	[N-]1C(=O)N[C@H](C1=O)c(cc2)ccc2C(=O)N(CC3)Cc(c3c45)c(=O)oc4cc(cc5)C6COC6	-9.5	-6.5
64	c1cc(C([O-])=O)ccc1C(=O)N(CC2)Cc(c2c34)c(=O)oc3cc(cc4)[C@@H]5CNCC[NH2+]5	-8.1	-6.2
65	[N-]1C(=O)N[C@H](C1=O)c(cc2)ccc2C(=O)N(CC3)Cc(c3c45)c(=O)oc4cc(cc5)[C@@H]6C[NH2+]CCN6	-10.1	-6.9
66	[N-]1C(=O)O[C@H](C1=O)c(cc2)ccc2C(=O)N(CC3)Cc(c3c45)c(=O)oc4cc(cc5)N6CC[NH2+]CC6	-9.2	-4.8
67	c1cc(P([O-])(=O)O)ccc1C(=O)N(CC2)Cc(c2c34)c(=O)oc3cc(cc4)[C@H]5C[NH2+]CCN5	-8.6	-5.6
68	O=C1NC(=O)C([O-])=C1c(cc2)ccc2C(=O)N(CC3)Cc(c3c45)c(=O)oc4cc(cc5)[C@H]6CCCCN6	-9.7	-7.2
69	O=C1NC(=O)C([O-])=C1c(cc2)ccc2C(=O)N(CC3)Cc(c3c45)c(=O)oc4cc(cc5)[C@@H]6CCCCN6	-9.7	-6.7
70	[O-]NC(=O)c1ccc(cc1)C(=O)N(CC2)Cc(c2c34)c(=O)oc3cc(cc4)C5CC[NH2+]CC5	-9.3	-4.9
71	[O-]NC(=O)c1ccc(cc1)C(=O)N(CC2)Cc(c2c34)c(=O)oc3cc(cc4)[C@@H]5CCCC[NH2+]5	-9.3	-6.9
72	N1C(=O)C[C@H](C1=O)c(cc2)ccc2C(=O)N(CC3)Cc(c3c45)c(=O)oc4cc(cc5)[C@H]6CCCCN6	-9.8	-7.1
73	c1o[n-]c(=O)c1-c(cc2)ccc2C(=O)N(CC3)Cc(c3c45)c(=O)oc4cc(cc5)[C@@H]6CCCCN6	-10.2	-6.8
74	[N-]1C(=O)N[C@H](C1=O)c(cc2)ccc2C(=O)N(CC3)Cc(c3c45)c(=O)oc4cc(cc5)C6CC[NH2+]CC6	-9.4	-6.6
75	[N-]1C(=O)N[C@H](C1=O)c(cc2)ccc2C(=O)N(CC3)Cc(c3c45)c(=O)oc4cc(cc5)[C@H]6CCC[NH2+]C6	-9.4	-6.1
76	[N-]1C(=O)N[C@H](C1=O)c(cc2)ccc2C(=O)N(CC3)Cc(c3c45)c(=O)oc4cc(cc5)[C@@H]6CCCC[NH2+]6	-9.5	-6.5
77	[N-]1C(=O)O[C@H](C1=O)c(cc2)ccc2C(=O)N(CC3)Cc(c3c45)c(=O)oc4cc(cc5)[C@@H]6CCCCN6	-10.3	-6.8
78	C1OCC1(O)c(cc2)ccc2C(=O)N(CC3)Cc(c3c45)c(=O)oc4cc(cc5)[C@H]6CCCCN6	-9.5	-7.0
79	C1OCC1(O)c(cc2)ccc2C(=O)N(CC3)Cc(c3c45)c(=O)oc4cc(cc5)[C@@H]6CCCCN6	-9.5	-6.5
80	CONC(=O)c1ccc(cc1)C(=O)N(CC2)Cc(c2c34)c(=O)oc3cc(cc4)[C@H]5CCC[NH2+]C5	-8.3	-4.8
81	c1cc(P([O-])([O-])=O)ccc1C(=O)N(CC2)Cc(c2c34)c(=O)oc3cc(cc4)[C@H]5CCCCN5	-10.4	-7.4
82	c1cc(P([O-])([O-])=O)ccc1C(=O)N(CC2)Cc(c2c34)c(=O)oc3cc(cc4)[C@@H]5CCCCN5	-10.4	-7.1
83	C1C[C@@H](C([O-])=O)CC[C@@H]1C(=O)N(CC2)Cc(c2c34)c(=O)oc3cc(cc4)[C@H]5CCCCN5	-9.5	-7.2

84	[O-]C(=O)[C@@H]1[C@H]([C@H]12)C[C@@H](C2)C(=O)N(CC3)Cc(c3c45)c(=O)oc4cc(cc5)[C@@H]6CCCCN6	-9.4	-7.1
85	c1nc(C([O-])=O)ccc1C(=O)N(CC2)Cc(c2c34)c(=O)oc3cc(cc4)[C@H]5CCCCN5	-9.5	-6.6
86	c1nc(C([O-])=O)ccc1C(=O)N(CC2)Cc(c2c34)c(=O)oc3cc(cc4)[C@@H]5CCCCN5	-9.7	-7.1
87	[O-]C(=O)c1ccc(nc1)C(=O)N(CC2)Cc(c2c34)c(=O)oc3cc(cc4)[C@H]5CCCCN5	-9.7	-7.2
88	n1cc(C([O-])=O)ncc1C(=O)N(CC2)Cc(c2c34)c(=O)oc3cc(cc4)[C@@H]5CCCCN5	-9.4	-6.8
89	[O-]C(=O)c(s1)ccc1C(=O)N(CC2)Cc(c2c34)c(=O)oc3cc(cc4)[C@H]5CCCCN5	-9.6	-6.7
90	[O-]C(=O)c(s1)ccc1C(=O)N(CC2)Cc(c2c34)c(=O)oc3cc(cc4)[C@@H]5CCCCN5	-9.7	-7.3
91	[O-]C(=O)c1cnc(s1)C(=O)N(CC2)Cc(c2c34)c(=O)oc3cc(cc4)[C@H]5CCCCN5	-9.5	-6.8
92	[O-]C(=O)c1cnc(s1)C(=O)N(CC2)Cc(c2c34)c(=O)oc3cc(cc4)[C@@H]5CCCCN5	-9.5	-6.8
93	[O-]NC(=O)c1ccc(cc1)C(=O)N(CC2)Cc(c2c34)c(=O)oc3cc(cc4)[C@H]5CCC[NH2+]5	-9.5	-6.8
94	c1o[n-]c(=O)c1-c(cc2)ccc2C(=O)N(CC3)Cc(c3c45)c(=O)oc4cc(cc5)[C@@H]6CCCN6	-10.3	-6.9
95	N1C(=O)N[C@H](C1=O)c(cc2)ccc2C(=O)N(CC3)Cc(c3c45)c(=O)oc4cc(cc5)[C@@H]6CCCN6	-9.9	-6.3
96	[N-]1C(=O)N[C@H](C1=O)c(cc2)ccc2C(=O)N(CC3)Cc(c3c45)c(=O)oc4cc(cc5)[C@H]6CCC[NH2+]6	-9.3	-6.8
97	FC(F)(F)[C@H](O)c(cc1)ccc1C(=O)N(CC2)Cc(c2c34)c(=O)oc3cc(cc4)[C@@H]5CCCN5	-9.4	-6.3
98	FC(F)(F)[C@@H](O)c(cc1)ccc1C(=O)N(CC2)Cc(c2c34)c(=O)oc3cc(cc4)[C@@H]5CCCN5	-9.6	-6.3
99	c1cc(P([O-])=O)ccc1C(=O)N(CC2)Cc(c2c34)c(=O)oc3cc(cc4)[C@@H]5CCCN5	-10.2	-7.2
100	[O-]C(=O)[C@H](C1)C[C@@H]([C@@H]12)[C@H]2C(=O)N(CC3)Cc(c3c45)c(=O)oc4cc(cc5)[C@H]6CCCN6	-9.8	-6.0
101	[O-]C(=O)[C@@H](C1)C[C@@H]([C@@H]12)[C@H]2C(=O)N(CC3)Cc(c3c45)c(=O)oc4cc(cc5)[C@H]6CCCCN6	-9.5	-6.1
102	[O-]C(=O)[C@@H]1[C@H]([C@H]12)C[C@@H](C2)C(=O)N(CC3)Cc(c3c45)c(=O)oc4cc(cc5)[C@H]6CCCN6	-9.5	-7.2
103	[O-]C(=O)c1ccc(nc1)C(=O)N(CC2)Cc(c2c34)c(=O)oc3cc(cc4)[C@@H]5CCCN5	-9.6	-7.3
104	[O-]C(=O)c1cnc(nc1)C(=O)N(CC2)Cc(c2c34)c(=O)oc3cc(cc4)[C@H]5CCCN5	-9.4	-6.8
105	n1cc(C([O-])=O)ncc1C(=O)N(CC2)Cc(c2c34)c(=O)oc3cc(cc4)[C@@H]5CCCN5	-9.4	-6.2
106	[O-]C(=O)c1cnc(s1)C(=O)N(CC2)Cc(c2c34)c(=O)oc3cc(cc4)[C@H]5CCCN5	-9.4	-7.2
107	n1[n-]nc(F)c1-c(cc2)ccc2C(=O)N(CC3)Cc(c3c45)c(=O)oc4cc(cc5)[C@@H]6CCCO6	-8.7	-5.2
108	[O-]NC(=O)c1ccc(cc1)C(=O)N(CC2)Cc(c2c34)c(=O)oc3cc(cc4)[C@@H]5CCOC5	-9.3	-6.1
109	[O-]NC(=O)c1ccc(cc1)C(=O)N(CC2)Cc(c2c34)c(=O)oc3cc(cc4)[C@@H]5CCCO5	-9.3	-5.8
110	N1C(=O)O[C@H](C1=O)c(cc2)ccc2C(=O)N(CC3)Cc(c3c45)c(=O)oc4cc(cc5)[C@@H]6CCCO6	-9.5	-6.8
111	O=C1C=C(O)C[C@H]1c(cc2)ccc2C(=O)N(CC3)Cc(c3c45)c(=O)oc4cc(cc5)[C@@H]6CCCO6	-9.7	-6.0
112	n1[n-]nc(F)c1-c(cc2)ccc2C(=O)N(CC3)Cc(c3c45)c(=O)oc4cc(cc5)[C@@H]6CCCCO6	-8.7	-6.3
113	[O-]NC(=O)c1ccc(cc1)C(=O)N(CC2)Cc(c2c34)c(=O)oc3cc(cc4)C5CCOCC5	-9.8	-6.3
114	[O-]NC(=O)c1ccc(cc1)C(=O)N(CC2)Cc(c2c34)c(=O)oc3cc(cc4)[C@H]5CCCCO5	-9.3	-6.7
115	[N-]1C(=O)N[C@H](C1=O)c(cc2)ccc2C(=O)N(CC3)Cc(c3c45)c(=O)oc4cc(cc5)C6CCOCC6	-10.0	-6.8
116	[N-]1C(=O)N[C@H](C1=O)c(cc2)ccc2C(=O)N(CC3)Cc(c3c45)c(=O)oc4cc(cc5)[C@H]6CCCCO6	-10.9	-6.9
117	N1C(=O)O[C@H](C1=O)c(cc2)ccc2C(=O)N(CC3)Cc(c3c45)c(=O)oc4cc(cc5)[C@@H]6CCCCO6	-9.5	-6.7
118	c1cc(C([O-])=O)ccc1C(=O)N(CC2)Cc(c2c34)c(=O)oc3c(ccc4)C[C@@H]5CCO5	-8.9	-5.5
119	CNC(=O)c(ccc1)c2oc(=O)c(c3c12)CN(CC3)C(=O)c4ccc(C([O-])=O)cc4	-8.8	-5.7
120	CC(=O)NCc(ccc1)c2oc(=O)c(c3c12)CN(CC3)C(=O)c4ccc(C([O-])=O)cc4	-10.2	-5.8
121	CNC(=O)Cc(ccc1)c2oc(=O)c(c3c12)CN(CC3)C(=O)c4ccc(C([O-])=O)cc4	-9.8	-5.6
122	CN(C)C(=O)c(ccc1)c2oc(=O)c(c3c12)CN(CC3)C(=O)c4ccc(C([O-])=O)cc4	-8.7	-5.6
123	CC(=O)Nc(ccc1)c2oc(=O)c(c3c12)CN(CC3)C(=O)c4ccc(C([O-])=O)cc4	-9.6	-5.7
124	c1cc(C([O-])=O)ccc1C(=O)N(CC2)Cc(c2c34)c(=O)oc3c(ccc4)CNC(=O)N	-10.3	-5.6
125	c1cc(C([O-])=O)ccc1C(=O)N(CC2)Cc(c2c34)c(=O)oc3c(ccc4)N(C)C(=O)N	-9.4	-5.8
126	c1cc(C([O-])=O)ccc1C(=O)N(CC2)Cc(c2c34)c(=O)oc3c(S(=O)(=O)C)ccc4	-8.7	-5.8
127	c1ccc(CC(=O)N)c2oc(=O)c(c3c12)CN(CC3)C(=O)c4ccc(C([O-])=O)cc4	-9.8	-5.7
128	c1cc(C([O-])=O)ccc1C(=O)N(CC2)Cc(c2c34)c(=O)oc3c(ccc4)NS(=O)(=O)N	-8.9	-5.4

129	c1cc(C([O-])=O)ccc1C(=O)N(CC2)Cc(c2c34)c(=O)oc3c(ccc4)NS(=O)(=O)[NH-]	-10.1	-5.6
130	c1cc(C([O-])=O)ccc1C(=O)N(CC2)Cc(c2c34)c(=O)oc3c(ccc4)[N-]S(=O)(=O)N	-9.6	-5.6
131	c1cc(C([O-])=O)ccc1C(=O)N(CC2)Cc(c2c34)c(=O)oc3c(N)ccc4	-8.7	-6.0
132	c1cc(C([O-])=O)ccc1C(=O)N(CC2)Cc(c2c34)c(=O)oc3c(NC)ccc4	-8.8	-5.9
133	c1cc(C([O-])=O)ccc1C(=O)N(CC2)Cc(c2c34)c(=O)oc3c(CO)ccc4	-9.2	-5.8
134	c1cc(C([O-])=O)ccc1C(=O)N(CC2)Cc(c2c34)c(=O)oc3c(OC)ccc4	-8.7	-5.9
135	c1cc(C([O-])=O)ccc1C(=O)N(CC2)Cc(c2c34)c(=O)oc3c(ccc4)CS(=O)(=O)NC	-9.1	-5.2
136	c1cc(C([O-])=O)ccc1C(=O)N(CC2)Cc(c2c34)c(=O)oc3c(ccc4)CS(=O)(=O)N(C)C	-9.8	-5.5
137	c1cc(C([O-])=O)ccc1C(=O)N(CC2)Cc(c2c34)c(=O)oc3c(ccc4)OCCO	-8.7	-5.9
138	[nH]1ncnc1-c(c2C([O-])=O)cc(cc2)C(=O)N(CC3)Cc(c3c45)c(=O)oc4cccc5	-9.2	-6.8
139	n1n[nH]cc1-c(c2C([O-])=O)cc(cc2)C(=O)N(CC3)Cc(c3c45)c(=O)oc4cccc5	-8.7	-6.9
140	[nH]1nccc1-c(c2C([O-])=O)cc(cc2)C(=O)N(CC3)Cc(c3c45)c(=O)oc4cccc5	-8.6	-6.1
141	[nH+]1c[nH]cc1-c(c2C([O-])=O)cc(cc2)C(=O)N(CC3)Cc(c3c45)c(=O)oc4cccc5	-8.3	-6.1
142	c1cc(C([O-])=O)ccc1C(=O)N(CC2)Cc(c2c34)c(=O)oc3cc(cc4)N(CC5)CCN5C	-9.0	-7.2
143	c1cc(C([O-])=O)ccc1C(=O)N(CC2)Cc(c2c34)c(=O)oc3cc(cc4)OCCNS(=O)(=O)C	-8.8	-5.2
144	c1cc(C([O-])=O)ccc1C(=O)N(CC2)Cc(c2c34)c(=O)oc3cc(cc4)NCCCO	-9.1	-5.9
145	c1cc(C([O-])=O)ccc1C(=O)N(CC2)Cc(c2c34)c(=O)oc3cc(cc4)N(C5)C[C@@H](O6)C[C@H]56	-8.6	-5.9
146	C1CC1(O)c(cc2)ccc2C(=O)N(CC3)Cc(c3c45)c(=O)oc4cccc5	-7.1	-5.9
147	O1CC[C@H]1Cc(cc2)ccc2C(=O)N(CC3)Cc(c3c45)c(=O)oc4cccc5	-7.7	-5.7
148	CNC(=O)Nc(cc1)ccc1C(=O)N(CC2)Cc(c2c34)c(=O)oc3cccc4	-7.4	-5.1
149	NS(=O)(=O)[N-]c(cc1)ccc1C(=O)N(CC2)Cc(c2c34)c(=O)oc3cccc4	-8.6	-6.1
150	O1CC[C@H]1c(cc2)ccc2C(=O)N(CC3)Cc(c3c45)c(=O)oc4cccc5	-7.2	-5.5
151	NS(=O)(=O)Cc(cc1)ccc1C(=O)N(CC2)Cc(c2c34)c(=O)oc3cccc4	-8.2	-5.6
152	CC(C)(O)c(cc1)ccc1C(=O)N(CC2)Cc(c2c34)c(=O)oc3cccc4	-7.8	-5.4
153	C1OCC[C@H]1c(cc2)ccc2C(=O)N(CC3)Cc(c3c45)c(=O)oc4cccc5	-7.5	5.4
154	CN(C1)CC1(OC)c(cc2)ccc2C(=O)N(CC3)Cc(c3c45)c(=O)oc4cccc5	-8.2	-6.7
